# *Nido*-Carborane Derivatives of (*S*)-Ornithine and (*S*)-Lysine as Potential Boron Delivery Agents: Synthesis and In Vitro Evaluation

**DOI:** 10.3390/ijms26178560

**Published:** 2025-09-03

**Authors:** Dmitry A. Gruzdev, Galina L. Levit, Vera V. Musiyak, Angelina A. Telegina, Ilya N. Ganebnykh, Marina A. Ezhikova, Mikhail I. Kodess, Olga I. Solovieva, Tatiana Y. Gusel’nikova, Ivan A. Razumov, Victor P. Krasnov

**Affiliations:** 1 Postovsky Institute of Organic Synthesis, Russian Academy of Sciences (Ural Branch), Ekaterinburg 620066, Russia; ca512@ios.uran.ru (G.L.L.); musiyak@ios.uran.ru (V.V.M.); telegina@ios.uran.ru (A.A.T.); ing@ios.uran.ru (I.N.G.); ema@ios.uran.ru (M.A.E.); nmr@ios.uran.ru (M.I.K.); ca@ios.uran.ru (V.P.K.); 2 Institute of Cytology and Genetics, Russian Academy of Sciences (Siberian Branch), Novosibirsk 630090, Russia; solovieva@bionet.nsc.ru (O.I.S.); razumov@bionet.nsc.ru (I.A.R.); 3 Department of Physics, Novosibirsk State University, Novosibirsk 630090, Russia; gtya18@gmail.com

**Keywords:** *nido*-carborane, lysine, ornithine, toxicity assessment, boron uptake assay

## Abstract

Derivatives of natural amino acids are selectively absorbed by many types of tumour cells. This makes the use of amino acids, especially polyfunctional ones, attractive as a basis in the design of low-toxicity agents for targeted boron delivery for boron neutron capture therapy (BNCT) of tumours. We synthesized a series of new (*S*)-ornithine and (*S*)-lysine derivatives containing a 7,8-dicarba-*nido*-undecaborane (*nido*-carborane) residue attached to the amino group in the side chain or alpha position. The MTT assay demonstrated moderate cytotoxicity of the lysine and ornithine derivatives containing a *nido*-carborane residue in the side chain. It has been found that sodium salt of *N*^ε^-(*nido*-carboran-7-yl)acetyl-(*S*)-lysine is capable of accumulation by MDA-MB-231 (human breast carcinoma) and SK-Mel 28 (human melanoma) cell lines, providing a boron concentration of up to 0.67 µg/10^6^ cells in in vitro experiments. This (*S*)-lysine derivative containing a *nido*-carborane residue in the side chain can be considered as a promising compound for in-depth study in vivo experiments aimed at designing an efficient boron delivery agent for BNCT.

## 1. Introduction

Natural amino acids are widely used in the design of prodrugs and medicinal agents for targeted delivery [1,2,3]. The introduction of fragments of polyfunctional amino acids allows optimizing the pharmacokinetic parameters of bioactive compounds and reducing their toxicity. In particular, the structural fragment of (*S*)-lysine has been used in the synthesis of water-soluble derivatives of amphetamine [4,5,6,7], diazepam [8,9,10,11,12], midazolam [13,14], derivatives of ketoprofen [15,16,17] and methotrexate [18], which are capable of overcoming the blood–brain barrier; as well as efficient antitumour [19,20,21,22,23,24,25] and antibacterial agents [26,27,28,29]. Many tumour cells are characterized by high activity of ornithine decarboxylase involved in the biosynthesis of polyamines [30,31,32]. This makes it possible to design new (*S*)-ornithine-based pharmaceuticals for cancer therapy and diagnostics [19,33,34,35,36,37,38].

Treatment of tumour diseases is one of the most important problems of modern medicine. Despite the successes and development of therapeutic methods, the incidence of cancer remains high [39]. One of the efficient modern approaches to cancer treatment is boron neutron capture therapy (BNCT). This method is based on the combined use of boron-containing agents of different nature and irradiation with thermal or epithermal neutrons [40,41,42,43,44,45,46]. An important requirement that guarantees the possibility of using the method is the selective delivery of boron compounds to tumour cells. One of the ways to design pharmaceuticals for targeted delivery of boron is to obtain conjugates of natural molecules containing residues of polyhedral boron-containing molecules, primarily dicarba-*closo*-dodecaborane and dicarba-*nido*-undecaborane (carboranes), as well as dodecaborate [47,48,49,50,51,52].

We have previously shown that carborane-containing folic acid conjugates have low toxicity and are capable of selective accumulation in U87 MG human glioblastoma cells (up to 7.0 µg B/10^6^ cells) [53]. There are several examples of tumour-selective boron delivery agents, for example, carborane derivatives of prostate specific membrane antigen (PSMA) [54,55], glucose [56,57], and short peptides [58,59].

Natural polyfunctional amino acids are an important class of tumour-tropic molecules, since cancer cells are characterized by high activity of metabolic processes and actively use amino acids in their vital functions [60,61].

A series of lysine derivatives containing *closo*-carborane moieties were synthesized in the context of the search for efficient agents for targeted boron delivery in BNCT [62]. Modification of lysine residues using carborane- and dodecaborane-containing building blocks made it possible to prepare boron-containing analogues of neuropeptide Y [63,64,65,66,67,68], [Tyr^3^]-octreotate [69], transferrin derivatives [70], ghrelin receptor agonists [71], and other peptides [72,73,74]. A number of boron-containing ligands of PSMA, which were capable of accumulating in cells, were obtained by acylation of the amino group in the side chain of lysine with carborane-based acids [54,55]. Lysine was used as a linker fragment in the design of a monodisperse closomer drug delivery system [75], derivatives of cell-membrane penetrating peptides [76,77], as well as modified boron carbide nanoparticles [78]. Previously, we demonstrated the possibility of synthesizing lysine derivatives containing one or two *closo*-carborane residues [79,80], and also prepared a bis-*nido*-carboranyl derivative of the tumour-tropic KRGD peptide with low cytotoxicity [81].

This paper describes the synthetic routes for simple in structure water-soluble *nido*-carborane-containing derivatives of (*S*)-lysine and (*S*)-ornithine with free carboxyl and amino groups in the alpha position or in the side chain, and also provides an assessment of the cytotoxicity of some of the obtained compounds and their accumulation in cells.

## 2. Results and Discussion

### 2.1. Synthesis and Characterization of the Obtained Compounds

(*closo*-Carboran-1-yl)acetic acid (**1**) was used as the starting carborane-containing building block. Previously, we have shown that, starting from compound **1**, carborane conjugates with natural molecules such as adenine [82], amino acids [83], and folic acid [53] can be easily synthesized. To obtain derivatives of (*S*)-lysine and (*S*)-ornithine containing one residue of *nido*-carborane in the side chain or at the α-amino group, we used an approach based on acylation of *tert*-butyl *N*^α^-Boc or *N*^ω^-Boc amino esters followed by deboronation and removal of protecting groups in one step. We have previously demonstrated that the simultaneous removal of several protecting groups at the last stage of synthesis is convenient for obtaining carboranyl derivatives of polyfunctional amino acids [84,85], as well as lysine and ornithine derivatives with substituents in the side chain [79,86].

**Scheme 1:**
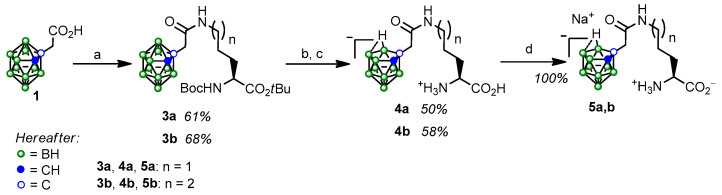
Synthesis of *N*^ω^-(*nido*-carboran-7-yl)acetyl derivatives of (*S*)-ornithine and (*S*)-lysine. (a) Compound **2a** (or **2b**), EtOCOCl, NMM, PhNEt_2_, THF, −12 °C to rt, 24 h. (b) CsF (3 equiv.), EtOH, Δ, 16 h. (c) HCl conc., 1,4-dioxane, rt, 3 h. (d) Na_2_CO_3_, H_2_O/EtOH, rt, 0.5 h.

**Figure 1:**
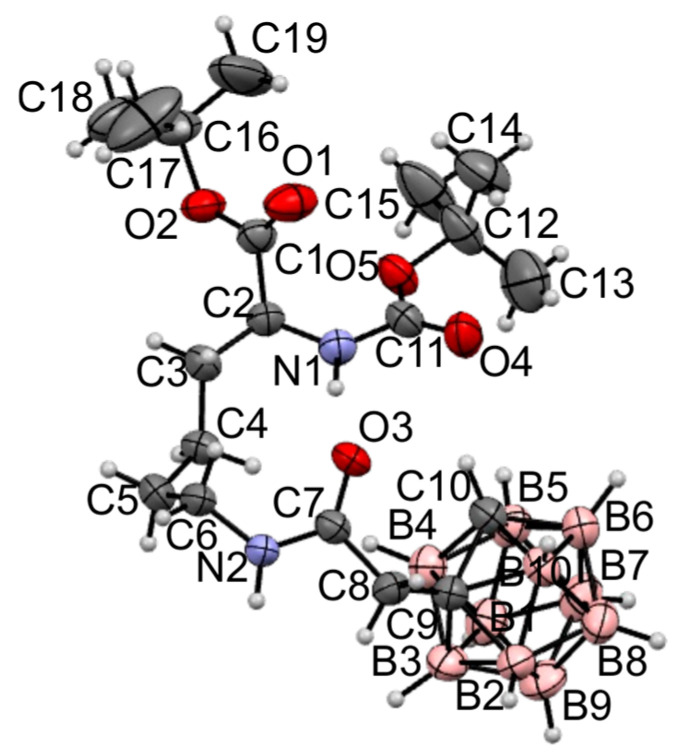
ORTEP structure of compound **3b** (thermal ellipsoids of 50% probability).

Amino esters *N*^α^-Boc-Orn-O*t*Bu (**2a**) and *N*^α^-Boc-Lys-O*t*Bu (**2b**) obtained from the corresponding *N*^ω^-Cbz derivatives by analogy with known procedures [87,88,89] were coupled to acid **1** using the mixed anhydride method (Scheme 1). Amides **3a** and **3b** were isolated in 61 and 68% yields, respectively. The structure of compound **3b**, determined by X-ray diffraction, is shown in Figure 1.

Treatment of dicarba-*closo*-dodecaborane derivatives, which are highly lipophilic compounds, with cesium fluoride or other fluorides is a convenient method for converting them into the corresponding dicarba-*nido*-undecaborane derivatives [90,91,92], which in the form of salts are often characterized by high solubility in water. We have previously used CsF treatment to prepare a number of *nido*-carborane derivatives, including planar-chiral ones [82,83,93]. Refluxing the protected *closo*-carborane derivatives **3a**,**b** with excess CsF in ethanol followed by removal of protecting groups under acidic conditions smoothly led to the corresponding *nido*-derivatives **4a**,**b** which were isolated as internal salts in moderate yields (Scheme 1). Their treatment with sodium carbonate afforded sodium salts of *N*^ω^-carboranyl amino acids **5a** and **5b** in a quantitative yield.

**Scheme 2:**
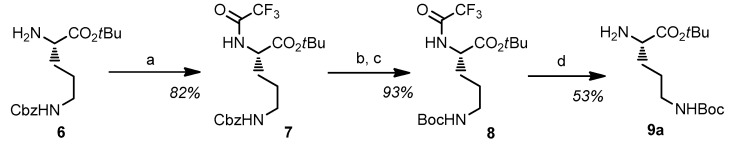
Synthesis of *tert*-butyl *N*^δ^-Boc-(*S*)-ornithinate (**9a**). (a) TFAA, NEt_3_, CH_2_Cl_2_, −5 °C to +10 °C, 24 h; (b) H_2_ (8 atm), 10% Pd/C, EtOH, rt, 8 h. (c) Boc_2_O, NEt_3_, DMF, rt, 16 h. (d) 1 *M* NaOH, EtOH, rt, 6 h.

To obtain *N*^α^-carboranylacetyl derivatives, it was necessary to synthesize suitably protected (*S*)-ornithine and (*S*)-lysine. This synthesis involved more steps. In the case of the ornithine derivative, we carried out the following sequential processes: transformation of amino ester **6** into *N*^δ^-Cbz-*N*^α^-trifluoroacetyl derivative **7**, removal of the Cbz group by hydrogenolysis, and introduction of the Boc group to form compound **8** (Scheme 2). Removal of the trifluoroacetyl protecting group in compound **8** under mild alkaline conditions by analogy with the known approach [94] gave amino ester **9a** in 40% overall yield relative to amino ester **6**. The choice of this synthetic route for compound **9a** with a free amino group in the α-position was due to the instability of readily available *N*^δ^-Boc-(*S*)-ornithine under the esterification conditions and the limited availability of *N*^α^-Cbz-(*S*)-ornithine.

**Scheme 3:**
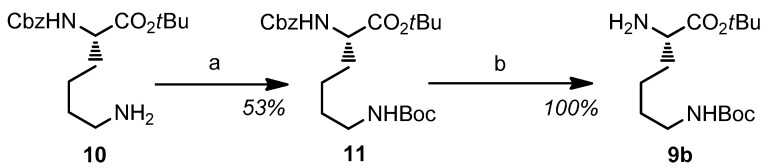
Synthesis of *tert*-butyl *N*^ε^-Boc-(*S*)-lysinate (**9b**). (a) Boc_2_O, NEt_3_, DMF, rt, 24 h. (b) H_2_ (25 atm), 10% Pd/C, EtOH, rt, 6 h.

In contrast, *N*^α^-Cbz-(*S*)-lysine is a commercially available starting material. At first, it was converted to *tert*-butyl amino ester **10** according to the known procedure [95]; further transformation of compound **10** to *N*^ε^-Boc derivative **11** followed by the removal of the Cbz protecting group afforded amino ester **9b** in 53% overall yield relative to compound **10** (Scheme 3). It should be noted that hydrogenation of compound **11** required more drastic reaction conditions than those used for preparing compounds **2a**,**b** and deprotection of compound **7** (hydrogen pressure of 25 atm in the presence of 20% catalyst by weight).

**Scheme 4:**
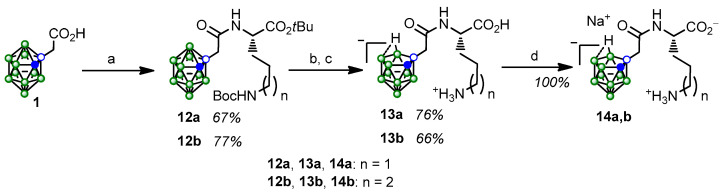
Synthesis of *N*^α^-(*nido*-carboran-7-yl)acetyl derivatives of (*S*)-ornithine and (*S*)-lysine. (a) Compound **9a** (or **9b**), EDC×HCl, HOBt, NEt_3_, CH_2_Cl_2_, rt, 24 h. (b) CsF, EtOH, Δ, 16 h. (c) HCl conc., 1,4-dioxane, rt, 3 h. (d) Na_2_CO_3_, H_2_O/EtOH, rt, 0.5 h.

Acylation of compounds **9a** and **9b** with acid **1** by the carbodiimide method smoothly afforded amides **12a**,**b** (Scheme 4). Successive treatment of compounds **12a**,**b** with CsF and concentrated hydrochloric acid in 1,4-dioxane afforded *nido*-carboranyl amino acids **13a** and **13b** as zwitterions. Similarly to amino acids **4a**,**b**, compounds **13a**,**b** readily formed sodium salts **14a**,**b** upon treatment with an equivalent amount of Na_2_CO_3_.

(*nido*-Carboran-7-yl)acetyl derivatives of (*S*)-ornithine and (*S*)-lysine **4a**,**b** and **13a**,**b**, isolated as zwitterions, are solids without a clear melting point. These compounds are slightly soluble in water and have a high tendency to form solvates with ethyl acetate, the content of which in the samples dried to constant weight reached 50 mol.% (according to elemental analysis and ^1^H NMR spectroscopy). Sodium salts **5a**,**b** and **14a**,**b** are finely crystalline compounds stable upon storage for several months. Their solubility in water is 5–7 mg/mL.

In the molecules of 7-monosubstituted *nido*-carboranes there is a chiral plane. As a result of the treatment of *closo*-carborane derivatives **3a**,**b** and **12a**,**b** with CsF, the deboronation of the B(3)H and B(6)H vertices occurs with equal probability. Therefore, compounds **4**, **5**, **13** and **14** are mixtures of two diastereomers in a ratio of approximately 1:1. In the case of amino acid derivatives substituted at the α-amino group (compounds **13a**,**b** and **14a**,**b**), two chirality elements (the asymmetric carbon atom of the amino acid and the chiral plane in the *nido*-carborane residue) are close to each other, and individual diastereomers are clearly distinguishable in the NMR spectra (see the Supplementary Materials, Figures S35–S46). At the same time, the NMR spectra of the *N*^ε^-derivatives of (*S*)-lysine **4b** and **5b** contain a single set of signals. The signals from bridging hydrogen atoms in *nido*-carborane residues of compounds **4**, **5**, **13** and **14** were in the upper field (*δ* −2.6…−2.8 ppm); the signals of CH groups in *nido*-carborane residues of compounds **4** and **13** were shifted upfield compared to the corresponding signals in the ^1^H NMR spectra of *closo*-derivatives **3** and **12** (*δ* 1.83–1.86 and 5.05–5.12 ppm, respectively). The ^11^B NMR spectra of compounds **4**, **5**, **13** and **14** contained sets of clearly distinguishable signals in the range *δ* −10.4…−37.5 ppm, which is characteristic of non-symmetrical mono-substituted *nido*-carborane derivatives [53,82,96,97]. The mass spectra of *closo*- and *nido*-carborane derivatives showed characteristic cluster peaks, which is associated with the presence of ten or nine boron atoms at different ratios of ^10^B and ^11^B isotopes (see the Supplementary Materials, Figures S47–S59).

### 2.2. In Vitro Evaluation

To test the ability to deliver boron into cells, we chose sodium salts of (*S*)-ornithine and (*S*)-lysine derivatives **5a**,**b** with unsubstituted functional groups at the alpha-position. We assumed that the presence of unmodified NH_2_ and CO_2_H groups at the alpha-position may promote more active transport into cells, for example, with the participation of the large neutral L-type amino acid transporter 1 (LAT1) [15,16,98,99,100].

#### 2.2.1. Toxicity Assay

**Figure 2:**
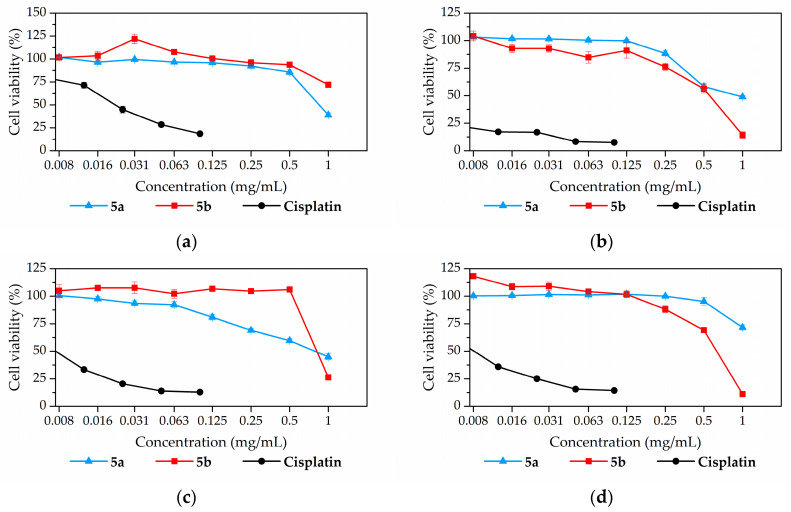
Effect of compounds **5a** and **5b** on the viability of various cell lines in vitro within 72 h of co-incubation: (**a**) BJ-5ta human foreskin fibroblasts, (**b**) DU 145 human prostate carcinoma, (**c**) MDA-MB-231 human breast carcinoma, (**d**) SK-Mel-28 human melanoma.

The toxicity profile of compound **5b** and its homologue **5a** was studied in the MTT assay [101] on healthy (nontransformed) cells (BJ-5ta human foreskin fibroblasts) and tumour cells (DU 145 human prostate carcinoma, MDA-MB-231 human breast carcinoma, SK-Mel-28 human melanoma, T98G and U87 MG human glioblastomas) (Figure 2, Table 1). The antitumour agent cisplatin was used as a positive control (at concentrations 10 times lower than those of compounds **5a**,**b**). Cell viability in negative control samples (without tested compounds in growth medium) was 100 ± 11%.

The cells were incubated in the presence of the test compounds for 72 h. It has been shown that sodium salts **5a**,**b** do not exhibit a significant effect on the survival of BJ-5ta fibroblasts and various human tumour cells at a concentration of 0.125 mg/mL and below (Figure 2). At the same time, the sodium salt of *N*^ε^-(*nido*-carboran-7-yl)acetyl-(*S*)-lysine (**5b**) was less toxic to healthy BJ-5ta cells than to tumour cells (Table 1). The (*S*)-ornithine derivative **5a** was less toxic to tumour cells than compound **5b**; however, it was characterized by greater cytotoxicity towards BJ-5ta fibroblasts.

#### 2.2.2. Evaluation of Boron Accumulation

Evaluation of boron accumulation in tumour and healthy cells is an important step in the design of potential agents for BNCT. Candidate compounds should provide selective boron accumulation in tumour cells accompanied by minimal cytotoxicity.

To test the ability to deliver boron into cells, we chose sodium salt of the (*S*)-lysine derivative **5b** demonstrating lower toxicity towards healthy cells than its homologue **5a**. In addition, it is known that *N*^ε^-acetylated (*S*)-lysine derivatives are good substrates for histone deacetylase that is actively expressed by various tumour cells [24,102,103].

**Figure 3:**
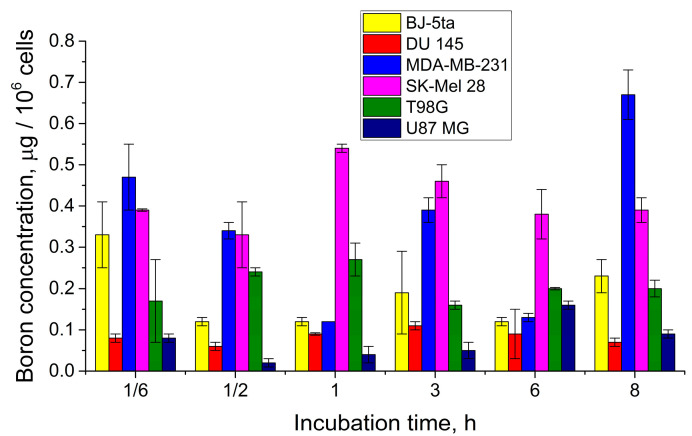
Cellular uptake of compound **5b** by various cells in vitro (*p* = 0.95, *n* = 3).

Incubation of cells of different lines in the presence of (*S*)-lysine derivative **5b** (at a concentration of 0.5 µg/mL) was carried out for 8 h to minimize the cytotoxic effect. The highest level of boron concentration was observed in MDA-MB-231 human breast carcinoma cells (up to 0.67 µg B/10^6^ cells after incubation for 8 h) (Figure 3).

The level of boron accumulation in SK-Mel 28 melanoma cells was slightly lower (up to 0.54 µg B/10^6^ cells after 1 h of incubation). The highest boron concentrations in cells using compound **5b** exceeded the level of boron accumulation in melanoma and breast cancer cells previously achieved using boronophenylalanine (BPA) and sodium borocaptate (BSH), clinically used agents for BNCT [104,105,106]. T98G and U87 MG glioblastoma cells, DU 145 prostate carcinoma cells, and BJ-5ta foreskin fibroblasts were characterized by low levels of compound **5b** accumulation.

It is noteworthy that the previously synthesized *bis*-amide of folic acid containing *nido*-carborane residues was selectively accumulated by U87 MG human glioblastoma cells, while the level of accumulation by MDA-MB-231 and SK-Mel 28 cell lines was low [53]. This indicates differences in the mechanism of transport of conjugates based on folic acid and (*S*)-lysine into cells.

The high demand of tumour cells for nutrients is largely ensured by amino acid transporters, primarily the large neutral L-amino acid transporter 1 (LAT1) and the alanine/serine/cysteine transporter 2 (ASCT2) [61,107,108]. In particular, LAT1 and ASCT2 have broad substrate specificity for amino acids and their derivatives and are actively expressed by human breast cancer cells (including MDA-MB-231) [109,110,111,112,113]. Human melanoma cells typically express high levels of LAT1 and are sensitive to LAT1-targeted therapy [114,115,116,117]. Moreover, it is known that SK-Mel 28 human melanoma cells are characterized by low activity of the ASCT2 transporter [118]. In addition, it is known that melanoma cells are able to accumulate boron-containing amino acids (e.g., ^10^B-BPA) via active transport [119,120,121]. Apparently, a significant level of accumulation of boron-containing amino acid **5b** by MDA-MB-231 and SK-Mel 28 cells is due to LAT1-mediated transport through the cell membrane. In recent years, good prospects for using BNCT for combating triple-negative breast cancers and melanoma using BPA and boron-containing nanoparticles have been demonstrated [122,123,124,125]. Therefore, design of efficient agents for boron delivery to melanoma and breast carcinoma cells is an important step in the development of BNCT.

## 3. Materials and Methods

### 3.1. Chemistry General Section

(1,2-Dicarba-*closo*-dodecaboran-1-yl)acetic acid (**1**) [126], *tert*-butyl *N*^α^-(*tert*-butoxycarbonyl)-(*S*)-ornithinate (**2a**) [83], *tert*-butyl *N*^α^-(*tert*-butoxycarbonyl)-(*S*)-lysinate (**2b**) [84], *tert*-butyl *N*^δ^-benzyloxycarbonyl-(*S*)-ornithinate (**6**) [127] and *tert*-butyl *N*^α^-benzyloxycarbonyl-(*S*)-lysinate (**10**) [95] were obtained as described previously. Other reagents are commercially available. Solvents were purified according to traditional methods [128] and used freshly distilled.

Melting points were obtained on a SMP3 apparatus (Barloworld Scientific, Staffordshire, UK) and are uncorrected. Optical rotations were measured on a Perkin Elmer 341 polarimeter (Perkin Elmer, Waltham, MA, USA). The ^1^H, ^11^B, ^13^C and ^19^F NMR spectra were recorded on a Bruker AVANCE 500 instrument (Bruker, Karlsruhe, Germany) (500, 160, 126 and 470 MHz, respectively) at ambient temperature using TMS and C_6_F_6_ as internal standards and BF_3_·Et_2_O as an external standard. The NMR spectra of the compounds were obtained; see the Supplementary Materials, Figures S1–S46. Microanalyses were carried out using a Perkin Elmer 2400 II automatic analyser (Perkin Elmer, Waltham, MA, USA). Analytical TLC was performed using Sorbfil plates (Imid, Krasnodar, Russia). Flash column chromatography was performed using Silica gel 60 (230–400 mesh) (Alfa Aesar, Heysham, Lancashire, UK). The high-resolution mass spectra were obtained on a Bruker maXis Impact HD mass spectrometer (Bruker, Karlsruhe, Germany), electrospray ionization in positive or negative mode with direct sample inlet (4 L/min flow rate).

Single crystal of compound **3b** was obtained by spontaneous crystallization from MeOH. The XRD experiments were performed on an Xcalibur 3 automated X-ray diffractometer (Oxford Diffraction, Milton Park Abingdon, Oxfordshire, UK) with CCD detector according to standard procedure (Mo*K*α-irradiation, graphite monochromator, ω-scans with 1° step at *T* = 295 (2) K). Empirical absorption correction was applied. The solution and refinement of the structures were accomplished with using OLEX2-1.3 software package [129]. The structures were solved by direct method in SHELXS programme and refined by SHELXL by full-matrix least-squared method for non-hydrogen atoms [130]. The H atoms at B–H and C–H bonds were placed in the calculated positions and refined in the “rider” model; the H atoms at N–H bonds were refined independently in isotropic approximation. The X-ray diffraction data were deposited with the Cambridge Crystallographic Data Centre (CCDC no. 2472575). Copies of the data can be obtained, free of charge, on application to CCDC, 12 Union Road, Cambridge CB2 1EZ, UK [fax: +44(0)1223 336033 or e-mail: deposit@ccdc.cam.ac.uk].

### 3.2. Synthesis

**General Procedure for the Synthesis of *tert*-Butyl *N*^α^-Boc-*N*^ω^-(*closo*-carboranyl)acetylamino Esters 3a,b**. Ethyl chloroformate (0.17 mL, 1.80 mmol) was added to a solution of compound **1** (0.36 g, 1.80 mmol) and *N*-methylmorpholine (0.20 mL, 1.80 mmol) in THF (15 mL) with stirring at −12 °C. The reaction mixture was stirred at −12 °C for 20 min, then a solution of compound **2a** or **2b** (1.80 mmol) and *N*,*N*-diethylaniline (0.54 g, 3.60 mmol) in THF (20 mL) was added. The resulting suspension was stirred at room temperature for 24 h, then evaporated to dryness under reduced pressure, and EtOAc (45 mL) was added to the residue. The resulting solution was washed successively with 10% citric acid solution (3 × 20 mL), saturated NaCl solution (3 × 20 mL), 5% NaHCO_3_ solution (3 × 20 mL), and water (2 × 20 mL). The organic layer was dried with Na_2_SO_4_ and evaporated to dryness under reduced pressure. The residue was purified by flash chromatography on silica gel (eluent hexane–EtOAc from 9:1 to 1:1).

*tert*-Butyl *N*^α^-*tert*-Butoxycarbonyl-*N*^δ^-(1,2-dicarba-*closo*-dodecaboran-1-yl)acetyl-(*S*)-ornithinate (**3a**). Yield 0.52 g (61%), colourless solid, mp. 64–66 °C. [α]_D_^20^ −9.5 (*c* 0.5, EtOH). ^1^H NMR (500 MHz, DMSO-*d*_6_) (conformers I and II, 85:15) δ (ppm): 1.3–2.8 (m, 10H, 10 × BH), 1.36 (s, 1.35H, *t*Bu (II)), 1.38 (s, 7.65H, *t*Bu (I)), 1.39 (s, 7.65H, *t*Bu (I)), 1.42 (s, 1.35H, *t*Bu (II)), 1.39–1.54 (m, 3H, 2 × H-4, H-3^B^), 1.60–1.67 (m, 1H, H-3^A^), 2.96–3.06 (m, 2H, 2 × H-5), 3.09 (s, 2H, CH_2_ acetyl), 3.65–3.70 (m, 0.15H, H-2 (II)), 3.77 (br. td, *J* = 8.3, 5.0 Hz, 0.85H, H-2 (I)), 5.12 (s, 1H, CH carborane), 6.77 (d, *J* = 7.1 Hz, 0.15H, NH-2 (II)), 7.10 (d, *J* = 7.8 Hz, 0.85H, NH-2 (I)), 8.22 (t, *J* = 5.6 Hz, 1H, NH-5). ^11^B{^1^H} NMR (160 MHz, DMSO-*d*_6_) δ (ppm): −13.1 (2B), −11.0 (3B), −10.1 (3B), −6.0 (1B), −3.2 (1B). ^13^C NMR (126 MHz, DMSO-*d*_6_) (predominant conformer) δ (ppm): 25.31, 27.56 (3C), 28.11 (4C), 38.26, 42.24, 53.98, 61.19, 71.41, 77.96, 80.15, 155.44, 165.39, 171.61. HRMS (ESI): *m*/*z* [M − H]^−^ calcd for [C_18_H_39_^11^B_10_N_2_O_5_]^−^: 473.3824, found: 473.3823.

*tert*-Butyl *N*^α^-*tert*-Butoxycarbonyl-*N*^ε^-(1,2-dicarba-*closo*-dodecaboran-1-yl)acetyl-(*S*)-lysinate (**3b**). Yield 0.60 g (68%), colourless solid, mp. 155–156 °C. [α]_D_^20^ −14.6 (*c* 0.9, MeOH). ^1^H NMR (500 MHz, DMSO-*d*_6_) (conformers I and II, 85:15) δ (ppm): 1.3–2.9 (m, 10H, 10 × BH), 1.24–1.41 (m, 4H, 2 × H-4, 2 × H-5), 1.35 (s, 1.35H, *t*Bu (II)), 1.38 (s, 7.65H, *t*Bu (I)), 1.39 (s, 7.65H, *t*Bu (I)), 1.41 (s, 1.35H, *t*Bu (II)), 1.49–1.62 (m, 2H, 2 × H-3), 2.96–3.07 (m, 2H, 2 × H-6), 3.09 (s, 2H, CH_2_ acetyl), 3.63–3.68 (m, 0.15H, H-2 (II)), 3.73 (ddd, *J* = 9.0, 7.8, 5.1 Hz, 0.85H, H-2 (I)), 5.12 (s, 1H, CH carborane), 6.73 (d, *J* = 7.4 Hz, 0.15H, NH-2 (II)), 7.05 (d, *J* = 7.8 Hz, 0.85H, NH-2 (I)), 8.21 (t, *J* = 5.6 Hz, 1H, NH-5). ^11^B{^1^H} NMR (160 MHz, DMSO-*d*_6_) δ (ppm): −13.0 (2B), −11.0 (3B), −10.1 (3B), −6.0 (1B), −3.2 (1B). ^13^C NMR (126 MHz, DMSO-*d*_6_) (predominant conformer) δ (ppm): 22.89, 27.59 (3C), 28.12 (3C), 28.19, 30.21, 38.39, 42.21, 54.25, 61.16, 71.44, 77.92, 80.07, 155.47, 165.36, 171.78. Calcd (%) for C_19_H_42_B_10_N_2_O_5_ (486.65): C 46.89, H 8.70, N 5.76. Found (%): C 47.15, H 8.64, N 5.71.

**General Procedure for the Synthesis of *N*^ω^-(*nido*-Carboranyl)acetylamino acids 4a,b**. CsF (0.39 g, 2.55 mmol) was added to a solution of compound **3a** or **3b** (0.85 mmol) in EtOH (25 mL). The reaction mixture was refluxed for 16 h, then cooled to room temperature and evaporated to dryness under reduced pressure. The residue was dissolved in water (50 mL) and extracted with EtOAc (3 × 15 mL). The organic layers were washed with saturated NaCl solution (2 × 20 mL), dried over Na_2_SO_4_, and evaporated to dryness under reduced pressure. The residue was dissolved in dioxane (12 mL), then conc. HCl (2.1 mL, 25.5 mmol) was added. The mixture was stirred at room temperature for 3 h, then evaporated to dryness under reduced pressure. The residue was dissolved in water (15 mL) and extracted with EtOAc (3 × 10 mL). The organic layers were washed with water (3 × 10 mL), dried over Na_2_SO_4_, and evaporated to dryness under reduced pressure. The residue was purified by flash chromatography (eluent CHCl_3_–EtOH–NH_4_OH from 8:2:0.5 to 4:6:0.5). Fractions containing the target compound were evaporated to dryness under reduced pressure. The residue was dissolved in 0.2 M HCl (20 mL), extracted with EtOAc (3 × 10 mL). The organic layers were washed with saturated NaCl solution (2 × 10 mL), dried over Na_2_SO_4_, and evaporated to dryness under reduced pressure.

*N*^δ^-(7,8-Dicarba-*nido*-undecaboran-7-yl)acetyl-(*S*)-ornithine (**4a**). Yield 0.13 g (50%), colourless solid, mp. 91–93 °C. [α]_D_^20^ +9.4 (*c* 0.5, EtOH). ^1^H NMR (500 MHz, DMSO-*d*_6_) (diastereomers I and II, appr. 1:1) δ (ppm): −2.73 (br. s, 0.5H, BHB (I)), −2.63 (br. s, 0.5H, BHB (II)), −0.6 … 2.3 (br. m, 9H, 9 × BH), 1.40–1.58 (m, 2H, 2 × H-3), 1.68–1.82 (m, 2H, 2 × H-4), 1.85 (s, 1H, CH carborane), 2.050 (d, *J* = 14.7 Hz, 0.5H, H^B^ acetyl (I)), 2.054 (d, *J* = 14.7 Hz, 0.5H, H^B^ acetyl (II)), 2.40 (d, *J* = 14.7 Hz, 1H, H^A^ acetyl (I and II)), 3.04 (q, *J* = 6.6 Hz, 2H, 2 × H-5), 3.92 (t, *J* = 6.0 Hz, 1H, H-2), 7.46 (t, *J* = 5.3 Hz, 1H, NH), 8.18 (s, 3H, NH_3_^+^), 13.80 (br.s, 1H, CO_2_H). ^11^B NMR (160 MHz, DMSO-*d*_6_) δ (ppm): −37.1 (d, *J* = 134 Hz, 1B), −33.3 (d, *J* = 125 Hz, 1B), −22.0 (d, *J* = 110 Hz, 1B), −18.8 … −16.9 (m, 3B), −14.3 (d, *J* = 120 Hz, 1B), −10.8 (d, *J* = 130 Hz, 2B). ^13^C NMR (126 MHz, DMSO-*d*_6_) δ (ppm): 24.92, 27.56, 37.79, 45.36, 46.30 (br. s), 51.81, 54.72 (br. s), 170.98 (2C). HRMS (ESI): *m*/*z* [M − H]^−^ calcd for [C_9_H_24_^11^B_9_N_2_O_3_]^−^: 306.2645, found: 306.2643.

*N*^ε^-(7,8-Dicarba-*nido*-undecaboran-7-yl)acetyl-(*S*)-lysine, Solvate with EtOAc (**4b**). Yield 0.18 g (58%), colourless semisolid. [α]_D_^20^ +5.9 (*c* 0.9, MeOH). ^1^H NMR (500 MHz, DMSO-*d*_6_) δ (ppm): −2.72 (br. s, 1H, BHB), −0.6…2.4 (br. m, 9H, 9 × BH), 1.30–1.46 (m, 4H, 2 × CH_2_), 1.74–1.83 (m, 2H, 2 × H-3), 1.86 (br.s, 1H, CH carborane), 2.03 (d, *J* = 14.5 Hz, 1H, H^B^ acetyl), 2.38 (d, *J* = 14.5 Hz, 1H, H^A^ acetyl), 2.97–3.06 (m, 2H, 2 × H-6), 3.88 (br. s, 1H, H-2), 7.34 (t, *J* = 5.3 Hz, 1H, NH), 8.17 (s, 3H, NH_3_^+^), 13.80 (s, 1H, CO_2_H). ^11^B{^1^H} NMR (160 MHz, DMSO-*d*_6_) δ (ppm): −37.1 (1B), −33.3 (1B), −22.0 (br. s, 1B), −18.4 (2B), −17.2 (1B), −14.2 (br. s, 1B), −10.8 (2B). ^13^C NMR (126 MHz, DMSO-*d*_6_) δ (ppm): 21.69, 28.67, 29.61, 37.88, 45.41, 46.20 (br. s), 51.85, 54.93 (br. s), 170.79, 171.02. Calcd (%) for C_10_H_27_B_9_N_2_O_3_ × 0.5EtOAc (364.68): C 39.52, H 8.57, N 7.68. Found (%): C 39.74, H 8.35, N 7.38.

**General Procedure for the Synthesis of Sodium Salts of *N*^ω^-(*nido*-Carboranyl)acetyl-amino Acids 5a,b.** A solution of Na_2_CO_3_ (0.016 g, 0.15 mmol) in water (1 mL) was added to a solution of compound **4a** or **4b** (0.30 mmol) in EtOH (5 mL). The reaction mixture was evaporated to dryness under reduced pressure. The residue was dried in vacuo over P_2_O_5_ at 70 °C.

Sodium *N*^δ^-(7,8-Dicarba-*nido*-undecaboran-7-yl)acetyl-(*S*)-ornithinate (**5a**). Yield 0.098 g (100%). Colourless semisolid. [α]_D_^20^ −3.1 (*c* 0.3, H_2_O). ^1^H NMR (500 MHz, D_2_O) δ (ppm): −3.05 … −2.35 (br. s, 1H, BHB), −0.4…2.5 (m, 9H, BH), 1.56–1.71 (m, 2H, 2 × H-3), 1.88–2.01 (m, 2H, 2 × H-4), 2.09 (s, 1H, CH carborane), 2.37 (d, *J* = 14.6 Hz, 1H, H^B^ acetyl), 2.57 (d, *J* = 14.6 Hz, 1H, H^A^ acetyl), 3.19–3.31 (m, 2H, 2 × H-5), 3.83 (t, *J* = 6.2 Hz, 1H, H-2). ^11^B NMR (160 MHz, D_2_O) δ (ppm): −37.4 (d, *J* = 138 Hz, 1B), −33.4 (d, *J* = 130 Hz, 1B), −21.4 (d, *J* = 146 Hz, 1B), −19.6 (d, *J* = 140 Hz, 1B), −18.7 (d, *J* = 160 Hz, 1B), −17.0 (d, *J* = 135 Hz, 1B), −14.2 (d, *J* = 154 Hz, 1B), −11.8 (d, *J* = 114 Hz, 1B), −11.1 (d, *J* = 115 Hz, 1B). ^13^C NMR (126 MHz, D_2_O) δ (ppm): 27.16, 30.58, 41.29, 47.78, 49.92 (br. s), 56.86, 59.05 (br. s), 176.64, 177.75. HRMS (ESI): *m*/*z* [M − Na]^−^ calcd for [C_9_H_24_^11^B_9_N_2_O_3_]^−^: 306.2645, found: 306.2644.

Sodium *N*^ε^-(7,8-Dicarba-*nido*-undecaboran-7-yl)acetyl-(*S*)-lysinate (**5b**). Yield 0.103 g (100%). Colourless semisolid. [α]_D_^20^ +2.4 (*c* 0.5, MeOH). ^1^H NMR (500 MHz, D_2_O) δ (ppm): −3.05 … −2.35 (br. s, 1H, BHB), −0.4 … 2.5 (m, 9H, BH), 1.42–1.53 (m, 2H, 2 × H-4), 1.57–1.62 (m, 2H, 2 × H-5), 1.82–1.97 (m, 2H, 2 × H-3), 2.09 (s, 1H, CH carborane), 2.35 (d, *J* = 14.5 Hz, 1H, H^B^ acetyl), 2.56 (d, *J* = 14.5 Hz, 1H, H^A^ acetyl), 3.16–3.27 (m, 2H, 2 × H-6), 3.75 (t, *J* = 6.0 Hz, 1H, H-2). ^11^B{^1^H} NMR (160 MHz, D_2_O) δ (ppm): −37.4 (1B), −33.4 (1B), −21.4 (1B), −19.6 (1B), −18.6 (m, 1B), −17.0 (1B), −14.2 (1B), −11.7 (1B), −11.2 (1B). ^13^C NMR (126 MHz, D_2_O) δ (ppm): 24.75, 30.93, 32.87, 41.48, 47.83, 49.87 (br. s), 57.53, 59.27 (br. s), 177.44, 177.62. HRMS (ESI): *m*/*z* [M + H]^+^ calcd for [C_10_H_27_^11^B_9_N_2_NaO_3_]^+^: 345.2768, found: 345.2764; [M + Na]^+^ calcd for [C_10_H_26_^11^B_9_N_2_Na_2_O_3_]^+^: 367.2587, found: 367.2584.

*tert*-Butyl *N*^δ^-Benzyloxycarbonyl-*N*^α^-trifluoroacetyl-(*S*)-ornithinate (**7**). Trifluoroacetic anhydride (0.63 mL, 4.50 mmol) was added dropwise to a solution of compound **6** (1.32 g, 4.09 mmol) and triethylamine (1.83 mL, 13.10 mmol) in CH_2_Cl_2_ (11 mL) with stirring at −5 °C. The reaction mixture was stirred at −5 °C for 10 min, then at 10 °C for 24 h, CH_2_Cl_2_ (10 mL) was added and the mixture was washed successively with 10% citric acid solution (3 × 10 mL), saturated NaCl solution (2 × 10 mL), 5% NaHCO_3_ solution (2 × 10 mL) and water (10 mL). The organic layer was dried over MgSO_4_ and evaporated to dryness under reduced pressure. The residue was purified by flash column chromatography on silica gel (eluent benzene–EtOAc from 95:5 to 85:15). Yield 1.40 g (82%). Colourless solid, mp. 68–70 °C. [α]_D_^20^ −23.4 (*c* 1.3, MeOH). ^1^H NMR (500 MHz, DMSO-*d*_6_) δ (ppm): 1.40 (s, 9H, *t*Bu), 1.42–1.49 (m, 2H, 2 × H-4), 1.65–1.74 (m, 1H, H-3^B^), 1.76–1.84 (m, 1H, H-3^A^), 2.98–3.02 (m, 2H, 2 × H-5), 4.16 (ddd, *J* = 9.4, 7.3, 5.1 Hz, 1H, H-2), 5.00 (s, 2H, CH_2_,Bn), 7.28 (t, *J* = 5.9 Hz, 1H, NH-5), 7.30–7.38 (m, 5H, Bn), 9.70 (d, *J* = 7.3 Hz, 1H, NH-2). ^19^F NMR (470 MHz, DMSO-*d*_6_) δ (ppm): 88.45 (s, CF_3_). ^13^C NMR (126 MHz, DMSO-*d*_6_) δ (ppm): 25.93, 27.10, 27.45 (3C), 39.5 (overlapped with DMSO-*d*_6_ signal), 53.16, 65.07, 81.18, 115.74 (q, *J* = 287.9 Hz), 127.62 (2C), 127.66, 128.25 (2C), 137.18, 156.04, 156.47 (q, *J* = 36.9 Hz), 169.39. HRMS (ESI): *m*/*z* [M + H]^+^ calcd for [C_19_H_26_F_3_N_2_O_5_]^+^: 419.1788, found: 419.1787.

*tert*-Butyl *N*^δ^-*tert*-Butoxycarbonyl-*N*^α^-trifluoroacetyl-(*S*)-ornithinate (**8**). 10% Pd/C (0.15 g) was added to a solution of compound **7** (1.03 g, 2.45 mmol) in EtOH (40 mL). The reaction mixture was stirred at room temperature under H_2_ (8 atm.) for 8 h. The solution was filtered; the filtrate was evaporated to dryness under reduced pressure. The residue was dissolved in DMF (5 mL); triethylamine (0.68 mL, 4.90 mmol) and a solution of Boc_2_O (0.59 g, 2.70 mmol) in DMF (6.5 mL) were added. The reaction mixture was stirred at room temperature for 16 h, then EtOAc (25 mL) was added. The solution was washed successively with 10% citric acid solution (3 × 10 mL) and saturated NaCl solution (2 × 10 mL). The organic layer was dried with MgSO_4_ and evaporated to dryness under reduced pressure. The residue was purified by flash column chromatography on silica gel (eluent benzene–EtOAc from 95:5 to 9:1). Yield 0.88 g (93%). Colourless solid, mp. 97–98 °C. [α]_D_^20^ –27 (*c* 0.8, MeOH). ^1^H NMR (500 MHz, DMSO-*d*_6_) (predominant conformer) δ (ppm): 1.37 (s, 9H, *t*Bu), 1.40 (s, 9H, *t*Bu), 1.38–1.44 (m, 2H, 2 × H-4), 1.63–1.71 (m, 1H, H-3^B^), 1.73–1.82 (m, 1H, H-3^A^), 2.90–2.93 (m, 2H, 2 × H-5), 4.13–4.18 (m, 1H, H-2), 6.81 (t, *J* = 5.7 Hz, 1H, NH-5), 9.68 (br. d, *J* = 5.0 Hz, 1H, NH-2). ^19^F NMR (470 MHz, DMSO-*d*_6_) (predominant conformer) δ (ppm): 88.45 (s, CF_3_). ^13^C NMR (126 MHz, DMSO-*d*_6_) (predominant conformer) δ (ppm): 26.02, 27.08, 27.45 (3C), 28.16 (3C), 39.05, 53.16, 77.35, 81.14, 115.73 (q, *J* = 288.0 Hz), 155.55, 156.45 (q, *J* = 36.6 Hz), 169.44. HRMS (ESI): *m*/*z* [M + Na]^+^ calcd for [C_16_H_27_F_3_N_2_NaO_5_]^+^: 407.1764, found: 407.1762.

*tert*-Butyl *N*^δ^-*tert*-Butoxycarbonyl-(*S*)-ornithinate (**9a**). 1 *M* NaOH (2.8 mL, 2.80 mmol) was added dropwise to a solution of compound **8** (0.43 g, 1.13 mmol) in EtOH (2.8 mL). The reaction mixture was stirred at room temperature for 6 h, then water (15 mL) was added and extracted with EtOAc (3 × 10 mL). The organic layers were washed with saturated NaCl solution (2 × 10 mL), dried over Na_2_SO_4_, and evaporated to dryness under reduced pressure. Yield 0.17 g (53%). Colourless oil. ^1^H NMR (500 MHz, CDCl_3_) (predominant conformer) δ (ppm): 1.44 (s, 9H, *t*Bu), 1.46 (s, 9H, *t*Bu), 1.50–1.60 (m, 3H, 2 × H-4 and H-3^B^), 1.61 (s, 2H, NH_2_), 1.69–1.76 (m, 1H, H-3^A^), 3.09–3.19 (m, 2H, 2 × H-5), 3.32 (dd, *J* = 6.9, 5.3 Hz, 1H, H-2), 4.73 (br. s, 1H, NH-5). ^13^C NMR (126 MHz, CDCl_3_) (predominant conformer) δ (ppm): 26.33, 28.05 (3C), 28.41 (3C), 32.16, 40.31, 54.71, 79.06, 81.05, 155.96, 175.21. HRMS (ESI): *m*/*z* [M + H]^+^ calcd for [C_14_H_29_N_2_O_4_]^+^: 289.2122, found: 289.2121.

*tert*-Butyl *N*^α^-Benzyloxycarbonyl-*N*^ε^-*tert*-butoxycarbonyl-(*S*)-lysinate (**11**). Boc_2_O (0.26 g, 1.18 mmol) was added to a solution of compound **10** (0.36 g, 1.07 mmol) and triethylamine (0.30 mL, 2.14 mmol) in DMF (5 mL) with stirring. The reaction mixture was stirred at room temperature for 24 h, then EtOAc (20 mL) was added. The solution was washed successively with 10% citric acid solution (3 × 15 mL) and saturated NaCl solution (2 × 15 mL), dried over Na_2_SO_4_, and evaporated to dryness under reduced pressure. The residue was purified by flash column chromatography on silica gel (eluent hexane–EtOAc from 9:1 to 8:2). Yield 0.25 g (53%). Colourless oil. [α]_D_^20^ −14.3 (*c* 0.9, MeOH). ^1^H NMR (500 MHz, DMSO-*d*_6_) (predominant conformer) δ (ppm): 1.23–1.38 (m, 4H, 2 × H-4 and 2 × H-5), 1.36 (s, 9H, *t*Bu), 1.38 (s, 9H, *t*Bu), 1.49–1.66 (m, 2H, 2 × H-3), 2.84–2.91 (m, 2H, 2 × H-6), 3.81–3.85 (m, 1H, H-2), 5.01 (d, *J* = 12.5 Hz, 1H, H^B^-Bn), 5.05 (d, *J* = 12.5 Hz, 1H, H^A^-Bn), 6.75 (t, *J* = 5.8 Hz, 1H, NH-6), 7.29–7.38 (m, 5H, Bn), 7.55 (d, *J* = 7.7 Hz, 1H, NH-2). ^13^C NMR (126 MHz, DMSO-*d*_6_) (predominant conformer) δ (ppm): 22.64, 27.56 (3C), 28.19 (3C), 28.91, 30.40, 39.3 (overlapped with DMSO-*d*_6_), 54.49, 65.29, 77.25, 80.31, 127.64 (2C), 127.71, 128.23 (2C), 136.96, 155.49, 156.01, 171.53. Calcd (%) for C_23_H_26_N_2_O_6_ (436.55): C 63.28, H 8.31, N 6.42. Found (%): C 63.39, H 8.29, N 6.71.

*tert*-Butyl *N*^ε^-*tert*-Butoxycarbonyl-(*S*)-lysinate (**9b**). 10% Pd/C (0.04 g) was added to a solution of compound 11 (0.20 g, 0.46 mmol) in EtOH (7 mL). The reaction mixture was stirred at room temperature under H_2_ (25 atm.) for 6 h. The solution was filtered; the filtrate was evaporated to dryness under reduced pressure. Yield 0.14 g (100%). Colourless oil. NMR spectra were identical to the published ones [131]. HRMS (ESI): *m*/*z* [M + H]^+^ calcd for [C_15_H_31_N_2_O_4_]^+^: 303.2278, found: 303.2280.

**General Procedure for the Synthesis of *tert*-Butyl *N*^ω^-Boc-*N*^α^-(*closo*-carboranyl)acetylamino Esters 12a,b.** EDC×HCl (0.14 g, 0.72 mmol) was added to a solution of compound **1** (0.12 g, 0.60 mmol), amino ester **9a** or **9b** (0.60 mmol), HOBt hydrate (0.09 g, 0.60 mmol), and triethylamine (0.18 mL, 1.32 mmol) in CH_2_Cl_2_ (5 mL). The reaction mixture was stirred at room temperature for 24 h, then CH_2_Cl_2_ (15 mL) was added. The solution was successively washed with 10% citric acid solution (3 × 10 mL), saturated NaCl solution (3 × 10 mL), 5% NaHCO_3_ solution (2 × 10 mL), and water (10 mL). The organic layer was dried over MgSO_4_ and evaporated to dryness under reduced pressure. The residue was purified by flash column chromatography on silica gel (eluent hexane–EtOAc from 8:2 to 7:3).

*tert*-Butyl *N*^δ^-*tert*-Butoxycarbonyl-*N*^α^-(1,2-dicarba-*closo*-dodecaboran-1-yl)acetyl-(*S*)-ornithinate (**12a**). Yield 0.19 g (67%). Colourless solid, mp. 109–114 °C. [α]_D_^20^ –15.7 (*c* 0.5, EtOH). ^1^H NMR (500 MHz, DMSO-*d*_6_) δ (ppm): 1.3–2.9 (m, 10H, 10 × BH), 1.35–1.43 (m, 20H, 2 × *t*Bu and 2 × H-4), 1.48–1.56 (m, 1H, H-3^B^), 1.60–1.67 (m, 1H, H-3^A^), 2.91 (q, *J* = 6.4 Hz, 2H, 2 × H-5), 3.19 (s, 2H, CH_2_ acetyl), 4.04–4.08 (m, 1H, H-2), 5.05 (s, 1H, CH carborane), 6.83 (t, *J* = 5.6 Hz, 1H, NH-5), 8.54 (d, *J* = 7.4 Hz, 1H, NH-2). ^11^B{^1^H} NMR (160 MHz, DMSO-*d*_6_) δ (ppm): −14.5 … −10.5 (br. m, 5B), −10.0 (3B), −6.0 (1B), −3.1 (1B). ^13^C NMR (126 MHz, DMSO-*d*_6_) δ (ppm): 25.81, 27.50 (3C), 28.17 (3C), 28.26, 39.5 (overlapped by DMSO), 41.70, 52.64, 61.18, 71.23, 77.33, 80.73, 155.53, 165.53, 170.61. Calcd (%) for C_18_H_40_B_10_N_2_O_5_ (472.63): C 45.74, H 8.53, N 5.93. Found (%): C 45.78, H 8.46, N 5.73.

*tert*-Butyl *N*^ε^-*tert*-Butoxycarbonyl-*N*α-(1,2-dicarba-*closo*-dodecaboran-1-yl)acetyl-(*S*)-lysinate (**12b**). Yield 0.22 g (77%). Colourless hygroscopic solid, mp. 55–58 °C. [α]_D_^20^ −18.0 (*c* 0.6, EtOH). ^1^H NMR (500 MHz, DMSO-*d*_6_) (predominant conformer) δ (ppm): 1.0–2.9 (br. m. 10H, 10 × BH), 1.24–1.28 (m, 2H, 2 × H-4), 1.33–1.43 (m, 2H, 2 × H-5), 1.37 (s, 9H, *t*Bu), 1.40 (s, 9H, *t*Bu), 1.50–1.57 (m, 1H, H-3^B^), 1.60–1.67 (m, 1H, H-3^A^), 2.88 (td, *J* = 7.2, 6.7 Hz 2H, 2 × H-6), 3.19 (s, 2H, acetyl), 4.04 (td, *J* = 7.8, 5.8 Hz, 1H, H-2), 5.05 (s, 1H, CH carborane), 6.75 (t, *J* = 5.6 Hz, 1H, NH-6), 8.52 (d, *J* = 7.5 Hz, 1H, NH-2). ^11^B{^1^H} NMR (160 MHz, DMSO-*d*_6_) δ (ppm): −13.0 (br. s, 2B), −10.9 (br. s, 3B), −10.0 (3B), −5.9 (1B), −3.1 (1B). ^13^C NMR (126 MHz, DMSO-*d*_6_) δ (ppm): 22.37, 27.51 (3C), 28.18 (3C), 28.89, 30.55, 39.5 (overlapped with DMSO-*d*_6_), 41.71, 52.74, 61.19, 71.24, 77.25, 80.71, 155.48, 165.53, 170.68. HRMS (ESI): *m*/*z* [M + H]^+^ calcd for [C_19_H_43_^11^B_10_N_2_O_5_]^+^: 489.4119, found: 489.4127.

**General Procedure for the Synthesis of *N*^α^-(*nido*-Carboranyl)acetylamino Acids 13a,b.** CsF (0.16 g, 1.08 mmol) was added to a solution of compound **12a** or **12b** (0.36 mmol) in EtOH (10 mL). The reaction mixture was refluxed for 16 h, then cooled to room temperature and evaporated to dryness under reduced pressure. The residue was dissolved in water (15 mL) and extracted with EtOAc (3 × 10 mL). The organic layers were washed with saturated NaCl solution (2 × 10 mL), dried over Na_2_SO_4_, and evaporated to dryness under reduced pressure. The residue was dissolved in dioxane (5.0 mL) and conc. HCl (1.8 mL, 21.6 mmol) was added. The reaction mixture was stirred at room temperature for 3 h, then evaporated to dryness under reduced pressure. The residue was dissolved in water (10 mL) and extracted with EtOAc (3 × 8 mL). The organic layers were washed with water (3 × 10 mL), dried with Na_2_SO_4_, and evaporated to dryness under reduced pressure. The residue was purified by flash chromatography (eluent CHCl_3_–EtOH–NH_4_OH 2.5:7:0.5). The fractions containing the target compound were evaporated to dryness under reduced pressure. The residue was dissolved in 0.2 M HCl (10 mL), the solution was extracted with EtOAc (3 × 8 mL). The organic layer was washed with saturated NaCl solution (2 × 10 mL), dried with Na_2_SO_4_, and evaporated to dryness under reduced pressure. The residue was dried *in vacuo* over P_2_O_5_ at 80 °C.

*N*^α^-(7,8-Dicarba-*nido*-undecaboran-7-yl)acetyl-(*S*)-ornithine, Solvate with EtOAc (**13a**). Yield 0.09 g (76%). Colourless semisolid. Contains 25 mol% EtOAc according to ^1^H NMR. [α]_D_^20^ −11.7 (*c* 0.4, 1 M HCl). ^1^H NMR (500 MHz, DMSO-*d*_6_) (diastereomers I and II, appr. 1:1) δ (ppm): −2.71 (br. s, 0.5H, BHB (I)), −2.61 (br. s, 0.5H, BHB (II)), −0.5…2.4 (br. m, 9H, 9 × BH), 1.56–1.66 (m, 3H, 2 × H-4 and H-3^B^), 1.77–1.85 (m, 2H, H-3^A^ and CH carborane), 2.10 (d, *J* = 15.0 Hz, 0.5H, H^B^ acetyl (I)), 2.15 (d, *J* = 15.2 Hz, 0.5H, H^B^ acetyl (II)), 2.46–2.50 (m, 1H, H^A^ acetyl (I and II)), 2.78–2.82 (m, 2H, 2 × H-5), 4.21–4.25 (m, 1H, H-2), 7.5–7.8 (m, 4H, NH, NH_3_^+^), 12.65 (br. s, 1H, CO_2_H). ^11^B{^1^H} NMR (160 MHz, DMSO-*d*_6_) δ (ppm): −37.1 (1B), −33.2 (1B), −21.8 (1B), −18.6 (1B), −16.7 (1B), −14.1 (1B), −10.6 (2B). ^13^C NMR (126 MHz, DMSO-*d*_6_) (diastereomers I and II) δ (ppm): 23.58 (I), 23.64 (II), 28.29 (I), 28.36 (II), 38.54 (I and II), 45.12 (I), 45.19 (II), 46.08 (br. s, I and II), 51.06 (I and II), 54.72 (br. s, I and II), 170.99 (I), 171.11 (II), 173.26 (I), 173.32 (II). HRMS (ESI): *m*/*z* [M + H]^+^ calcd for [C_9_H_26_^11^B_9_N_2_O_3_]^+^: 309.2790, found: 309.2787.

*N*^α^-(7,8-Dicarba-*nido*-undecaboran-7-yl)acetyl-(*S*)-lysine, Solvate with EtOAc (**13b**). Yield 0.08 g (66%). Colourless semisolid. Contains 33 mol% EtOAc according to ^1^H NMR. [α]_D_^20^ −11.6 (*c* 0.4, 1 M HCl). ^1^H NMR (500 MHz, DMSO-*d*_6_) (diastereomers I and II, appr. 1:1) δ (ppm): −3.0 … −2.4 (br. s, 1H, BHB, I and II), −0.5…2.4 (br. m, 9H, 9 × BH), 1.36–1.46 (m, 2H, 2 × H-4), 1.51–1.63 (m, 3H, 2 × H-5 and H-3^B^), 1.69–1.78 (m, 1H, H-3^A^), 1.83 (s, 0.5H, CH carborane (I)), 1.86 (s, 0.5H, CH carborane (II)), 2.06 (d, *J* = 14.7 Hz, 0.5H, H^B^ acetyl (I)), 2.16 (d, *J* = 14.8 Hz, 0.5H, H^B^ acetyl (II)), 2.42 (d, *J* = 14.8 Hz, 0.5H, H^A^ acetyl (II)), 2.50 (m, 0.5H, H^A^ acetyl (I), overlapped with DMSO-*d*_6_), 2.79 (t, *J* = 7.6 Hz, 2H, 2 × H-6), 4.18–4.25 (m, 1H, H-2), 7.53 (d, *J* = 8.1 Hz, 0.5H, NH (I)), 7.57 (d, *J* = 8.1 Hz, 0.5H, NH (II)), 7.63 (br. s, 3H, NH_3_^+^), 12.54 (s, 1H, CO_2_H). ^11^B{^1^H} NMR (160 MHz, DMSO-*d*_6_) (diastereomers I and II, appr. 1:1) δ (ppm): −37.0 (1B), −33.2 (1B), −21.8 (1B), −19.0 (0.5B (I)), −18.3 (0.5B (II)), −17.1 (0.5B (I)), −16.3 (0.5B (II)), −14.1 (1B), −10.7 (2B). ^13^C NMR (126 MHz, DMSO-*d*_6_) (diastereomers I and II) δ (ppm): 22.13 (I and II), 26.52 (I and II), 30.78 (I), 30.81 (II), 38.70 (I and II), 45.21 (I), 45.29 (II), 45.57 (br. s, I), 46.00 (br. s, II), 51.07 (I), 51.15 (II), 54.68 (br. s, I), 55.42 (br. s, II), 170.90 (I and II), 173.46 (I and II). HRMS (ESI): *m*/*z* [M + H]^+^ calcd for [C_10_H_28_^11^B_9_N_2_O_3_]^+^: 323.2948, found: 323.2947.

**General Procedure for the Synthesis of Sodium salts of *N*^α^-(*closo*-Carboranyl)acetylamino Acids 14a,b**. A solution of Na_2_CO_3_ (9.7 mg, 0.09 mmol) in water (1 mL) was added to a solution of compound **13a** or **13b** (0.18 mmol) in EtOH (5 mL) with stirring at room temperature. The reaction mixture was evaporated to dryness under reduced pressure. The residue was dried in vacuo over P_2_O_5_ at 70 °C.

Sodium *N*^α^-(1,2-Dicarba-*nido*-dodecaboran-1-yl)acetyl-(*S*)-ornithinate (**14a**). Yield 0.06 g (100%). Colourless solid, mp. 208–212 °C. [α]_D_^20^ −6.8 (*c* 0.4, 1 M NaOH). ^1^H NMR (500 MHz, D_2_O) (diastereomers I and II, appr. 1:1) δ (ppm): −2.76 (br. s, 0.5H, BHB (I)), −2.64 (br. s, 0.5H, BHB (II)), −0.5…2.7 (br. m, 9H, 9 × BH), 1.72–1.85 (m, 3H, 2 × H-4 and H-3^B^), 1.89–1.94 (m, 1H, H-3^A^), 2.09 (s, 1H, CH carborane), 2.34 (d, *J* = 15.1 Hz, 0.5H, H^B^ acetyl (I)), 2.50 (d, *J* = 15.4 Hz, 0.5H, H^B^ acetyl (II)), 2.60 (d, *J* = 15.4 Hz, 0.5H, H^A^ acetyl (II)), 2.70 (d, *J* = 15.1 Hz, 0.5H, H^A^ acetyl (I)), 3.03–3.11 (m, 2H, 2 × H-5), 4.22–4.26 (m, 1H, H-2). ^11^B{^1^H} NMR (160 MHz, D_2_O) δ (ppm): −37.3 (1B), −33.2 (1B), −20.7 (br. s, 2B), −19.0 (1B), −15.9 (1B), −14.0 (1B), −12.0 (1B), −10.7 (1B). ^13^C NMR (126 MHz, D_2_O) (diastereomers I and II) δ (ppm): 25.90 (I), 26.00 (II), 31.56 (I), 31.60 (II), 41.90 (I and II), 47.95 (I), 48.02 (II), 49.08 (br. s, I and II), 56.81 (I), 56.88 (II), 59.69 (br. s, I and II), 177.16 (I and II), 180.63 (I), 180.78 (II). HRMS (ESI): *m*/*z* [M − Na]^–^ calcd for [C_9_H_24_^11^B_9_N_2_O_3_]^–^: 307.2645, found: 307.2646.

Sodium *N*^α^-(1,2-Dicarba-*nido*-dodecaboran-1-yl)acetyl-(*S*)-lysinate (**14b**). Yield 0.06 g (100%). Colourless solid, mp. 208–213 °C. [α]_D_^20^ −7.0 (*c* 0.4, 1 M NaOH). ^1^H NMR (500 MHz, D_2_O) (diastereomers I and II, appr. 1:1) δ (ppm): −2.77 (br. s, 0.5H, BHB (I)), −2.64 (br. s, 0.5H, BHB (II)), −0.4…2.6 (br. m, 9H, 9 × BH), 1.48–1.60 (m, 2H, 2 × H-4), 1.68–1.80 (m, 3H, 2 × H-5 and H-3^B^), 1.86–1.93 (m, 1H, H-3^A^), 2.10 (s, 0.5H, CH carborane (I)), 2.11 (s, 0.5H, CH carborane (II)), 2.32 (d, *J* = 14.8 Hz, 0.5H, H^B^ acetyl (I)), 2.51 (d, *J* = 15.2 Hz, 0.5H, H^B^ acetyl (II)), 2.57 (d, *J* = 15.2 Hz, 0.5H, H^A^ acetyl (II)), 2.71 (d, *J* = 14.8 Hz, 0.5H, H^A^ acetyl (I)), 3.06 (t, *J* = 7.6 Hz, 2H, 2 × H-6), 4.20–4.25 (m, 1H, H-2), 7.54 (d, *J* = 7.7 Hz, 0.5H, NH (I)), 7.76 (d, *J* = 7.9 Hz, 0.5H, NH (II)). ^11^B{^1^H} NMR (160 MHz, D_2_O) (diastereomers I and II, appr. 1:1) δ (ppm): −37.3 (1B (I and II)), −33.2 (1B (I and II)), −20.7 (1.5B (I and II)), −19.7 (0.5B (II)), −19.1 (0.5B (I)), −18.6 (0.5B (II)), −16.5 (0.5B (I)), −15.7 (0.5B (II)), −13.9 (br. s, 1B (I and II)), −11.8 (1B (I)), −10.8 (1B (II)). ^13^C NMR (126 MHz, D_2_O) (diastereomers I and II) δ (ppm): 24.70 (I), 24.79 (II), 29.13 (I and II), 34.00 (I), 34.10 (II), 42.14 (I and II), 48.03 (I and II), 49.15 (br. s, I and II), 57.03 (I), 57.11 (II), 59.24 (br. s, I), 60.24 (br. s, II), 177.05 (I), 177.11 (II), 181.30 (I), 181.41 (II). HRMS (ESI): *m*/*z* [M − Na]^–^ calcd for [C_10_H_26_^11^B_9_N_2_O_3_]^–^: 321.2803, found: 321.2802.

### 3.3. Cell Lines

The following cell lines were used: BJ-5ta human foreskin fibroblasts (ATCCCRL-4001™), U87 MG human glioblastoma (ATCCHTB-14™), T98G human glioblastoma (ATCCCRL-1690™), SK-Mel-28 human melanoma (ATCCHTB-72™), MDA-MB-231 breast carcinoma (ATCCCRM-HTB-26™), and DU 145 prostate adenocarcinoma (ATCCHTB-81™) stored in the SPF-vivarium cryobank at the Institute of Cytology and Genetics of the Russian Academy of Sciences (Siberian Branch), Novosibirsk. Cells were cultured in 5% CO_2_ in DMEM/F12 (1:1) nutrient medium (Biolot, St. Petersburg, Russia) supplemented with 10% fetal bovine serum (Invitrogen, Waltham, MA, USA). Cells were counted on a Countess automatic cell counter (Invitrogen, Waltham, MA, USA).

### 3.4. MTT Cytotoxicity Assay

Cells were seeded in 96-well plates in the amount of 2 × 10^4^ cells per well and cultivated for 24 h. Stock solutions of compounds **5a** and **5b** in 0.5% aqueous NaHCO_3_ (concentration 5.0 mg/mL) were prepared under stirring for 10 min, then incubated at 37 °C for 20 min, and sonicated on a Sonicator Q700 ultrasonic homogenizer (Qsonica L.C.C, Newtown, CT, USA) for 30 min. A solution of the commercial anticancer agent Cisplatin (Cisplatin Teva, Pharmachemie B.V., Haarlem, the Netherlands) with an initial concentration of 0.50 mg/mL was used as a positive control. Nutrient medium without additives was used as a negative control. The stock solutions of compounds **5a**,**b** were added to the nutrient medium with cells in a volume of 1/5 of the total volume of the medium in the well, as a result of which the concentration of compounds **5a** and **5b** in the medium was 1.00 mg/mL; cisplatin concentration 0.10 mg/mL. Then, a series of twofold dilutions of stock solutions was prepared and added to the nutrient medium with cells in such a way as to obtain nutrient media with the concentration of compounds **5a** and **5b** of 0.50, 0.25, 0.125, 0.063, 0.031, 0.016, and 0.008 mg/mL. The concentration of cisplatin in the positive control samples was 0.10000, 0.0500, 0.0250, 0.0125, 0.0063, 0.0031, 0.0016, and 0.0008 mg/mL. The duration of cell incubation was 3 days at 37 °C in an atmosphere containing 5% CO_2_. After that, the culture medium was removed from each well, a solution of MTT [3-(4,5-dimethylthiazol-2-yl)-2,5-diphenyltetrazolium bromide] in DMEM/F12 (1:1) culture medium (MTT concentration 0.5 mg/mL) was added and incubated for 4 h; then the supernatant was removed and the formazan precipitate was dissolved in DMSO (100 µL). The optical density of the resulting solutions was determined on a Multiskan Sky High Microplate Spectrophotometer (Thermo Fisher Scientific, Waltham, MA, USA) at a wavelength of 595 nm. Cell viability was determined based on optical density; cell viability in the negative control was taken as 100%. Experiments were performed in three parallel runs (for detailed information on cell viability of various cell lines, see the Supporting Information, Table S1).

### 3.5. Boron Uptake and Accumulation Assay

Stock solution of compound **5b** was prepared in 0.5% aqueous NaHCO_3_ (concentration 5.0 mg/mL). BJ-5ta, SK-Mel 28, T98G, DU 145, MDA-MB-231, and U87 MG cells were cultured in 5 mL of nutrient medium at 37 °C in a 5% CO_2_ atmosphere until a monolayer was obtained (from 3 × 10^6^ up to 5 × 10^6^ cells). The nutrient medium was removed, a mixture of the nutrient medium (4.75 mL in the case of U87 MG cells or 4.50 mL in other cases) and the stock solution of compound **5b** (0.25 mL in the case of U87 MG cells or 0.50 mL in other cases) was added to the cells and incubated at 37 °C in a 5% CO_2_ atmosphere. Cells cultured without the addition of test compound were used as controls.

After the cells were incubated for various times (10 min, 30 min, 1 h, 3 h, 6 h, and 8 h), the culture medium was separated from the cells, and the cells were removed from the substrate with a trypsin–versene solution (1:1) (Biolot, St. Petersburg, Russia), and the number of cells was counted (the number of cells used for assay, see the Supplementary Materials, Table S2). The resulting cell suspension was divided into three equal parts, centrifuged (1000 rpm, 5 min), the cells were separated from the supernatant. 16 *M* Nitric acid (1.0 mL) was added to the resulting cells, the mixture was kept at 95 ± 1 °C for 30–40 min, then cooled to 20 °C, and deionized water (3.0 mL) was added. The boron content in the obtained solutions was determined on an iCAP 6500 DUO high-resolution atomic emission spectrometer with inductively coupled plasma (Thermo Fisher Scientific, Waltham, MA, USA) according to the procedure described in [132].

## 4. Conclusions

Thus, we synthesized for the first time the (*S*)-lysine and (*S*)-ornithine derivatives containing a (*nido*-carboran-7-yl)acetic acid moiety at the amino group either in the side chain or at the alpha position of amino acid. Sodium salts of *nido*-carboranyl derivatives of lysine and ornithine are soluble in water (5–7 mg/mL) and exhibited moderate cytotoxicity against BJ-5ta human foreskin fibroblasts and tumour cells of various lines. It has been shown that sodium salt of *N*^ε^-(*nido*-carboran-7-yl)acetyl-(*S*)-lysine accumulates well in MDA-MB-231 (human breast carcinoma) and SK-Mel 28 (human melanoma) cell lines, providing a boron concentration of up to 0.67 µg/10^6^ cells in in vitro experiments. The obtained results form the basis for further testing of toxicity and biodistribution in experiments on laboratory animals.

## Figures and Tables

**Table 1:** CC_50_ values (mg/mL) for sodium salts of *nido*-carborane-containing amino acids **5a** and **5b** after a 72 h incubation with cells (MTT assay, *n* = 3).

Compound	Cell Line					
	BJ-5ta	DU 145	MDA-MB-231	SK-Mel 28	T98G	U87MG
**5a**	0.82	1.00	0.91	1.76	n.d.	n.d.
**5b**	1.78	0.57	0.68	0.56	0.27	0.45

n.d.—not determined.

## Data Availability

Data are contained within the article and Supplementary Materials.

## Supplementary Materials

The following supporting information can be downloaded at https://www.mdpi.com/article/10.3390/ijms26178560/s1.
