# Supplementary material for: Nido-Carborane Derivatives of (S)-Ornithine and (S)-Lysine as Potential Boron Delivery Agents: Synthesis and In Vitro Evaluation
Source: Int J Mol Sci. 2025 Sep 3;26(17):8560. doi: 10.3390/ijms26178560 (PMC12428867; doi:10.3390/ijms26178560)
Supplement: Supplementary file 1 [file ijms-26-08560-s001.zip › ijms-3810010-supplementary.pdf]

Supplementary Materials

nido-Carborane Derivatives of (*S*)-Ornithine and (*S*)-Lysine as Potential Boron Delivery Agents: Synthesis and In Vitro Evaluation

Dmitry A. Gruzdev [1*], Galina L. Levit [1], Vera V. Musiyak [1], Angelina A. Telegina [1],
Ilya N. Ganebnykh [1], Marina A. Ezhikova [1], Mikhail I. Kodess [1],
Olga I. Solovieva [2,3], Tatiana Ya. Gusel'nikova [3], Ivan A. Razumov [2,3] and
Victor P. Krasnov [1]

[1] Postovsky Institute of Organic Synthesis, Russian Academy of Sciences (Ural Branch), Ekaterinburg 620066, Russia

[2] Institute of Cytology and Genetics, Russian Academy of Sciences (Siberian Branch), Novosibirsk 630090, Russia

[3] Department of Physics, Novosibirsk State University, Novosibirsk 630090, Russia

Contents

NMR Spectra S2

HRMS Data S-25

Table S1. Effect of Compounds **5a** and **5b** and Cisplatin (Positive Control) on the Viability of Various Cell Lines In Vitro within 72 h of Co-incubation S30

Table S2. Boron Accumulation after Incubation in the Presence of Compound **5b** .. S33

NMR SPECTRA

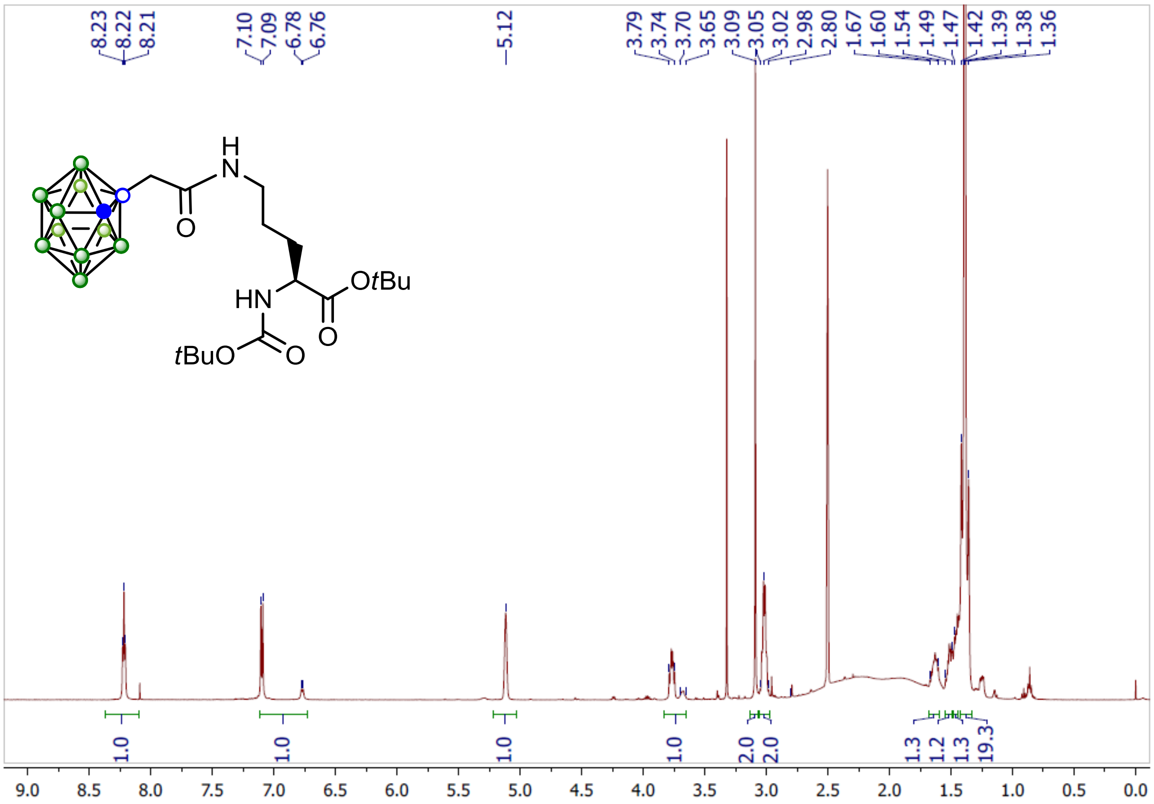

Figure S1. ^{1}H NMR spectrum of compound **3a** (500 MHz, DMSO-d_6)

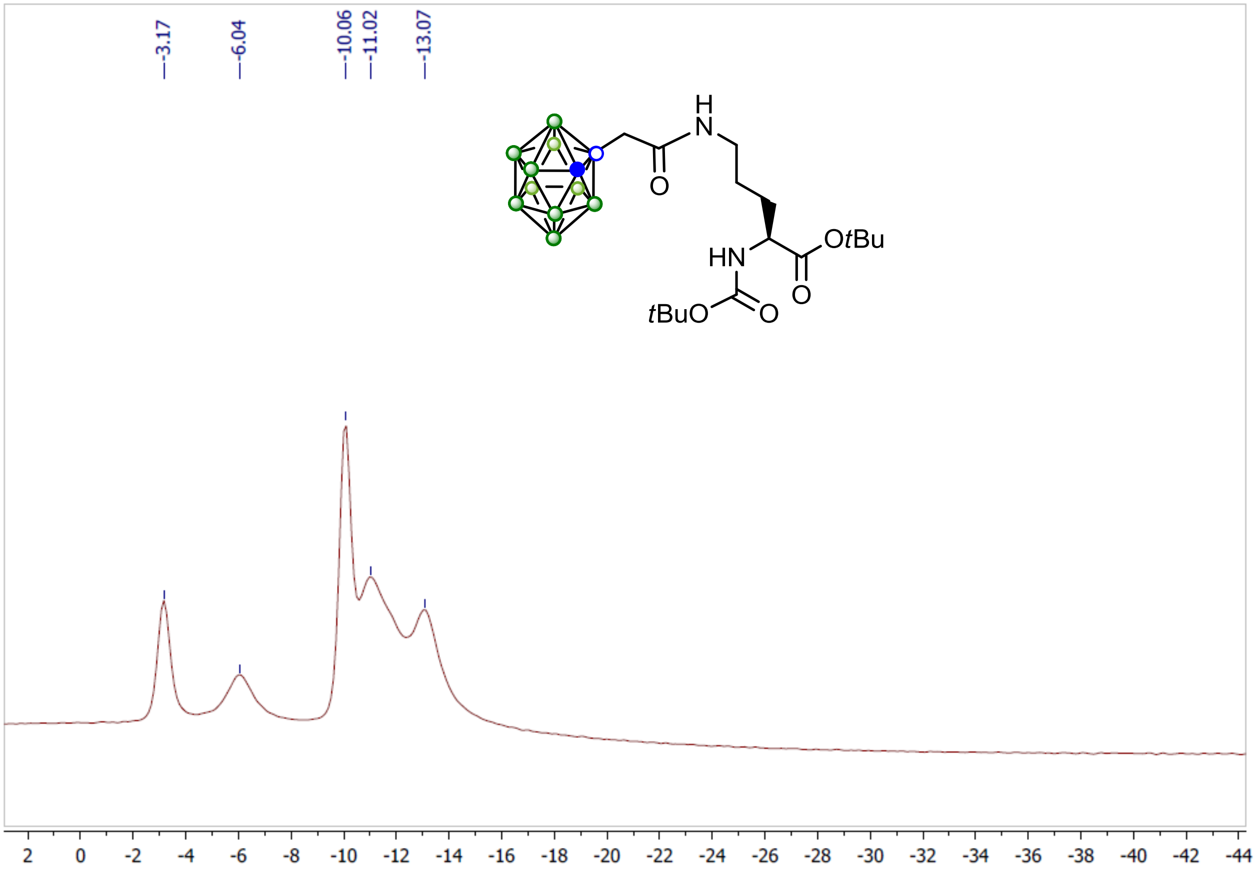

Figure S2. ^{11}B{^{1}H} NMR spectrum of compound **3a** (160 MHz, DMSO-d_6)

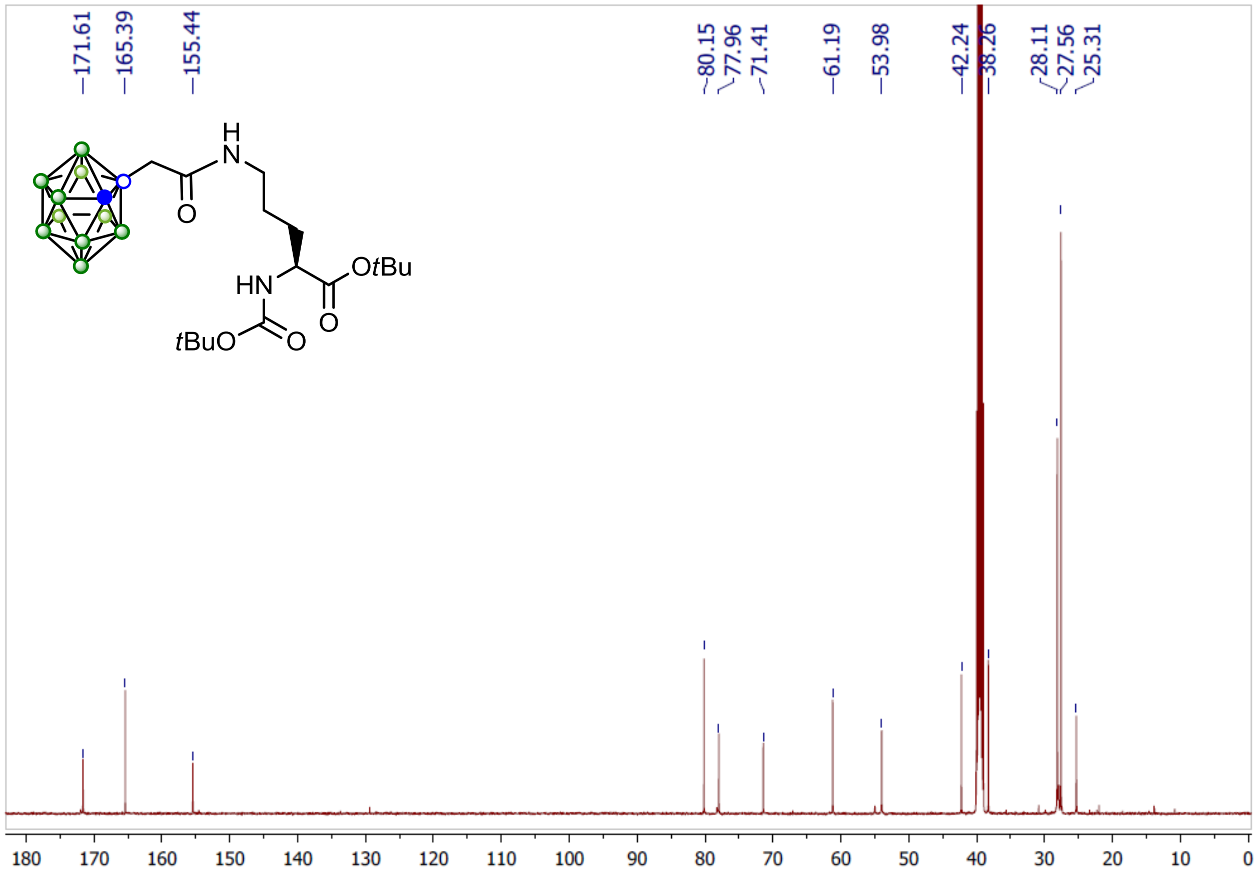

Figure S3. ^{13}C NMR spectrum of compound **3a** (126 MHz, DMSO-d_6)

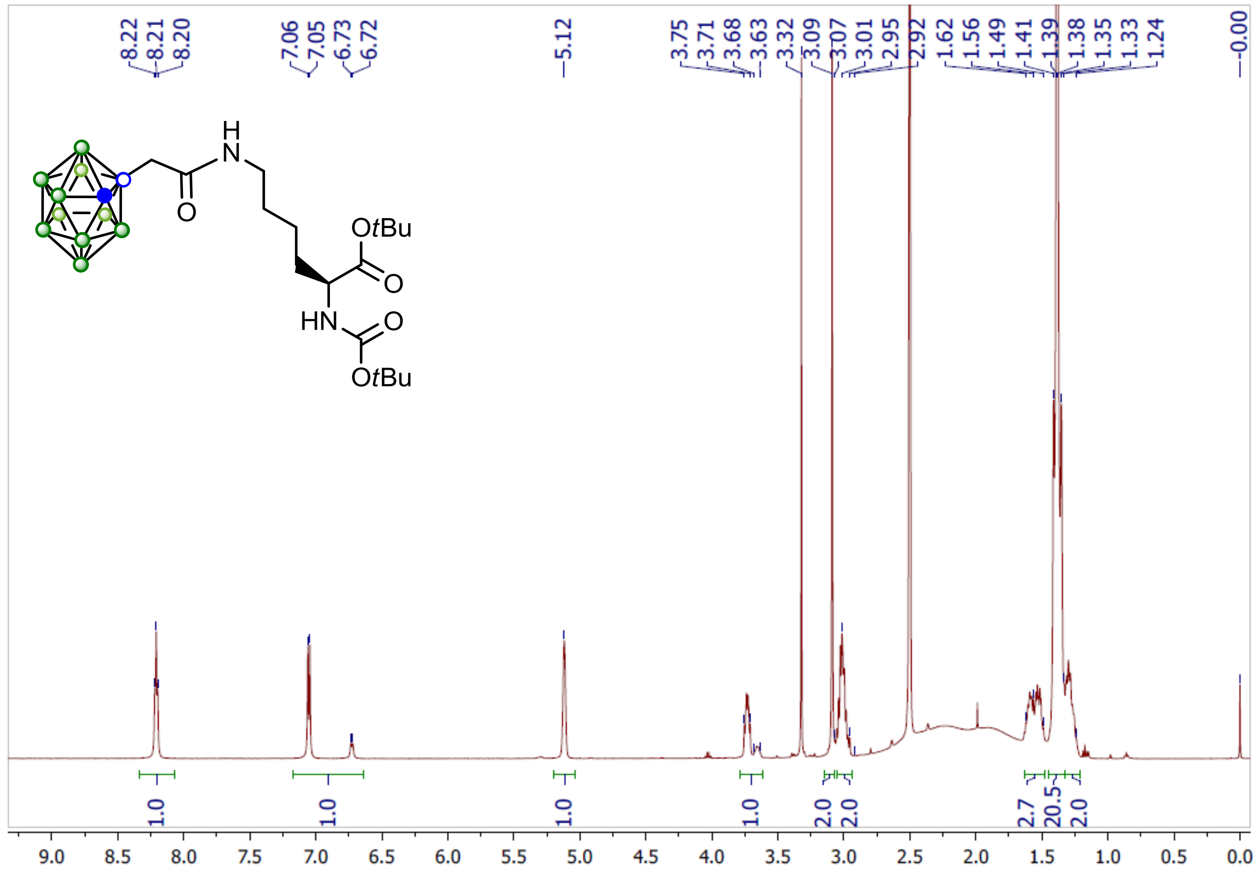

Figure S4. ^{1}H NMR spectrum of compound **3b** (500 MHz, DMSO-d_6)

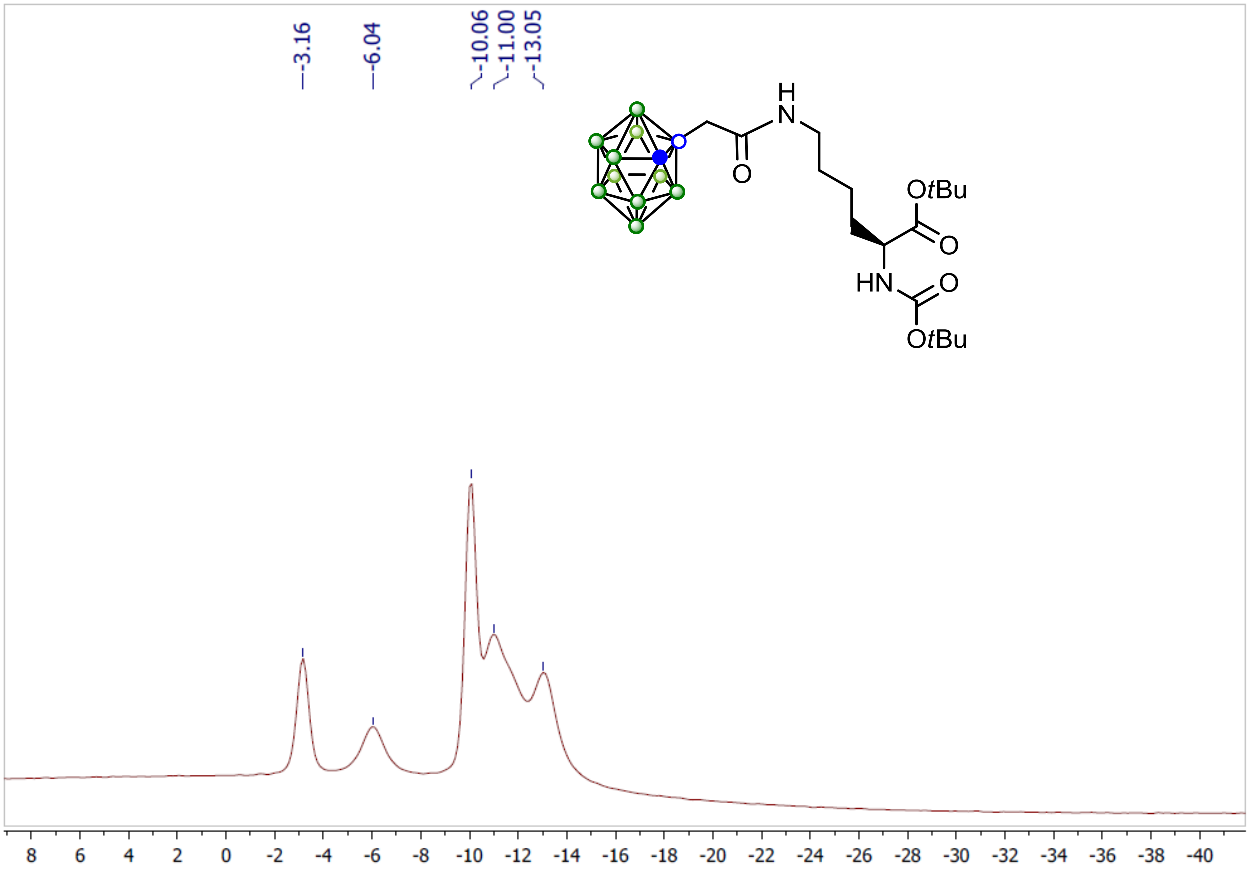

Figure S5. $^{11}B\{^{1}H\}$ NMR spectrum of compound **3b** (160 MHz, DMSO-d_6)

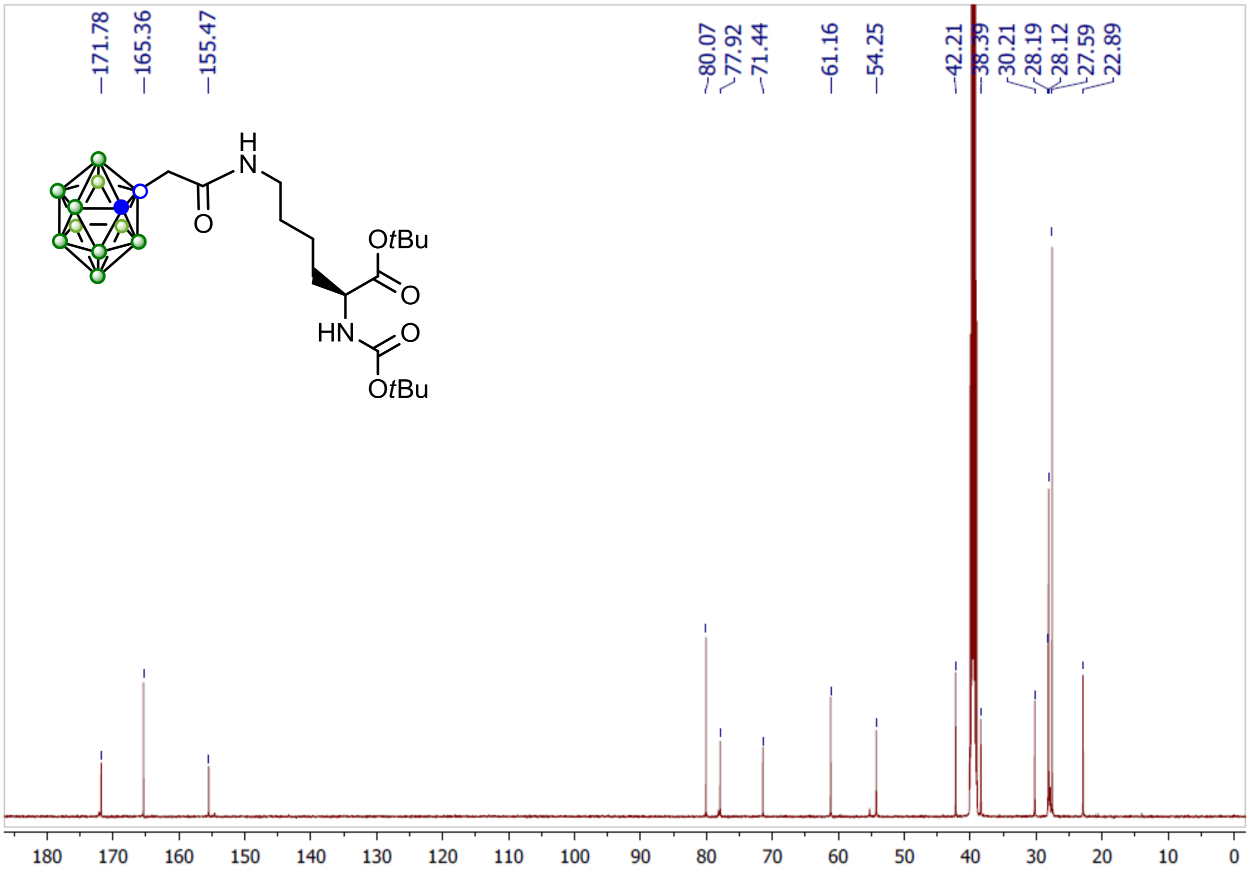

Figure S6. ^{13}C NMR spectrum of compound **3b** (126 MHz, DMSO-d_6)

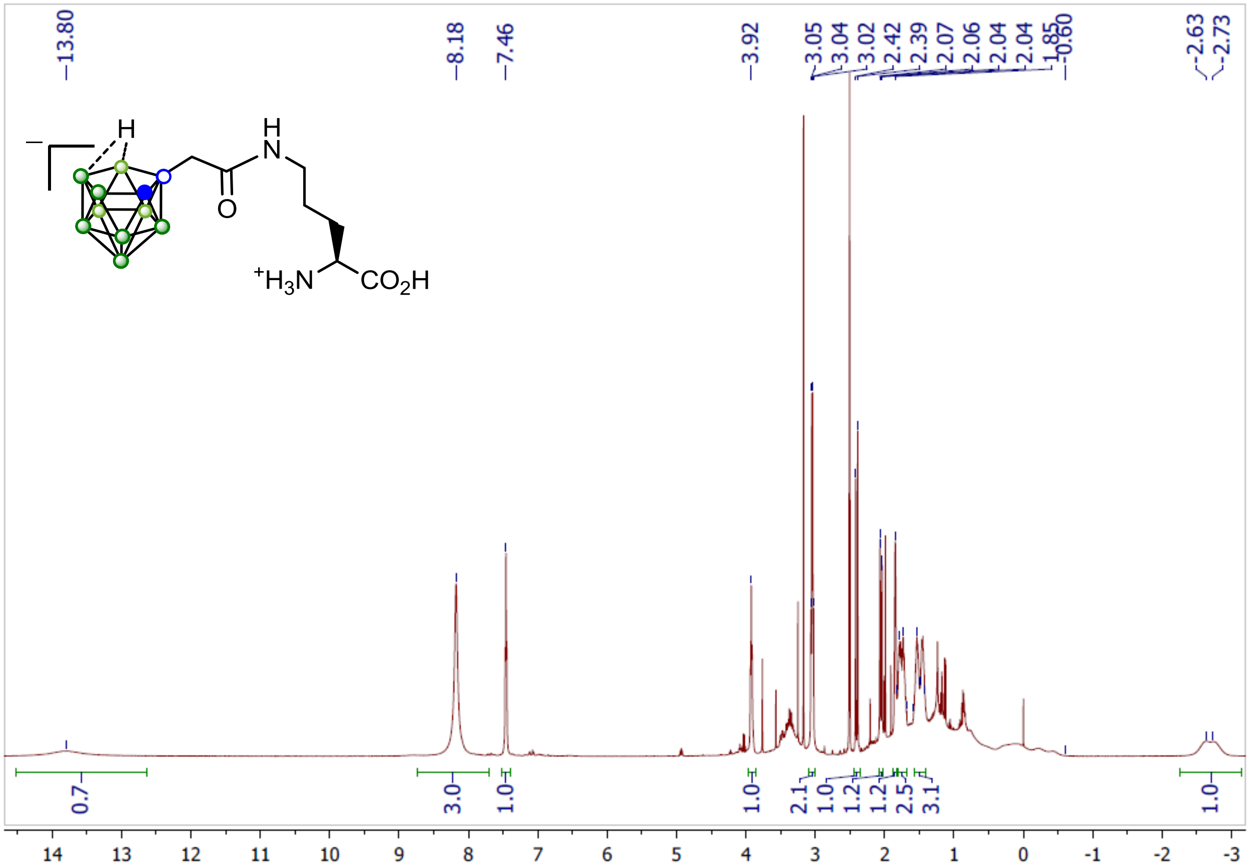

Figure S7. ^{1}H NMR spectrum of compound **4a** (500 MHz, DMSO-d_6)

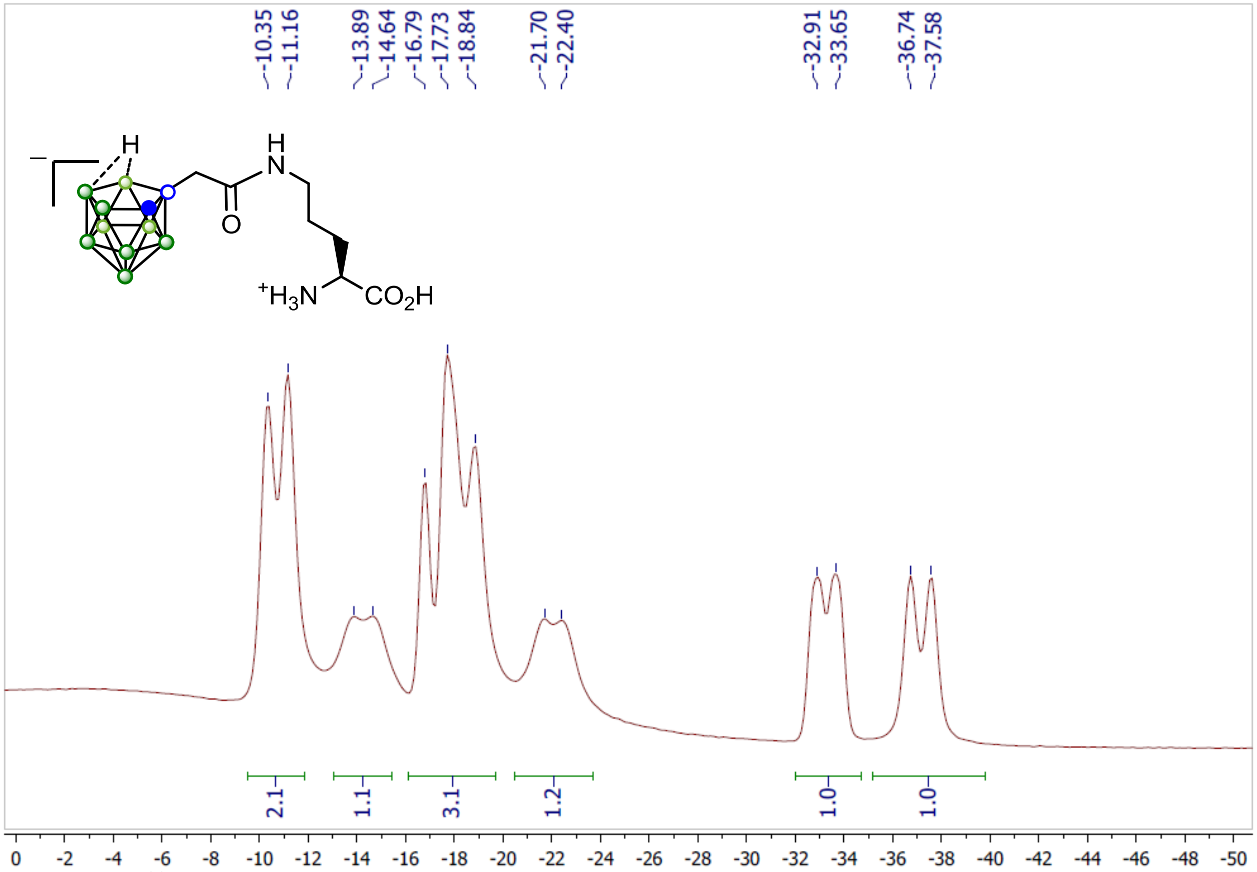

Figure S8. ^{11}B NMR spectrum of compound **4a** (160 MHz, DMSO-d_6)

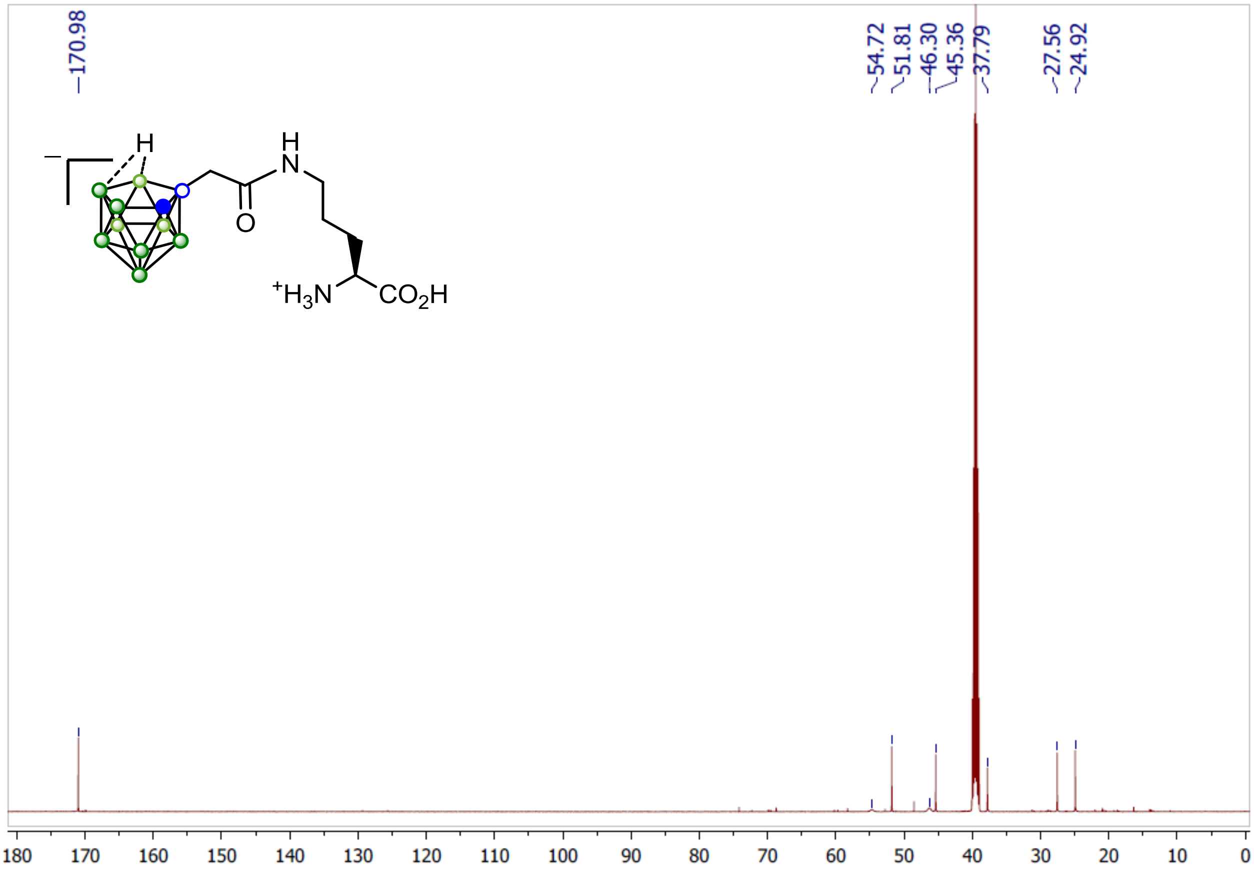

Figure S9. ^{13}C NMR spectrum of compound **4a** (126 MHz, DMSO-d_6)

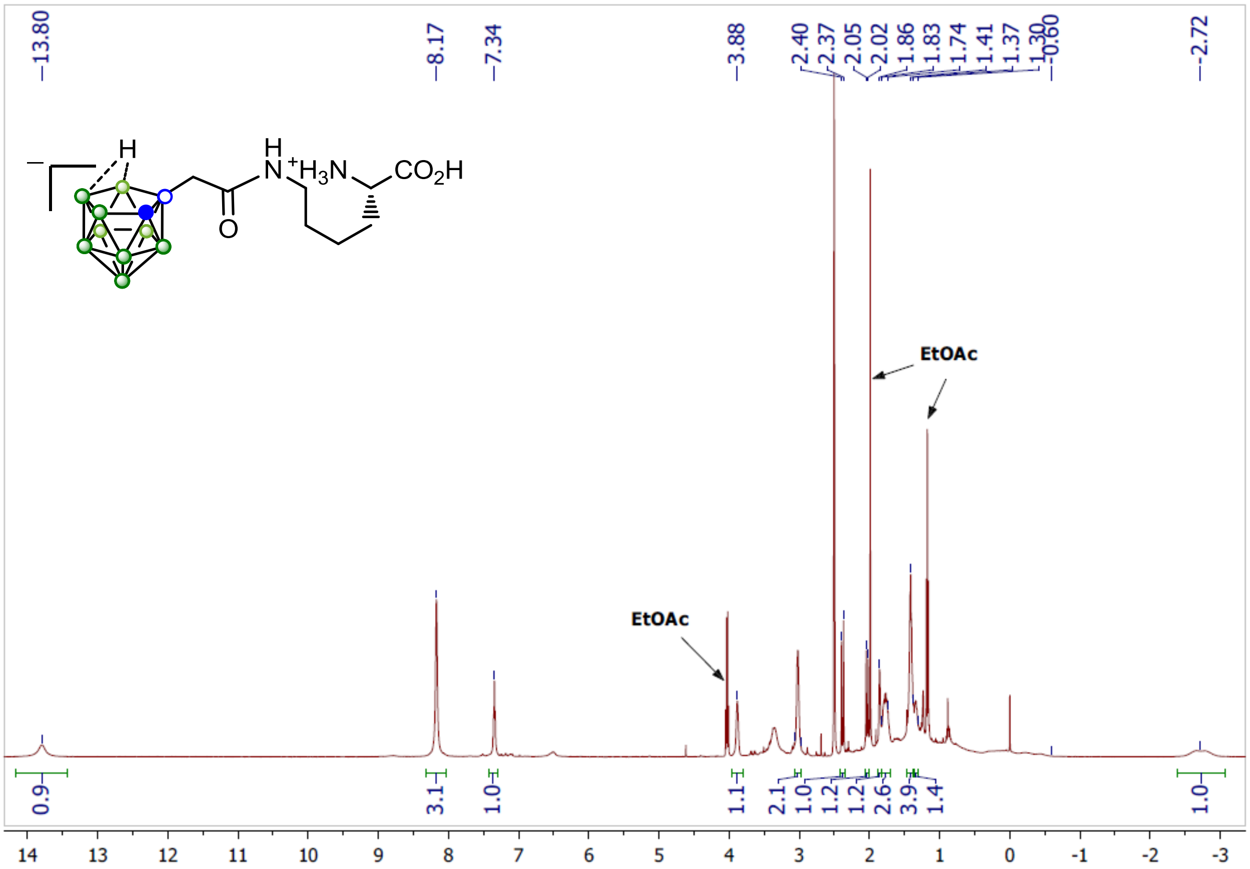

Figure S10. ^{1}H NMR spectrum of compound **4b** (500 MHz, DMSO-d_6)

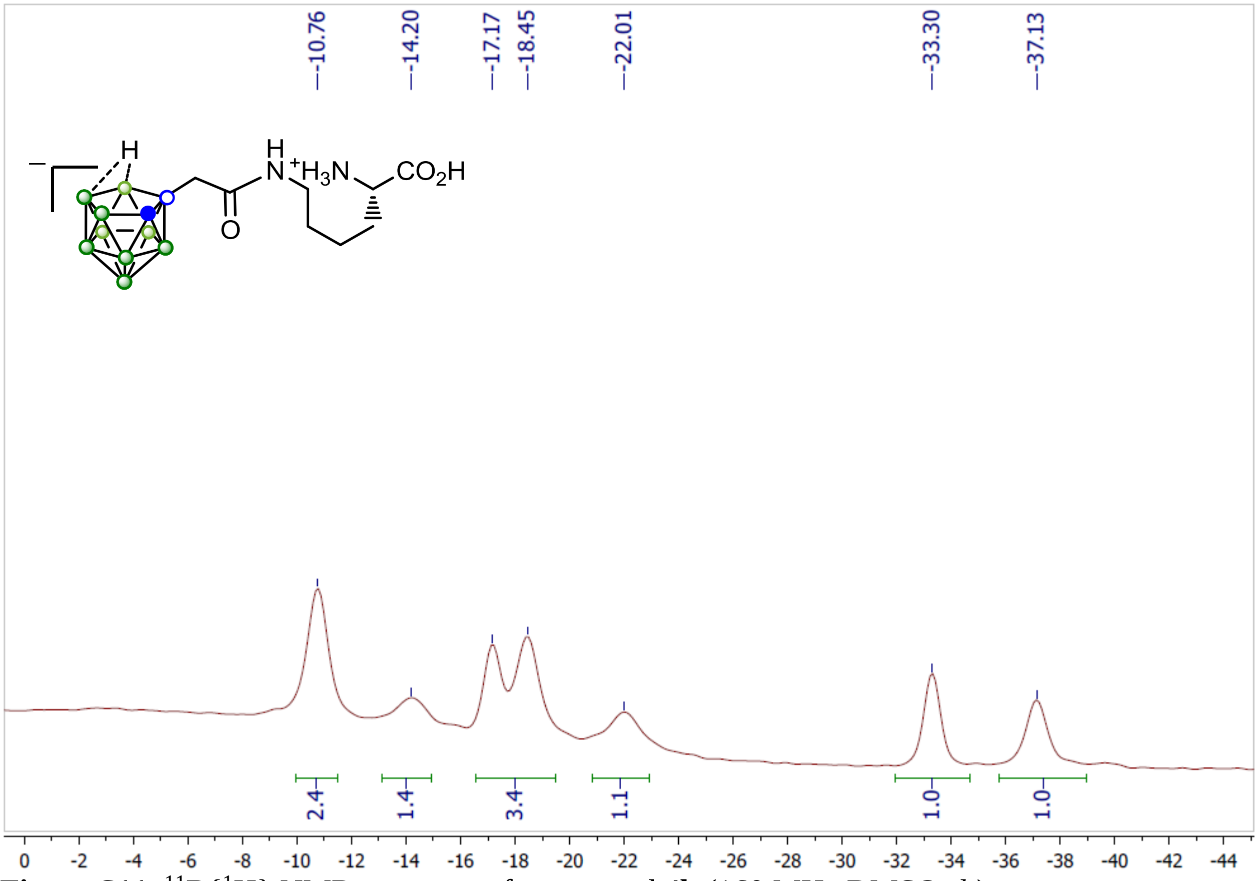

Figure S11. $^{11}B\{^{1}H\}$ NMR spectrum of compound **4b** (160 MHz,DMSO-d_6)

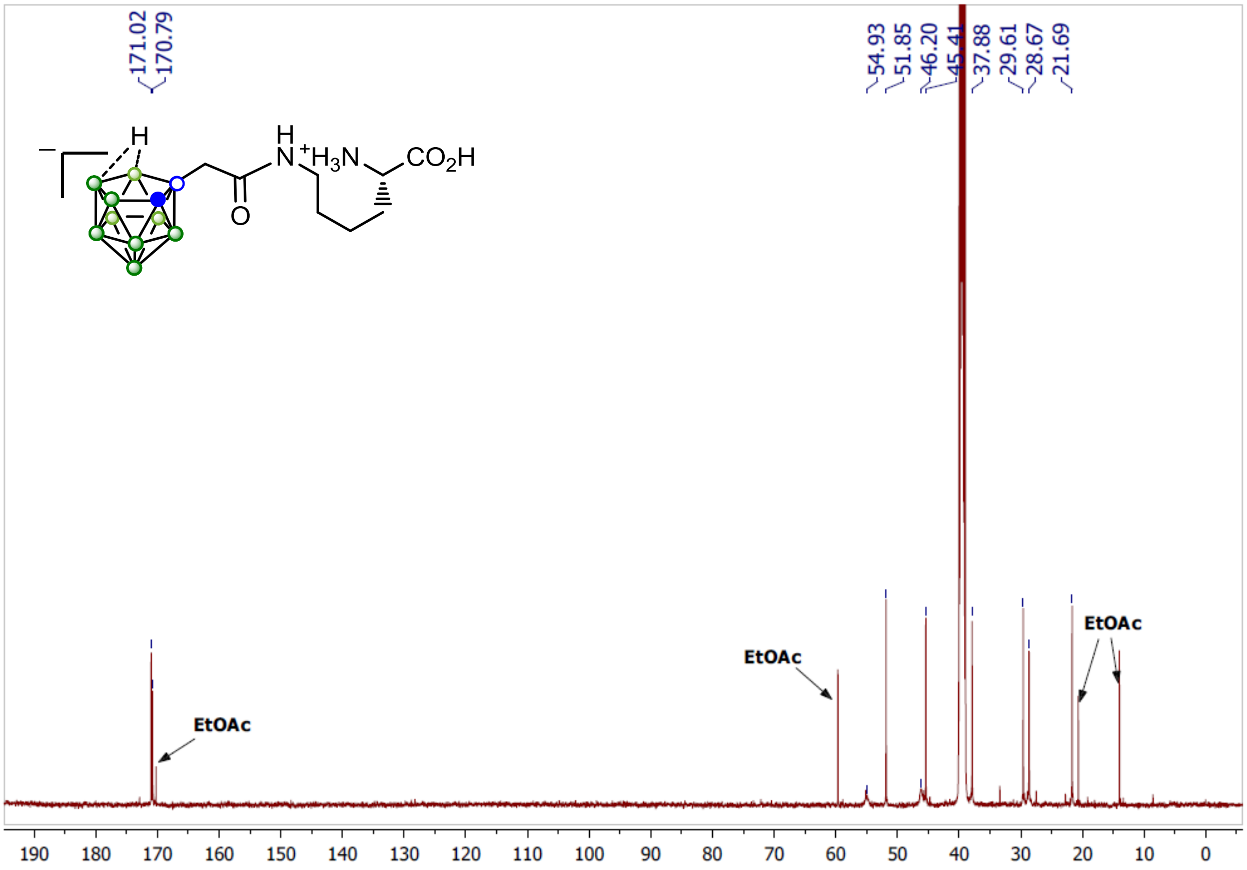

Figure S12. ^{13}C NMR spectrum of compound **4b** (126 MHz, DMSO-d_6)

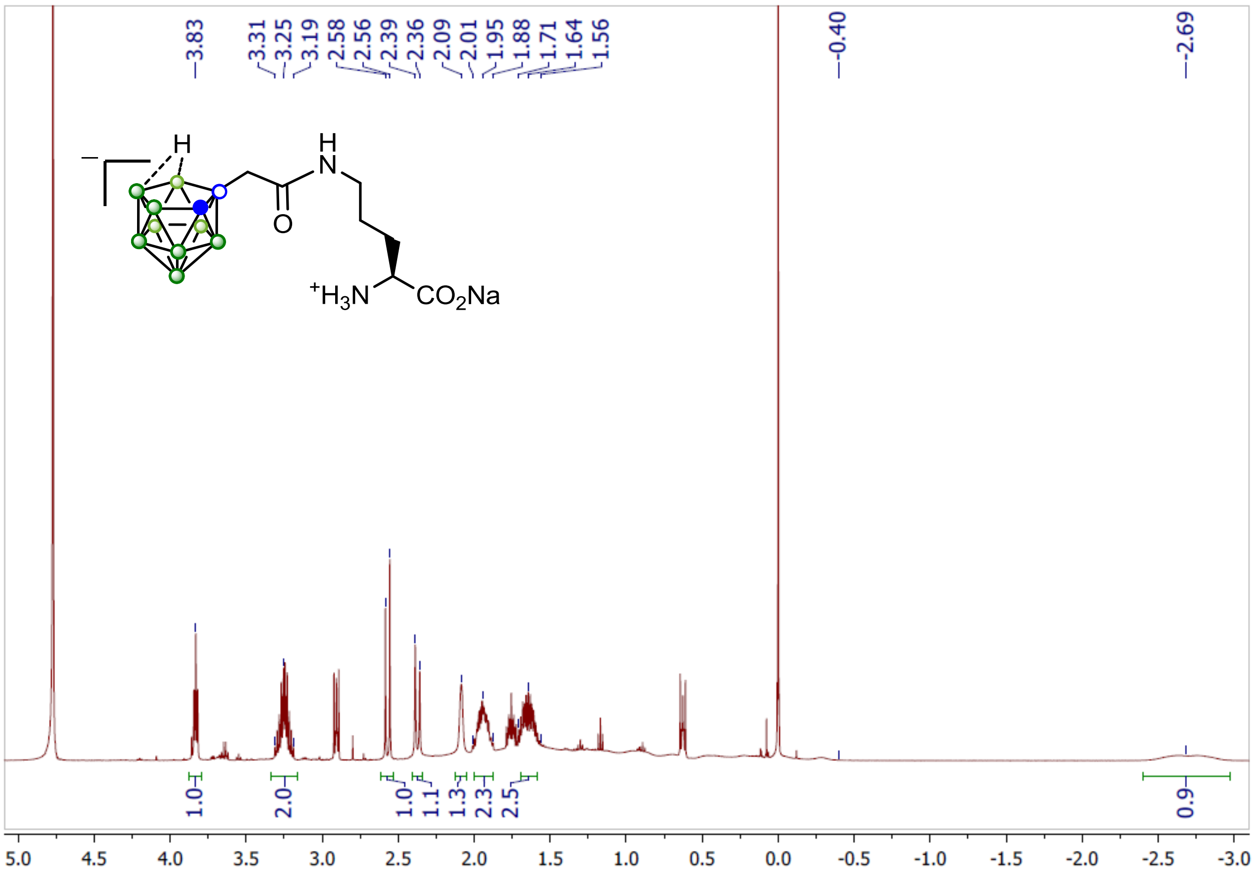

Figure S13. ^{1}H NMR spectrum of compound **5a** (500 MHz, D_2O)

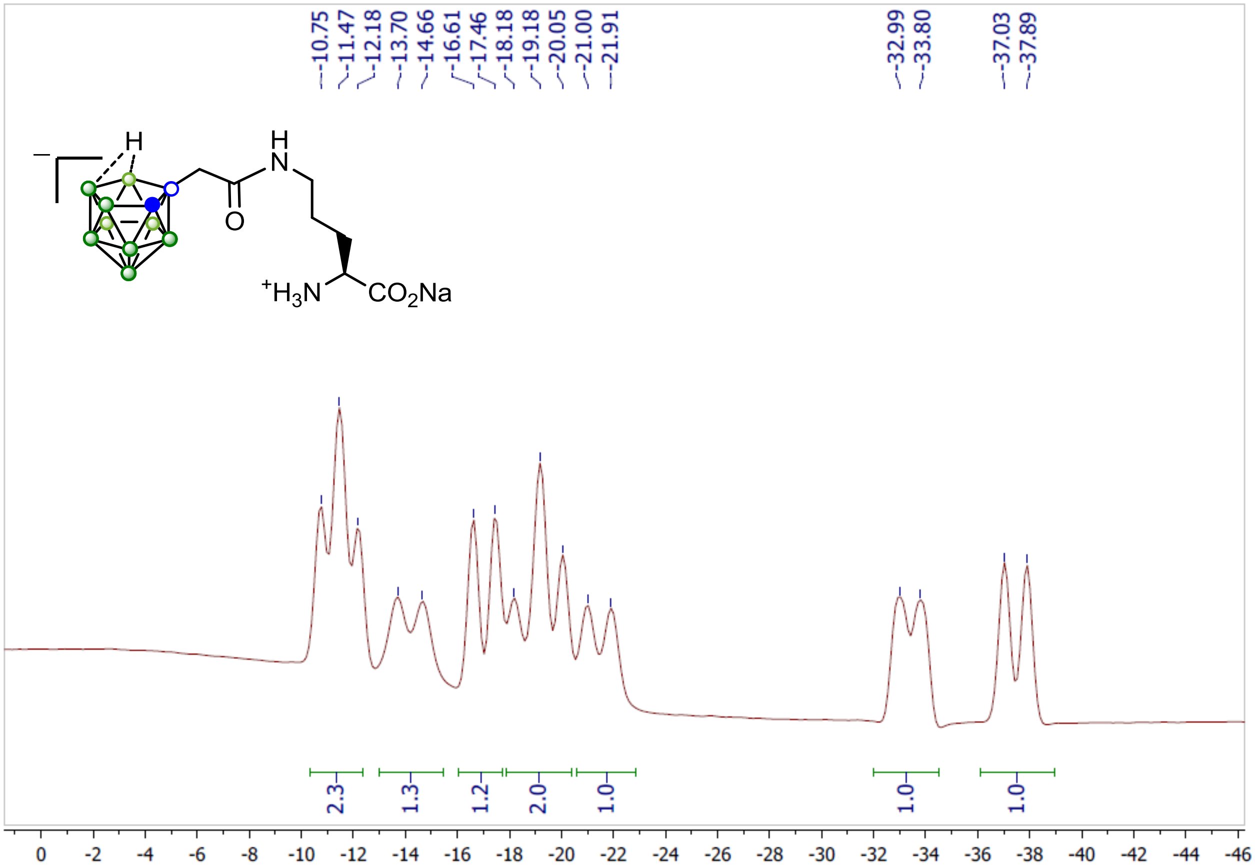

Figure S14. ^{11}B NMR spectrum of compound **5a** (160 MHz, D_2O)

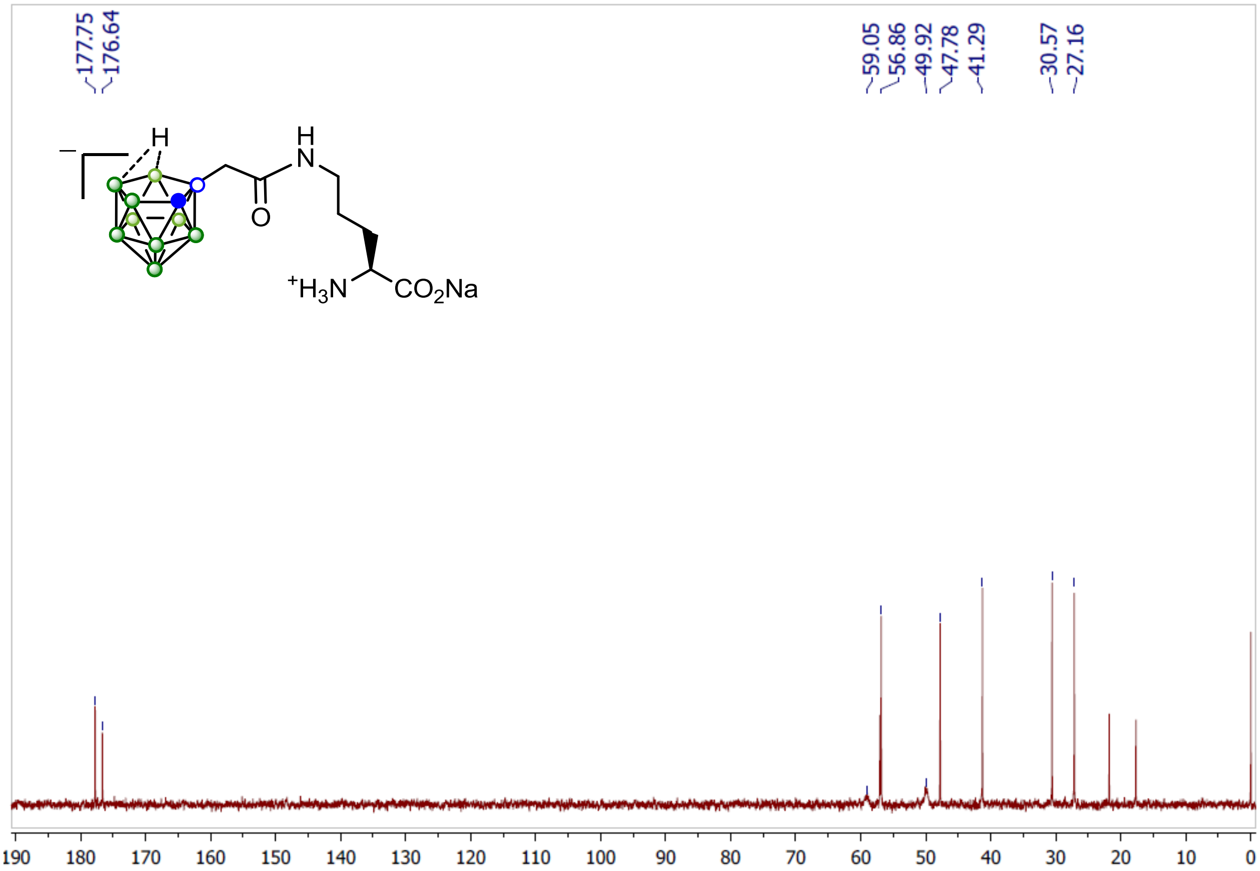

Figure S15. ^{13}C NMR spectrum of compound **5a** (126 MHz, D_2O)

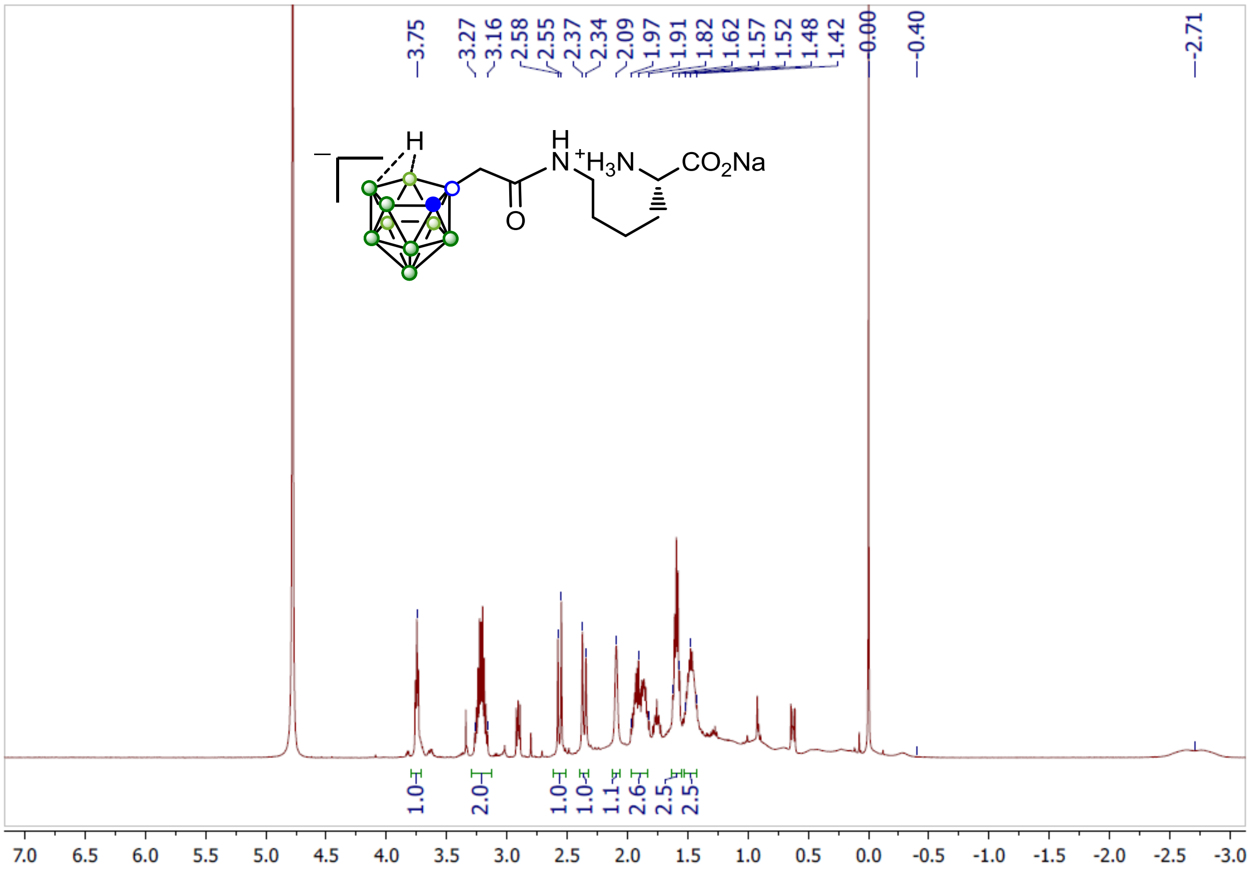

Figure S16. ^{1}H NMR spectrum of compound **5b** (500 MHz, D_2O)

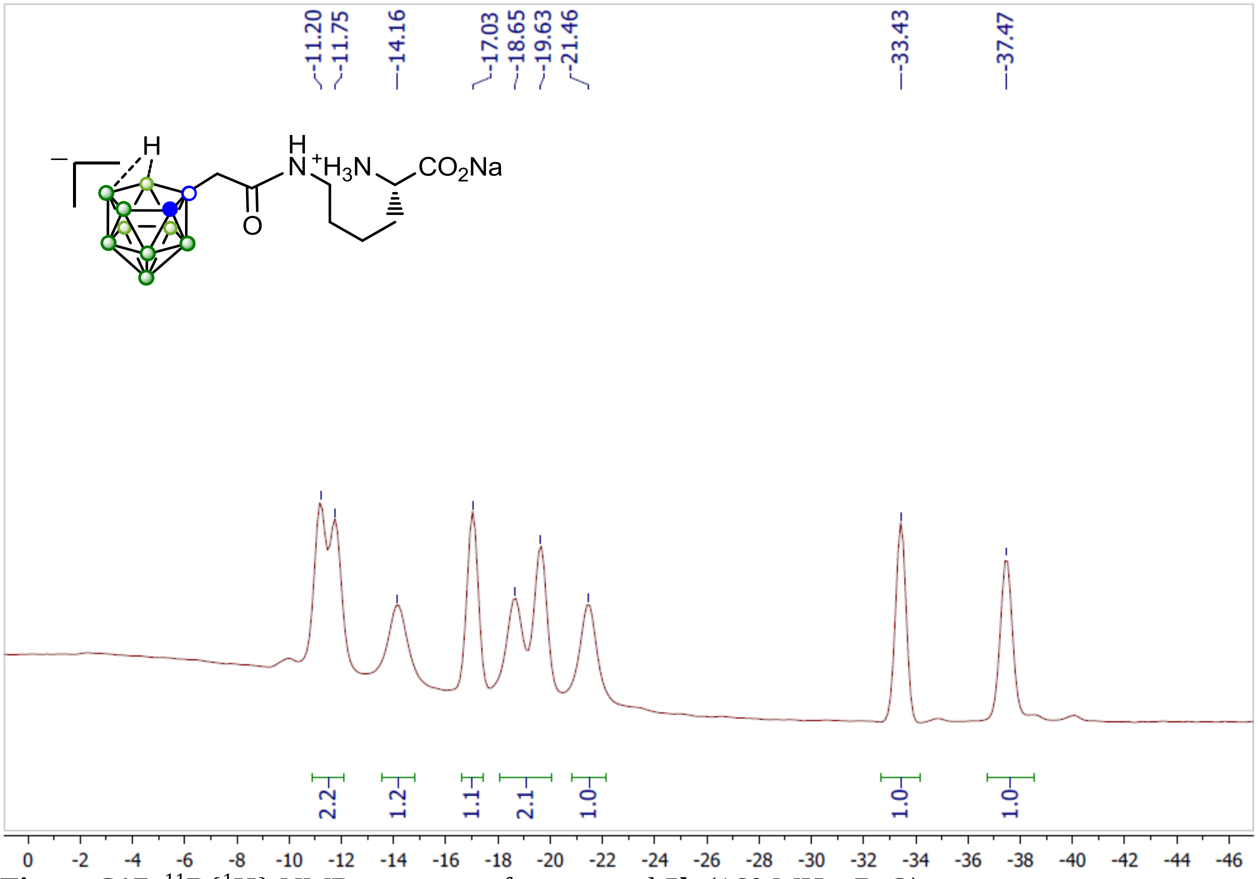

Figure S17. $^{11}B\{^{1}H\}$ NMR spectrum of compound **5b** (160 MHz, D_2O)

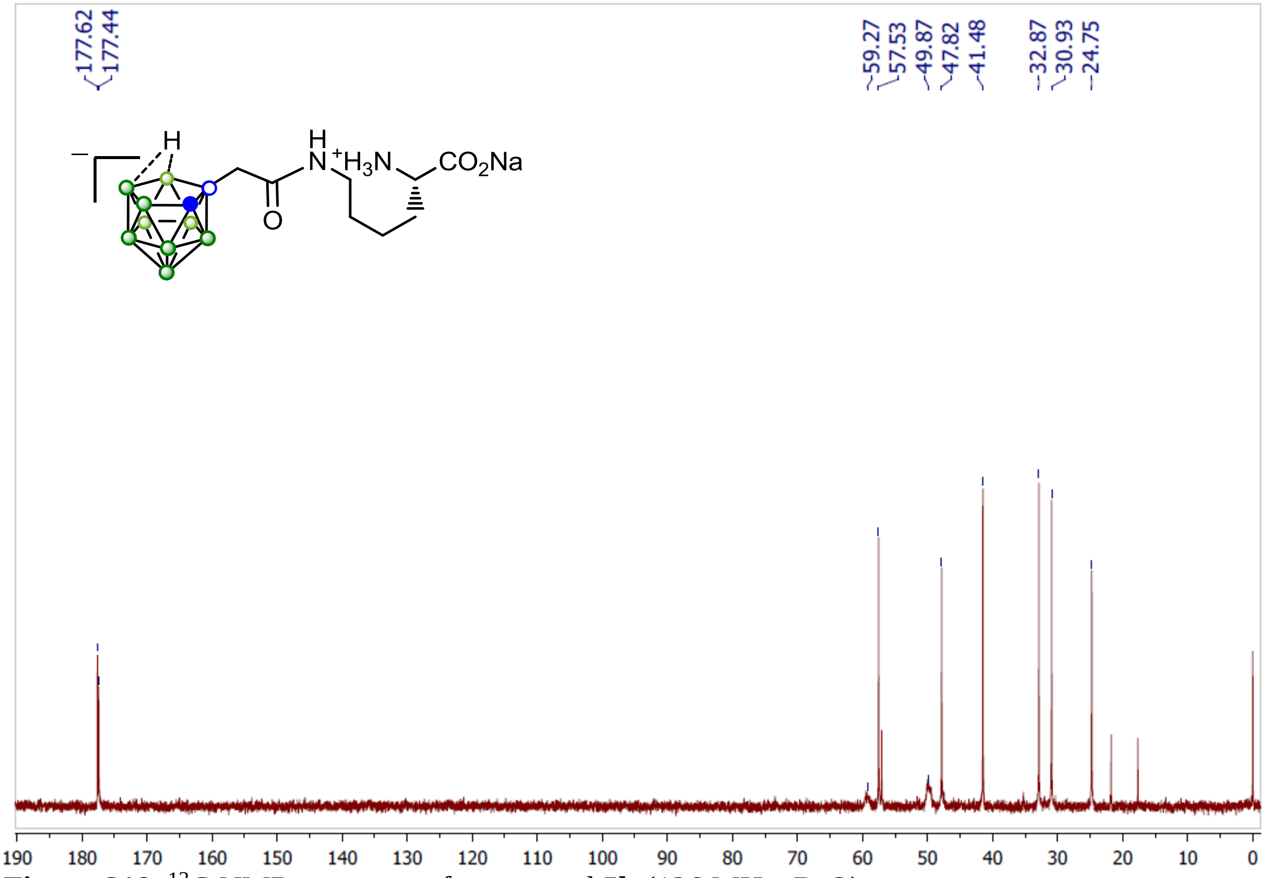

Figure S18. ^{13}C NMR spectrum of compound **5b** (126 MHz, D_2O)

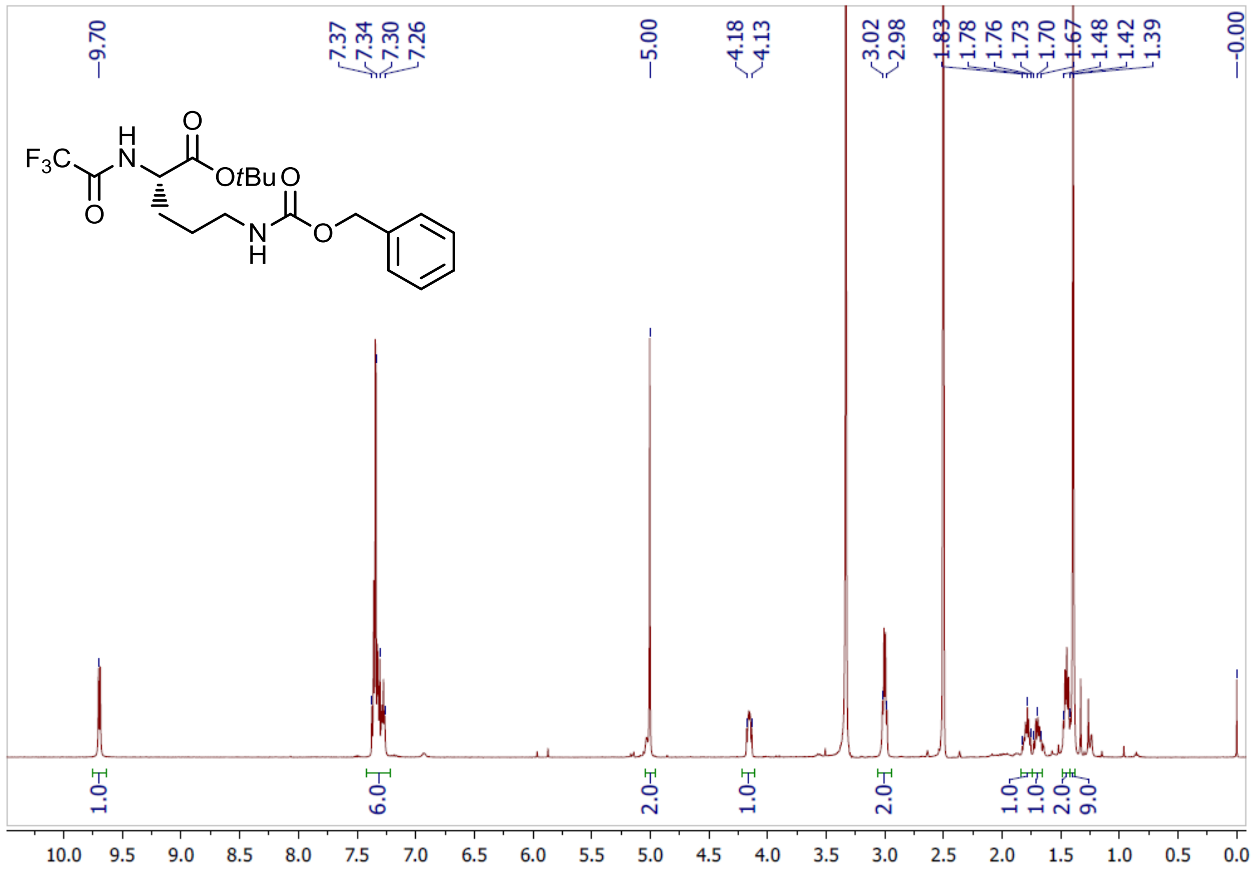

Figure S19. ^{1}H NMR spectrum of compound **7** (500 MHz, DMSO-d_6)

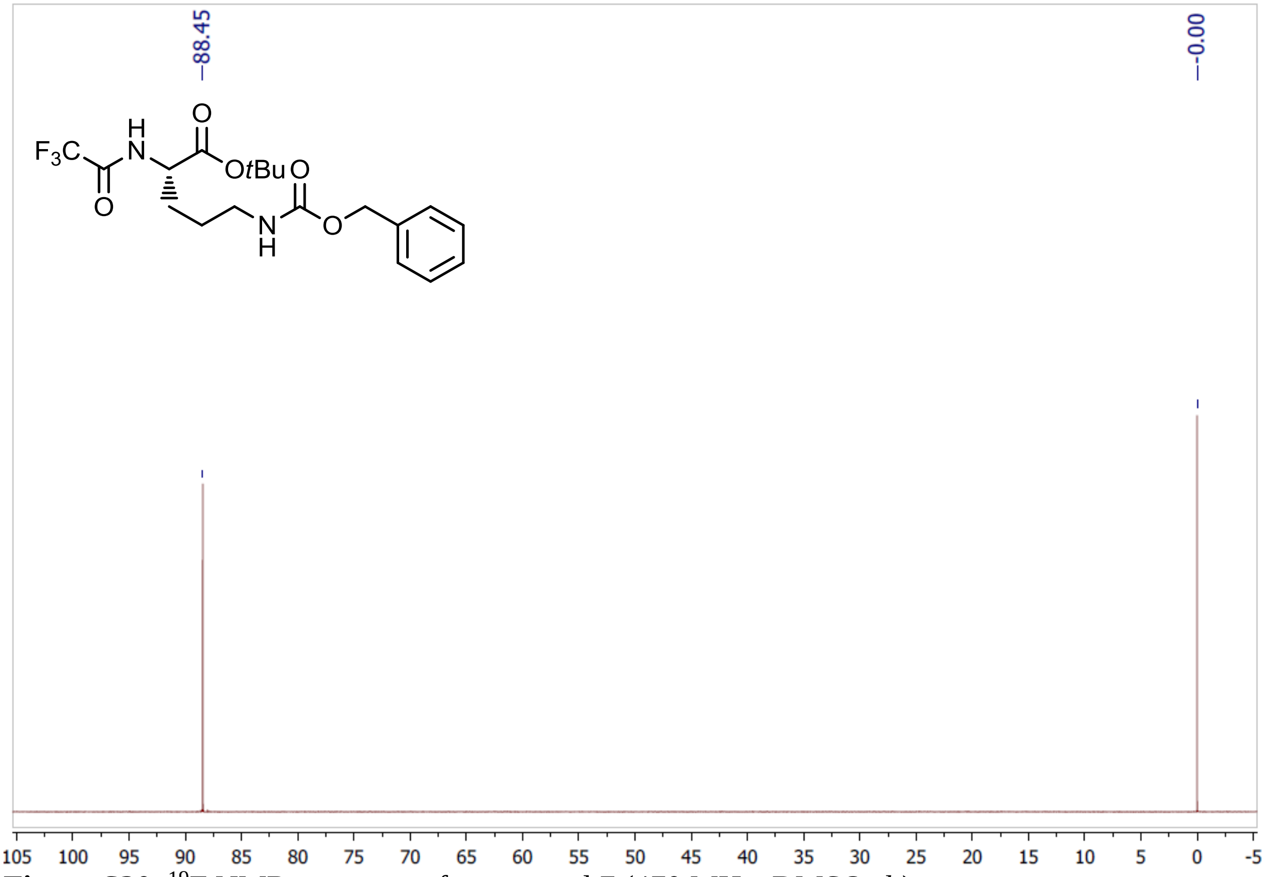

Figure S20. ^{19}F NMR spectrum of compound **7** (470 MHz, DMSO-d_6)

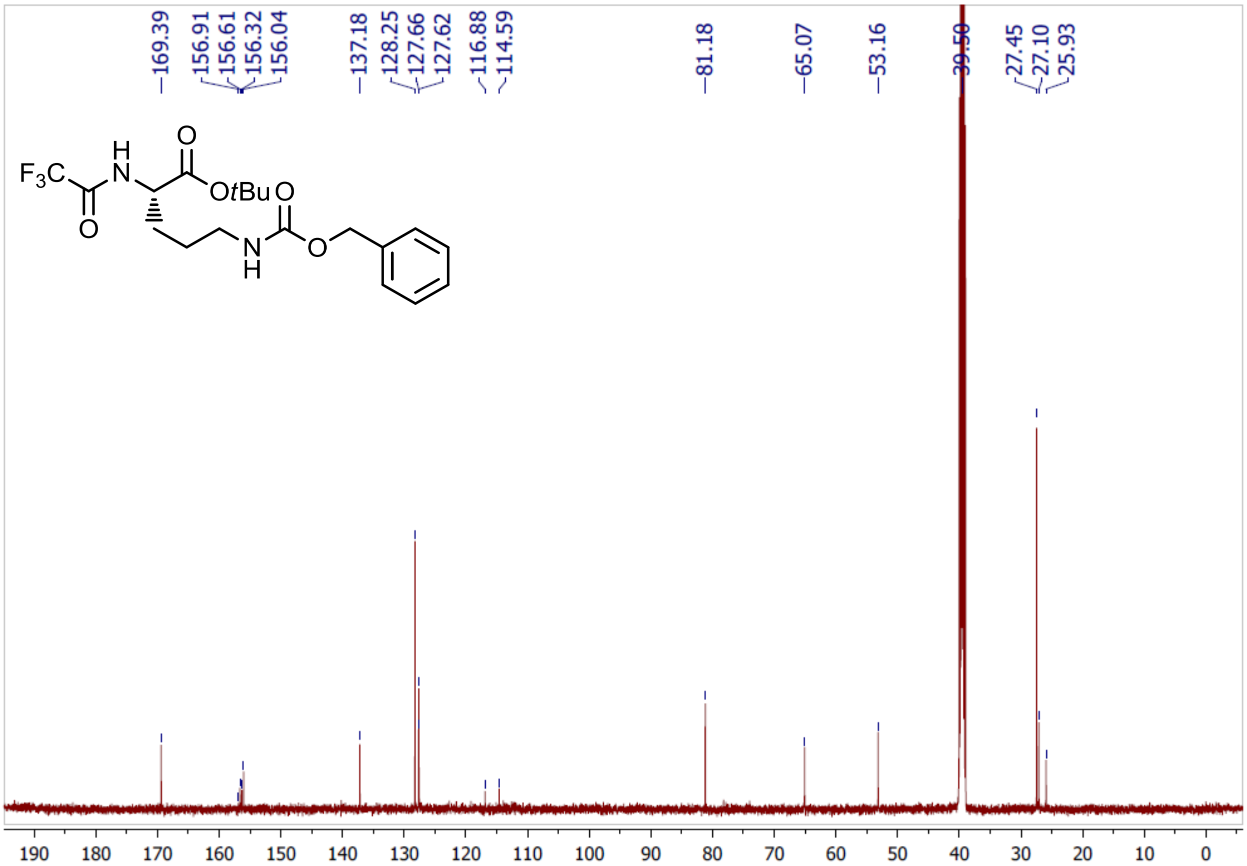

Figure S21. ^{13}C NMR spectrum of compound **7** (126 MHz, DMSO-d_6)

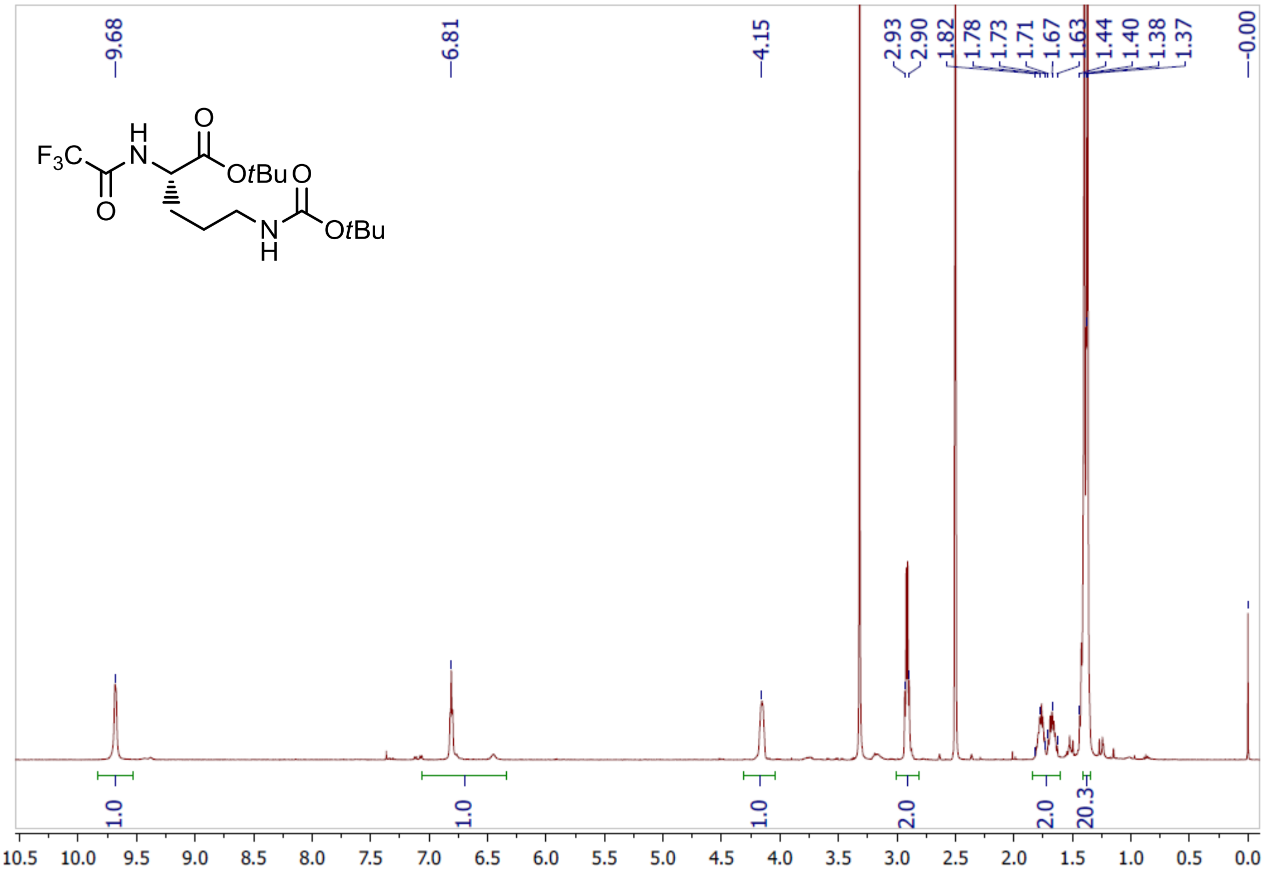

Figure S22. ^{1}H NMR spectrum of compound **8** (500 MHz, DMSO-d_6)

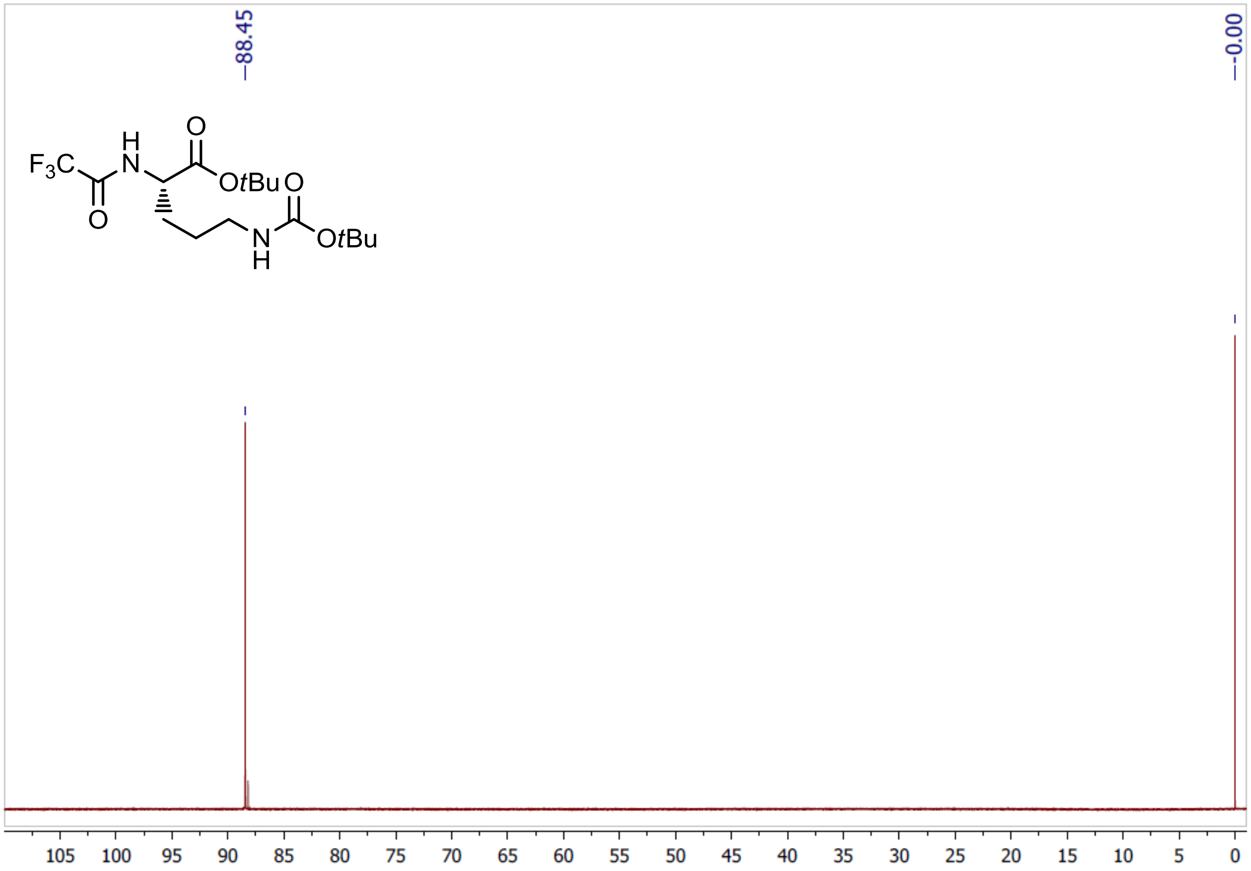

Figure S23. ^{19}F NMR spectrum of compound **8** (470 MHz, DMSO-d_6)

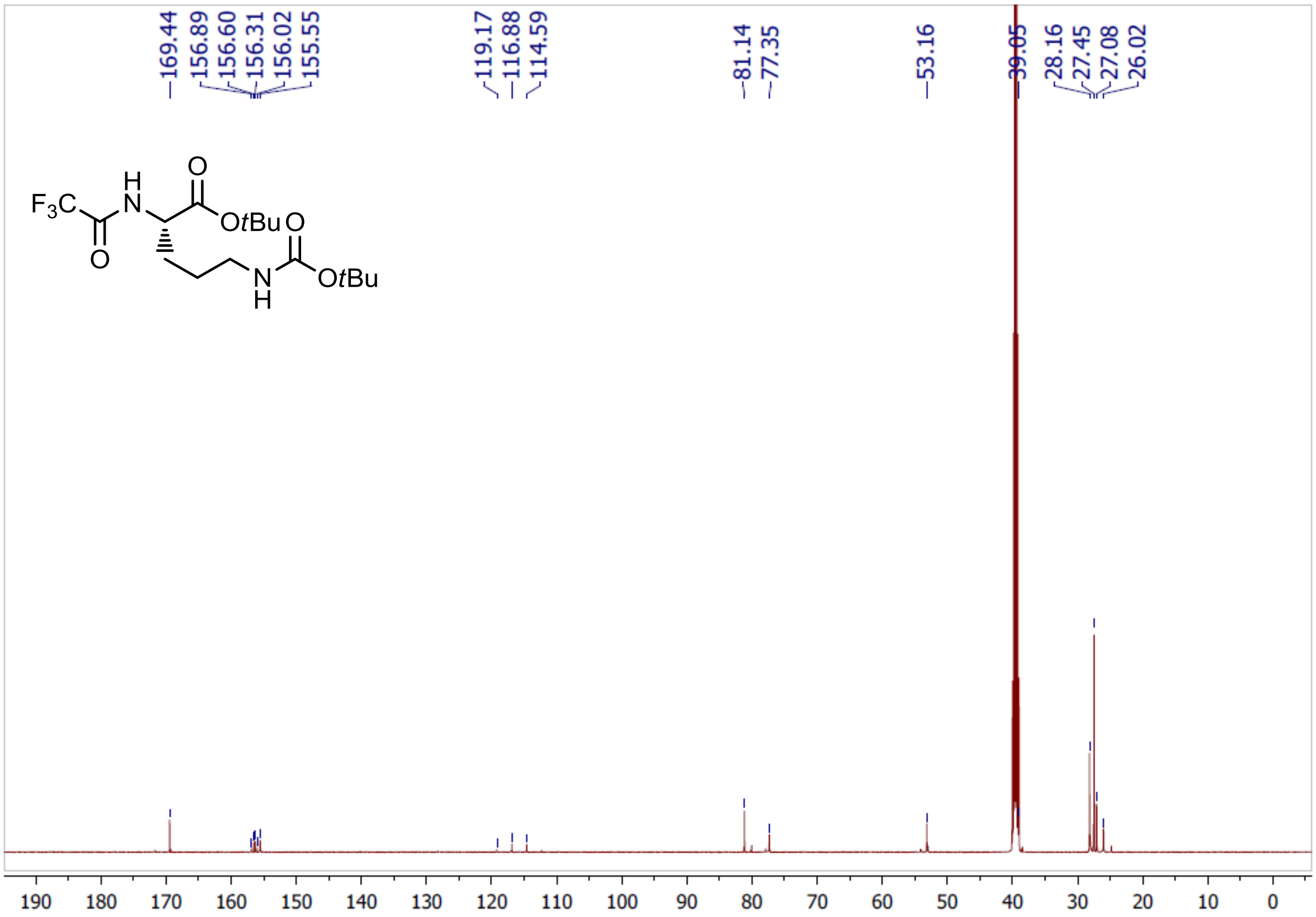

Figure S24. ^{13}C NMR spectrum of compound **8** (126 MHz, DMSO-d_6)

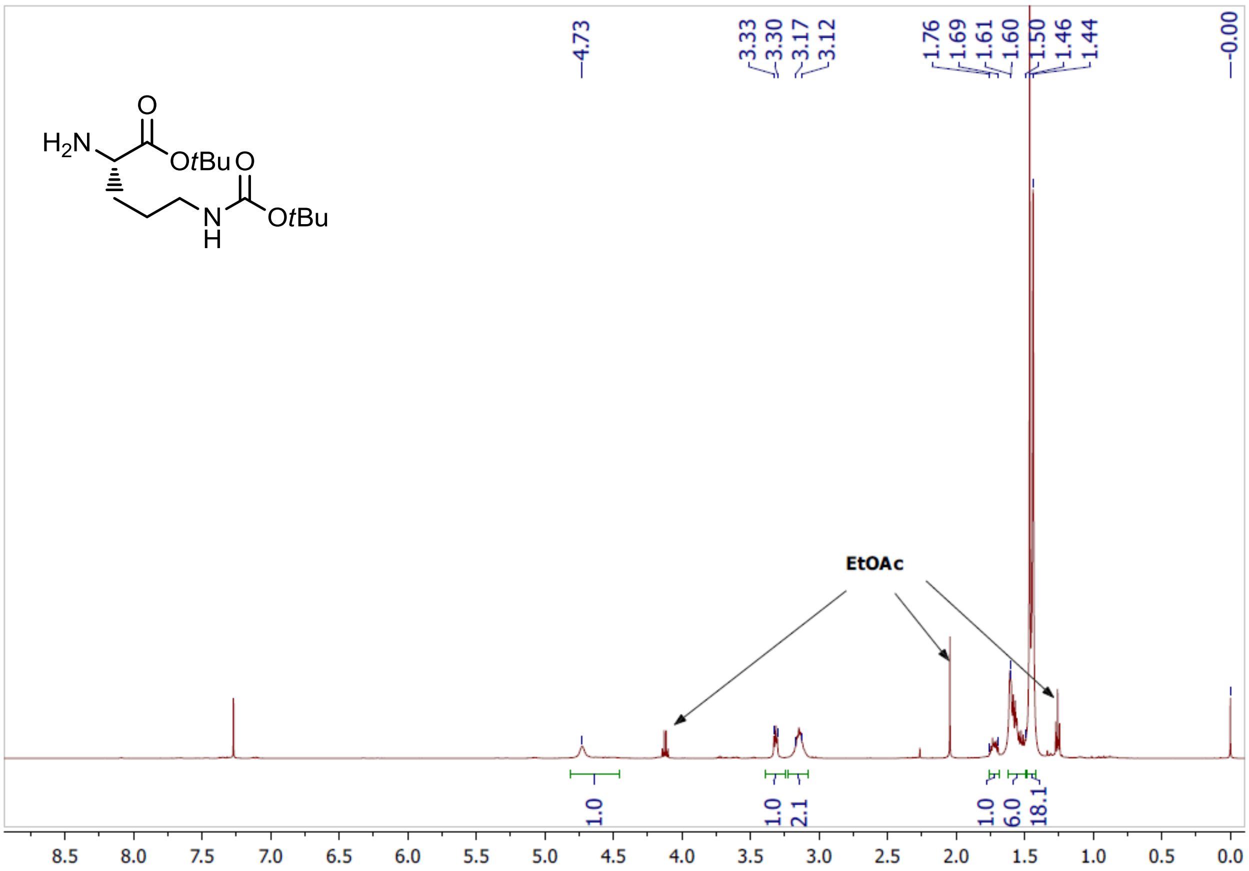

Figure S25. ^{1}H NMR spectrum of compound **9a** (500 MHz, DMSO-d_6)

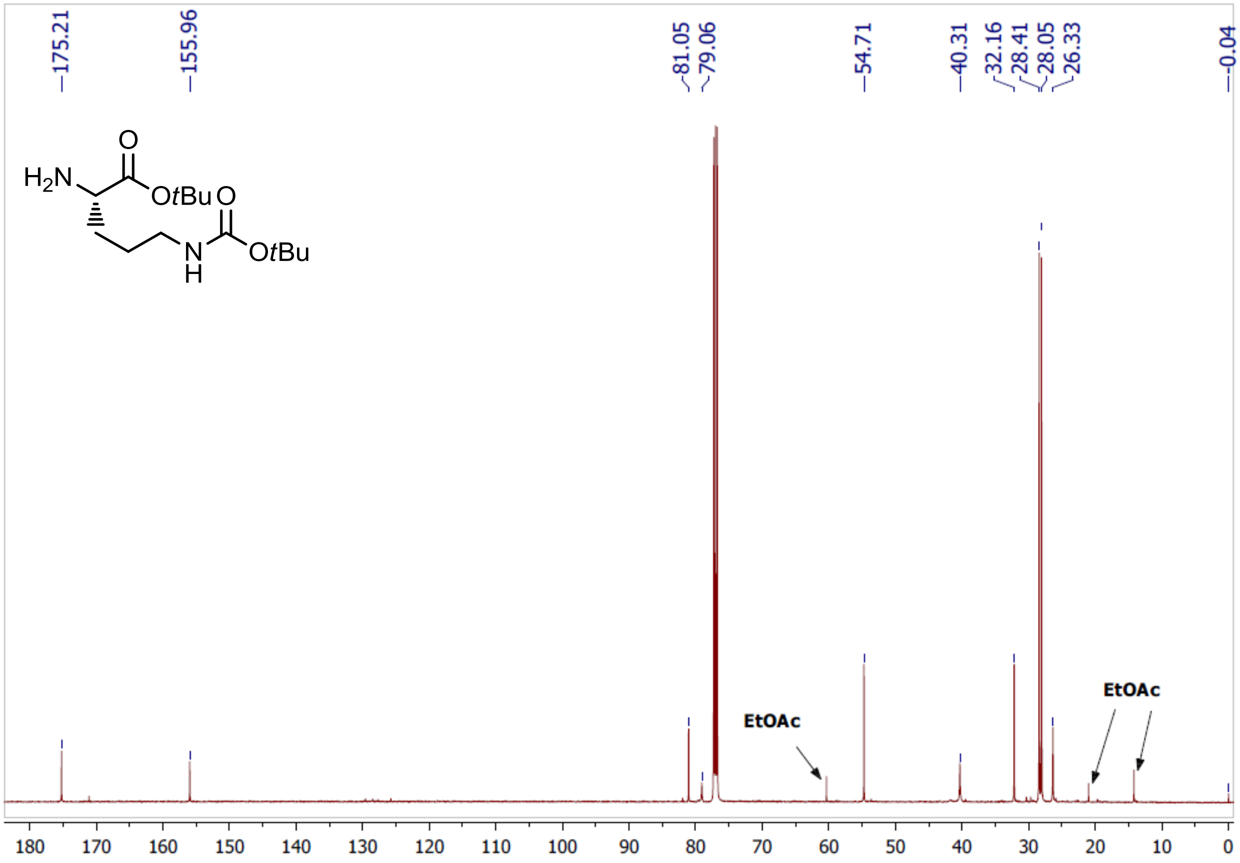

Figure S26. ^{13}C NMR spectrum of compound **9a** (126 MHz, DMSO-d_6)

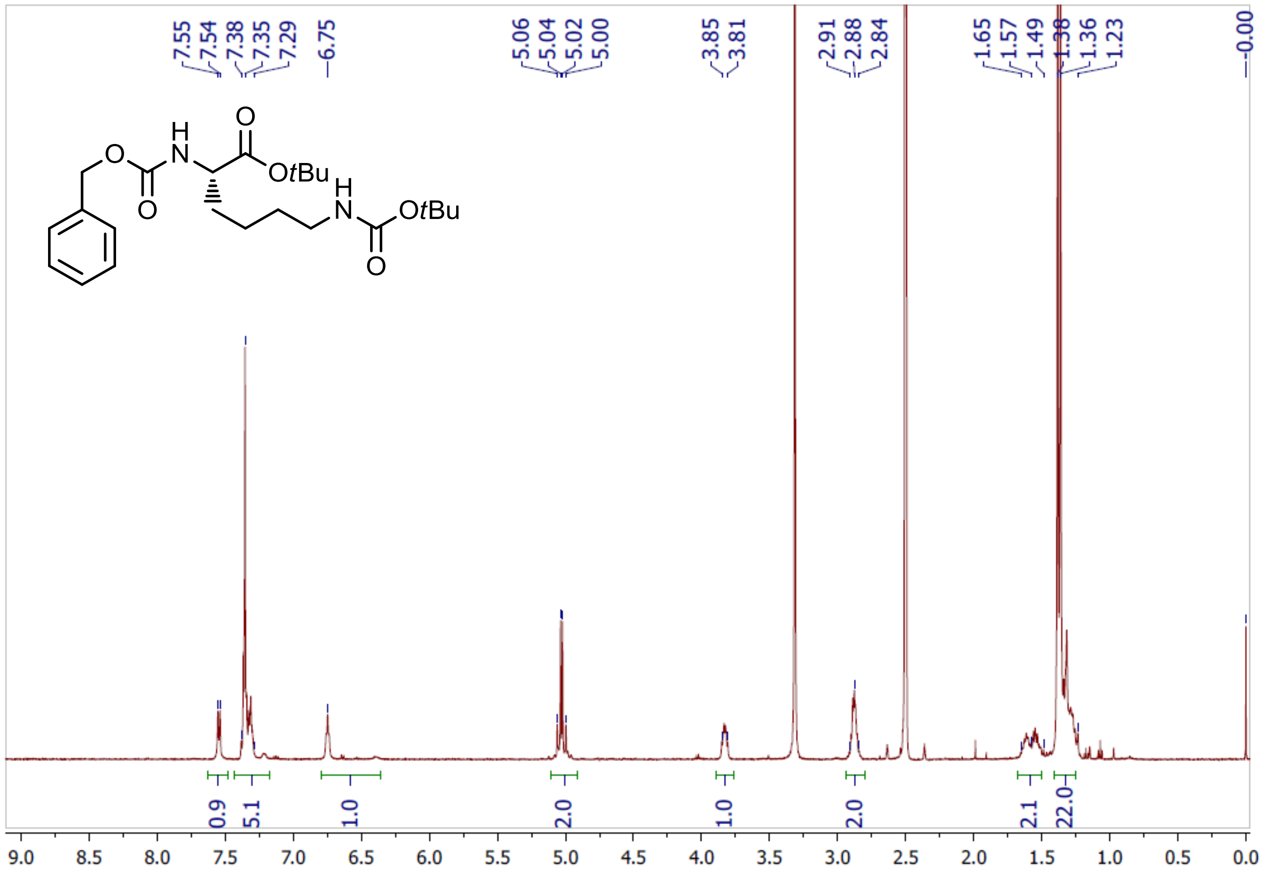

Figure S27. ^{1}H NMR spectrum of compound **11** (500 MHz, DMSO-d_6)

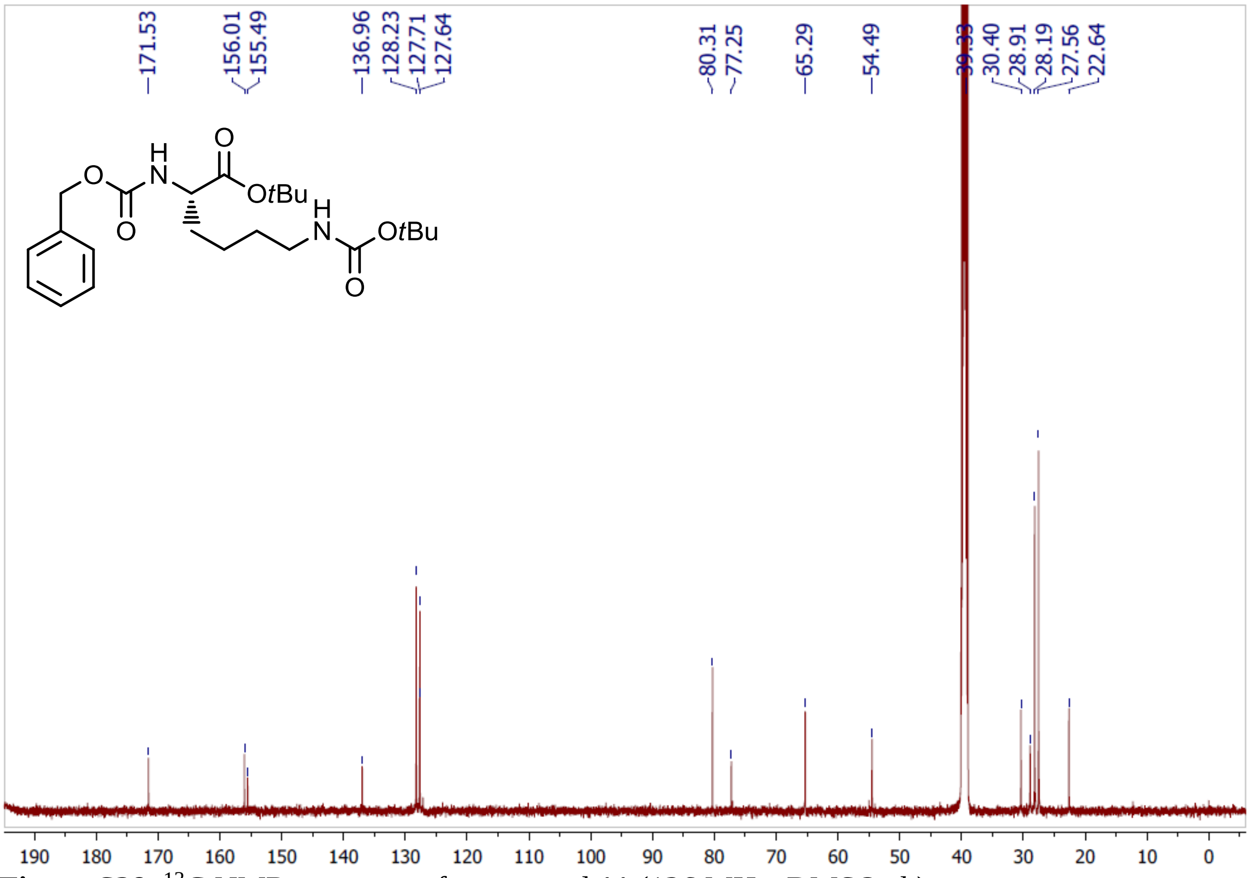

Figure S28. ^{13}C NMR spectrum of compound **11** (126 MHz, DMSO-d_6)

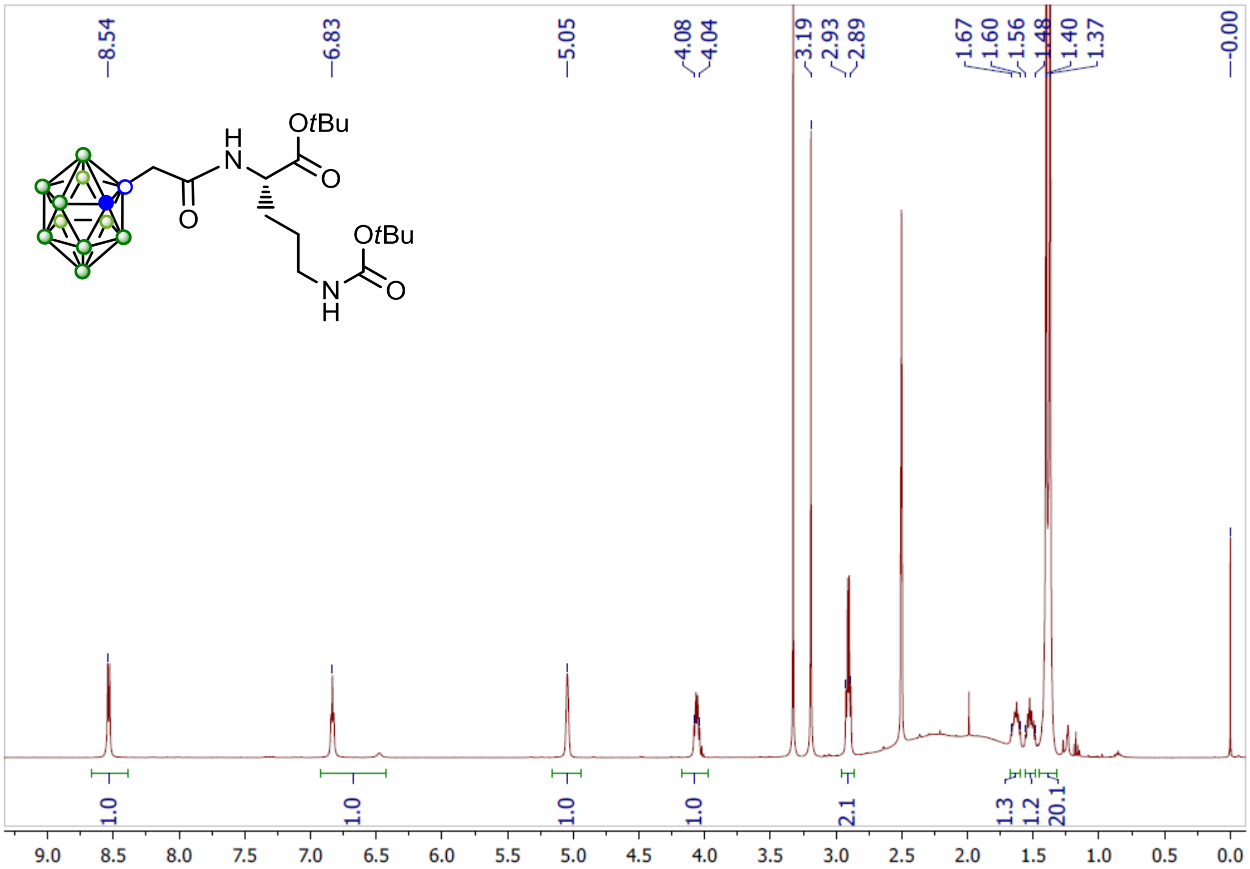

Figure S29. ^{1}H NMR spectrum of compound **12a** (500 MHz, DMSO-d_6)

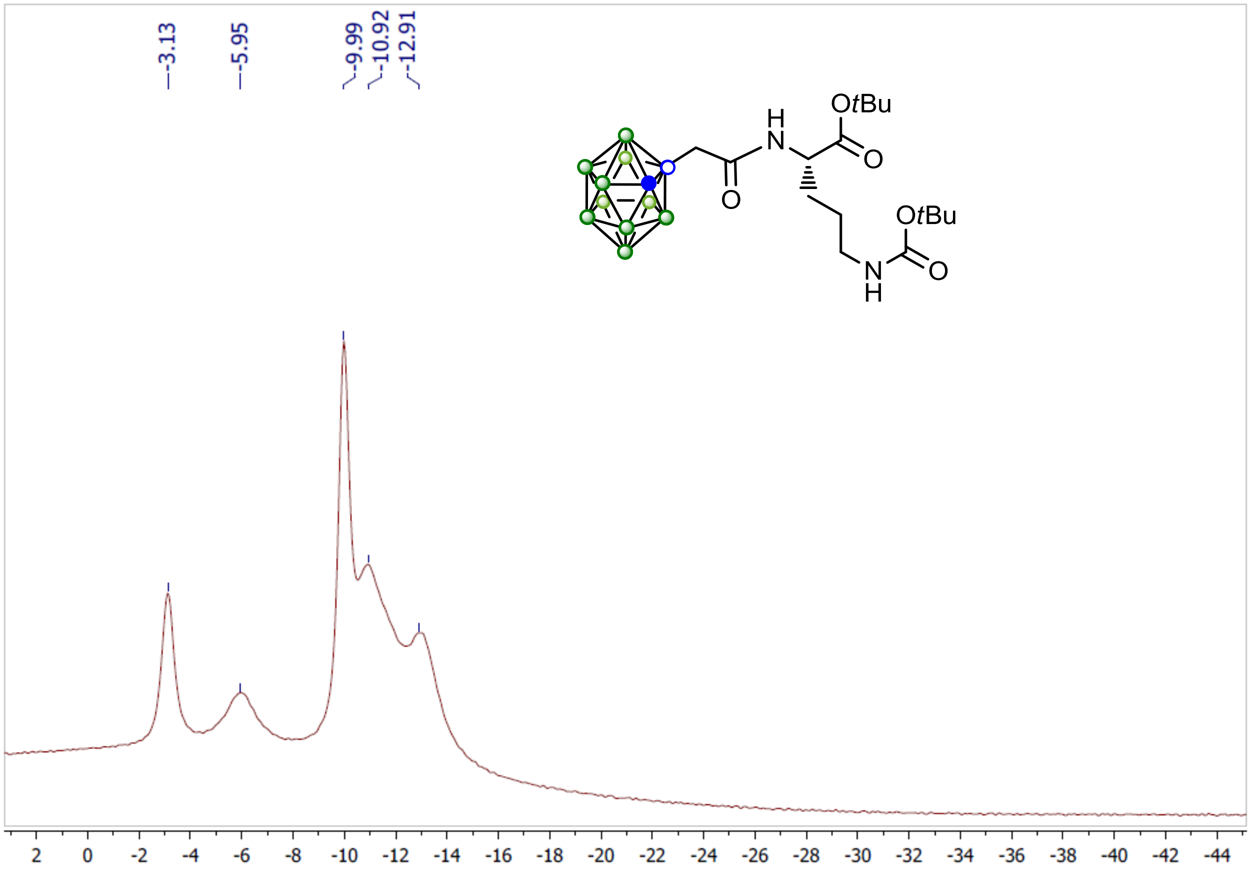

Figure S30. ^{11}B{^{1}H} NMR spectrum of compound **12a** (160 MHz, DMSO-d_6)

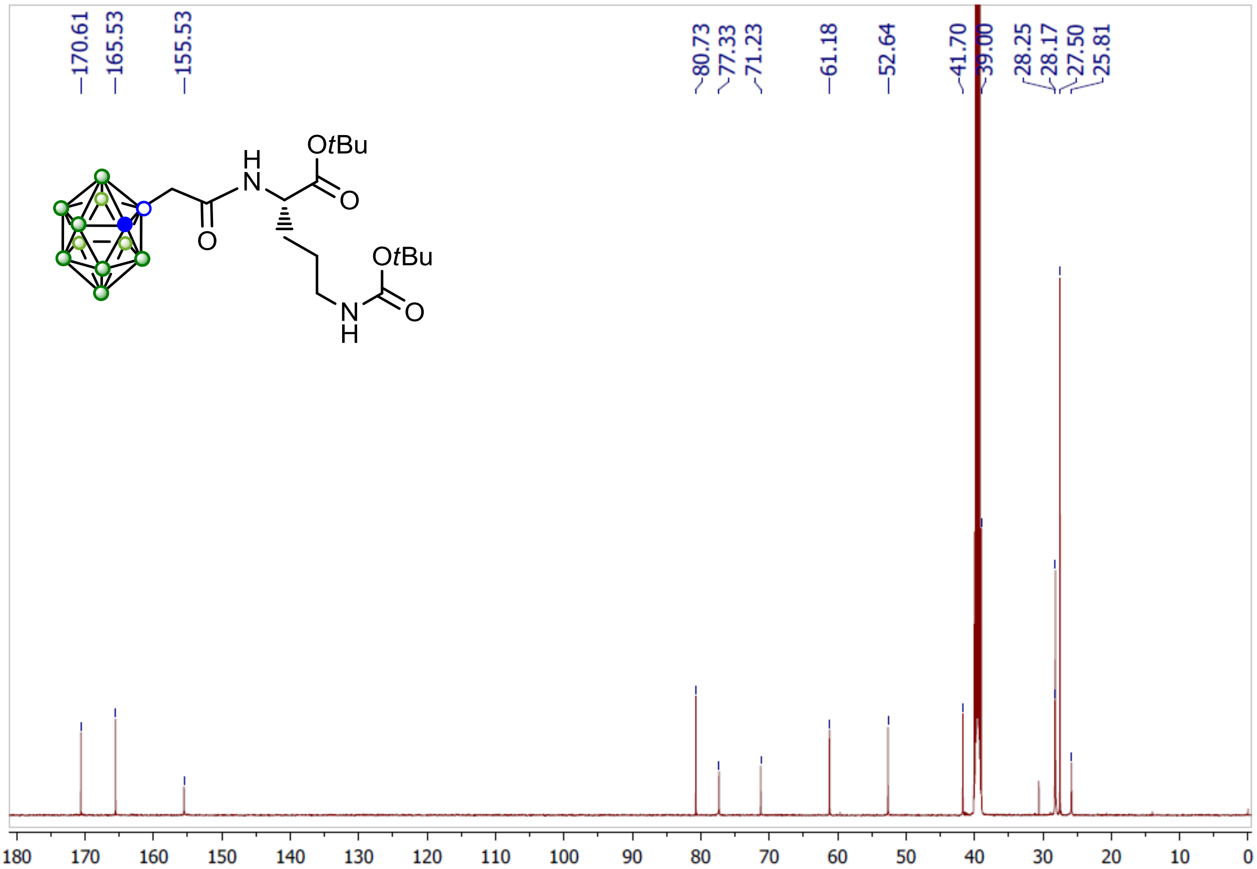

Figure S31. ^{13}C NMR spectrum of compound **12a** (126 MHz, DMSO-d_6)

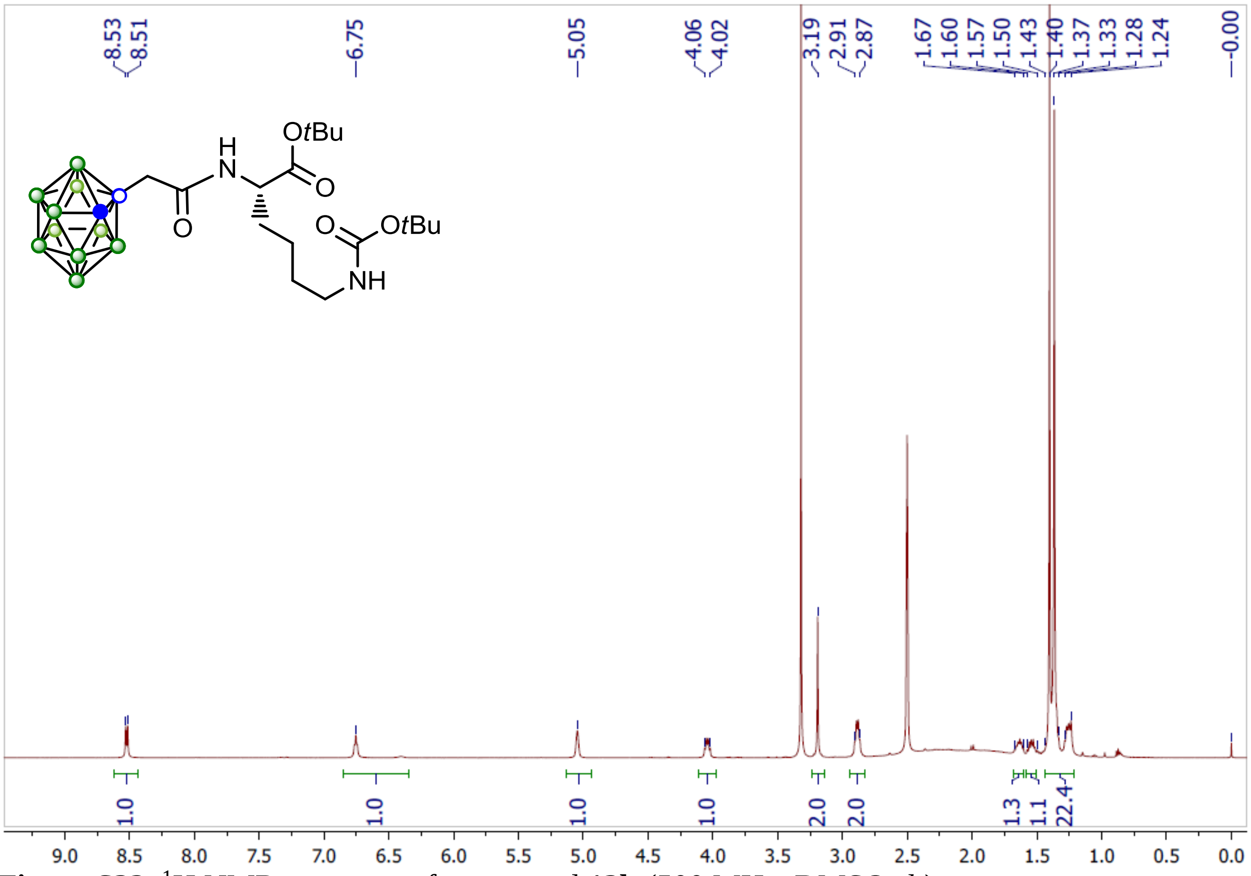

Figure S32. ^{1}H NMR spectrum of compound **12b** (500 MHz, DMSO-d_6)

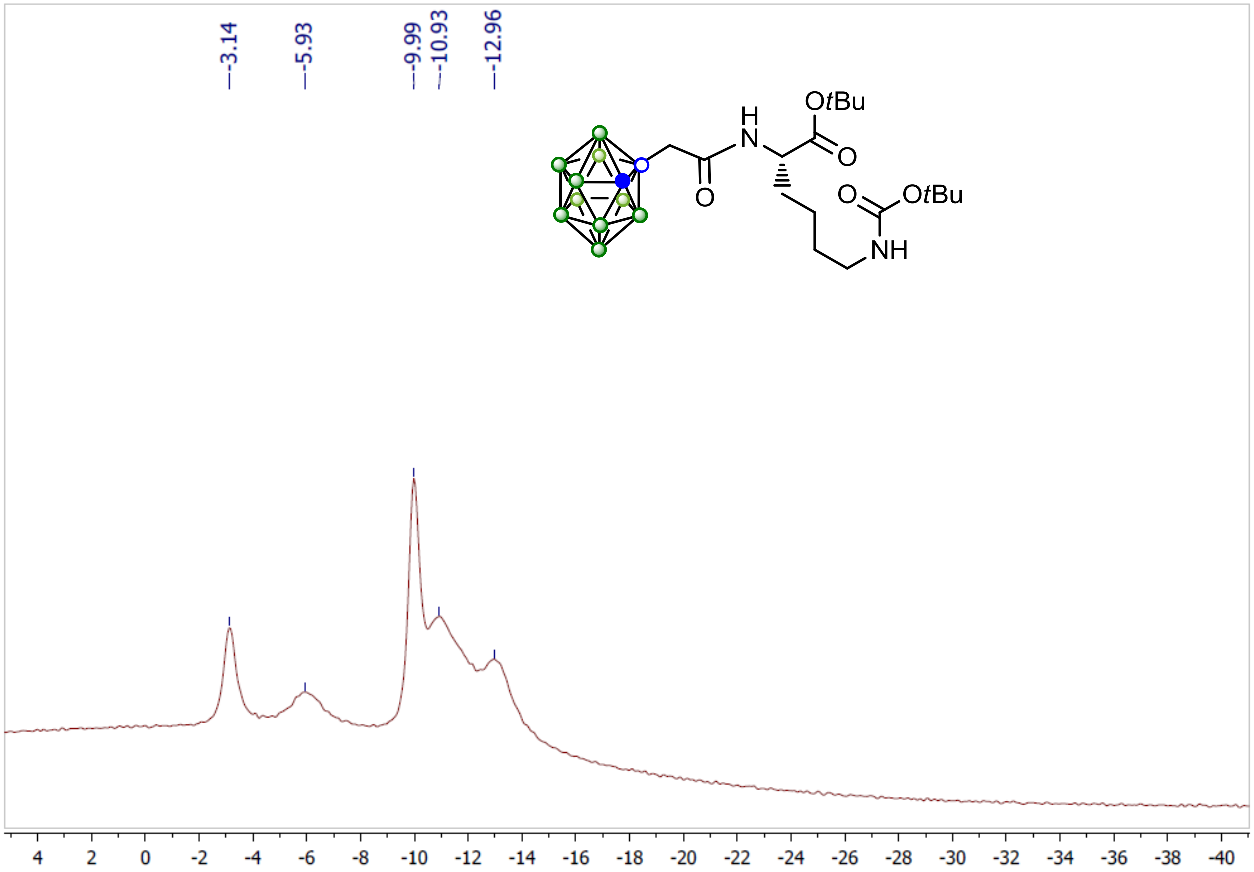

Figure S33. $^{11}B\{^{1}H\}$ NMR spectrum of compound **12b** (160 MHz, DMSO-d_6)

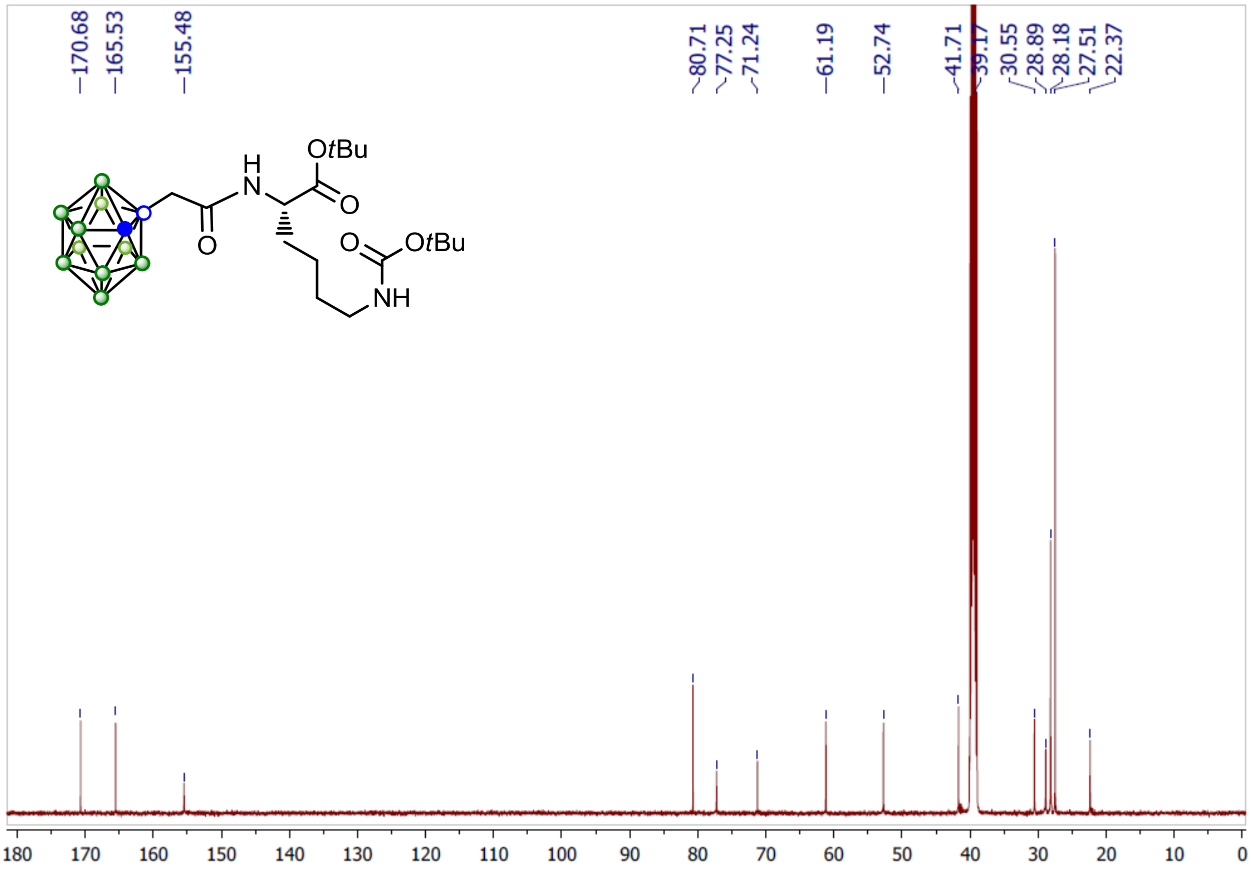

Figure S34. ^{13}C NMR spectrum of compound **12b** (126 MHz, DMSO-d_6)

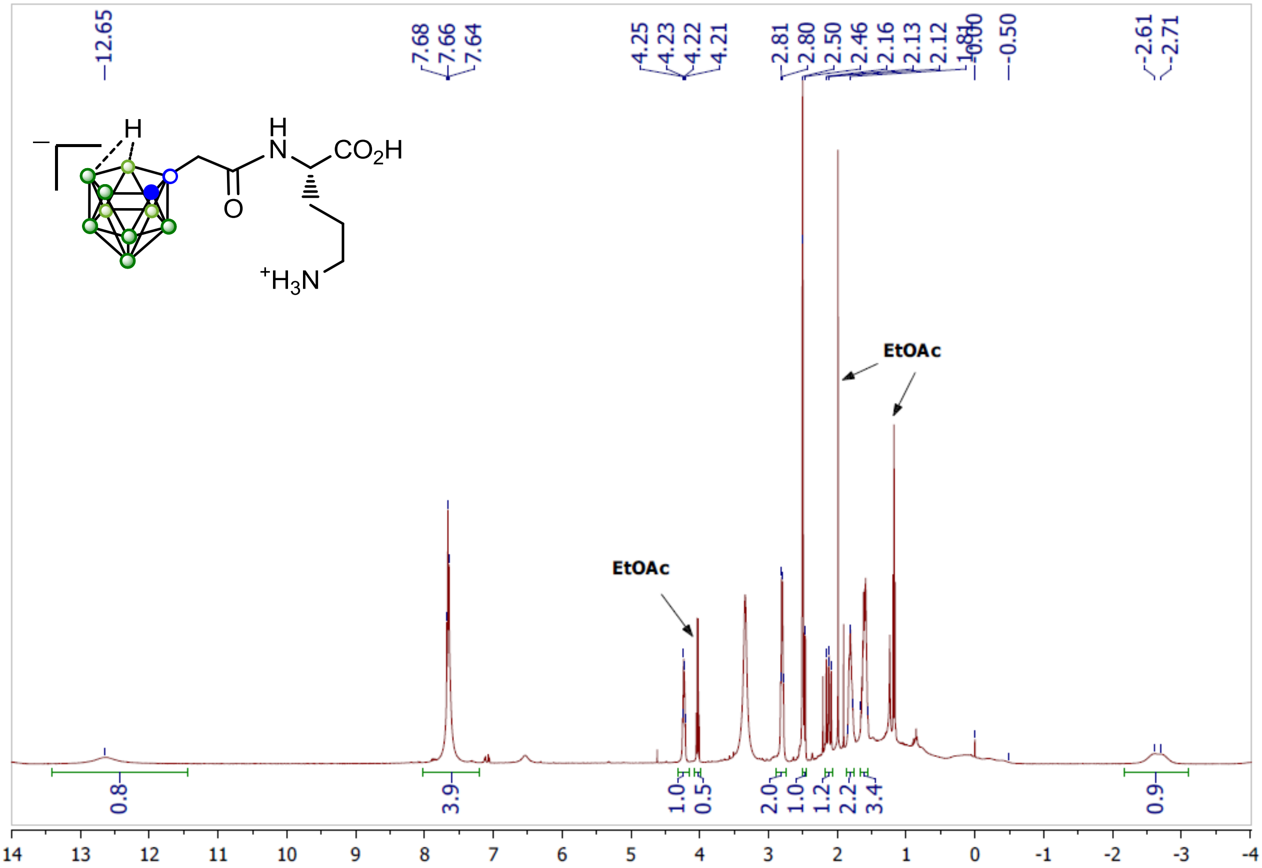

Figure S35. ^{1}H NMR spectrum of compound **13a** (500 MHz, DMSO-d_6)

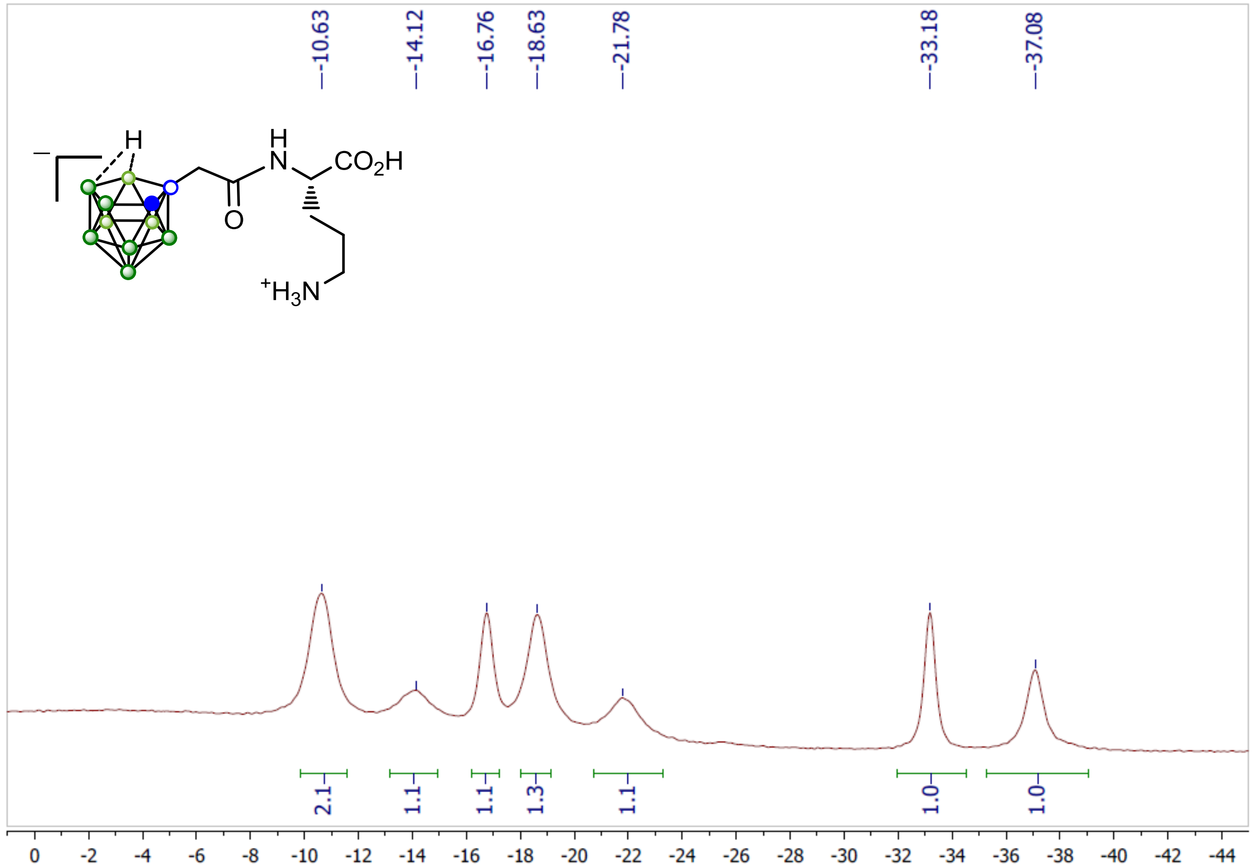
Figure S36. $^{11}B\{^{1}H\}$ NMR spectrum of compound **13a** (160 MHz, DMSO-d_6)

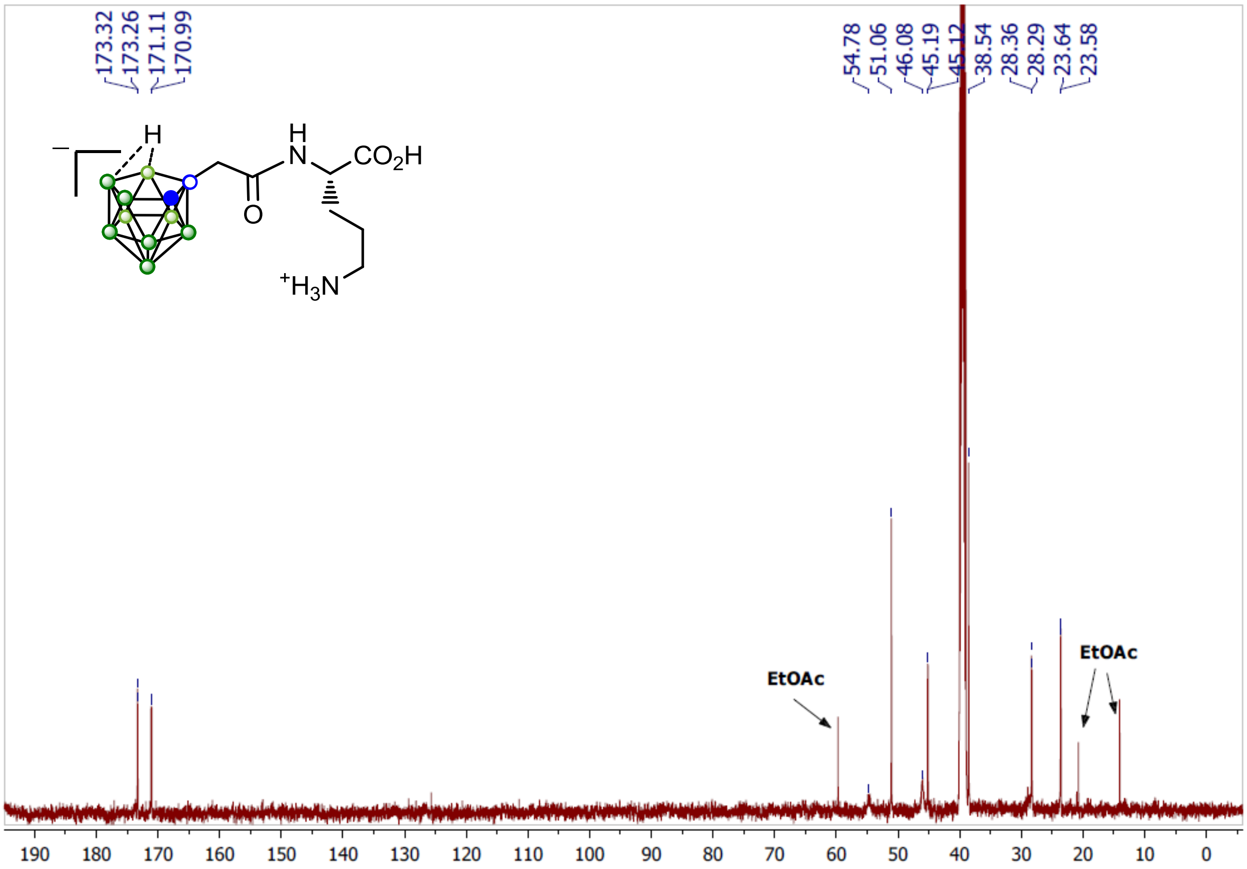

Figure S37. ^{13}C NMR spectrum of compound **13a** (126 MHz, DMSO-d_6)

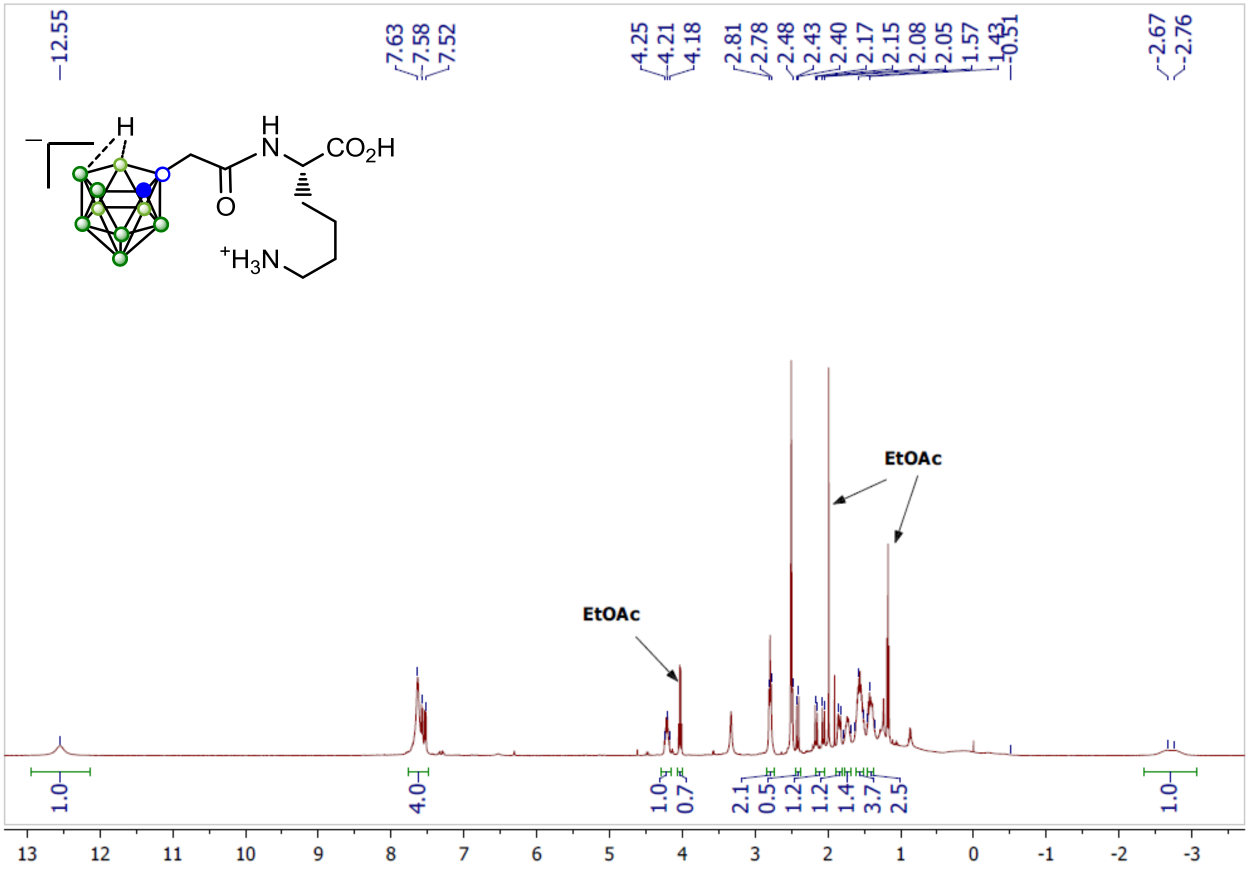

Figure S38. ^{1}H NMR spectrum of compound **13b** (500 MHz, DMSO-d_6)

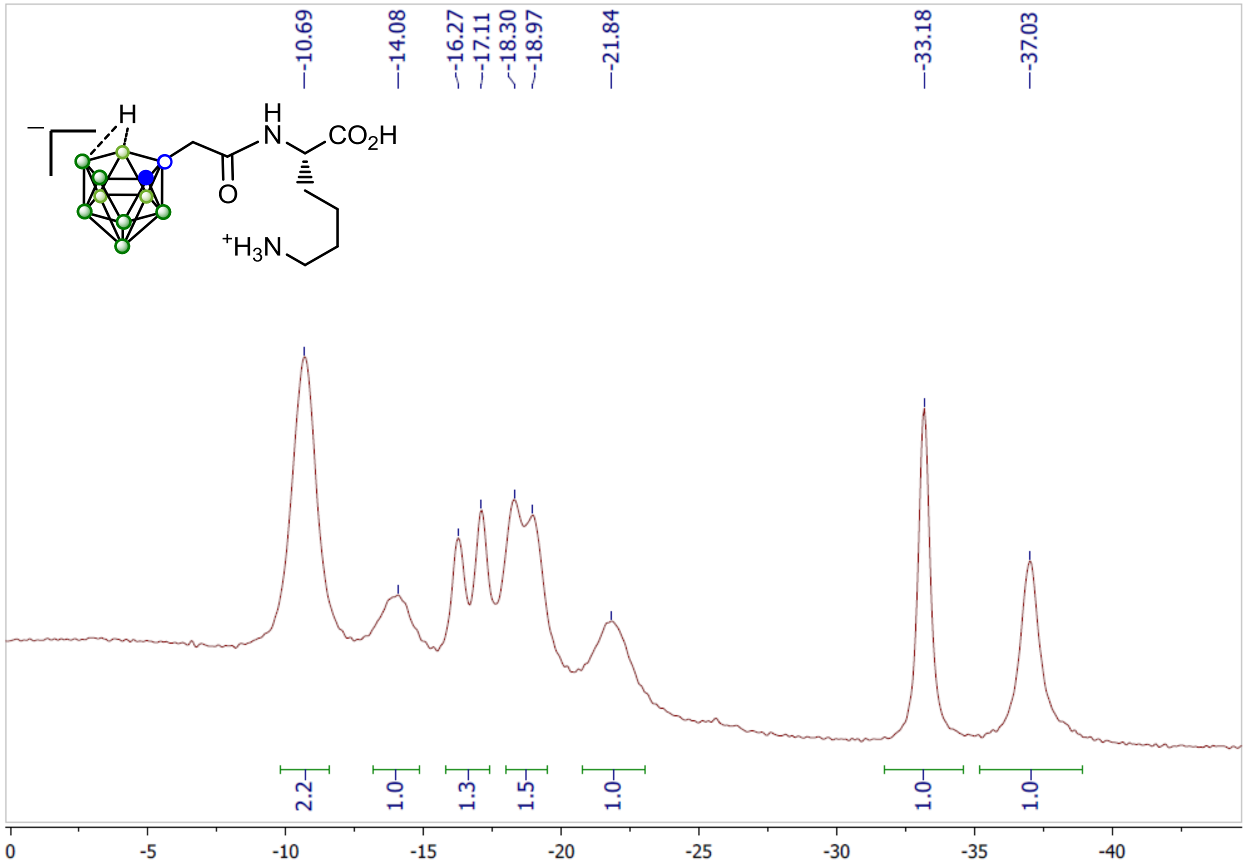

Figure S39. $^{11}B\{^1H\}$ NMR spectrum of compound **13b** (160 MHz, DMSO-d_6)

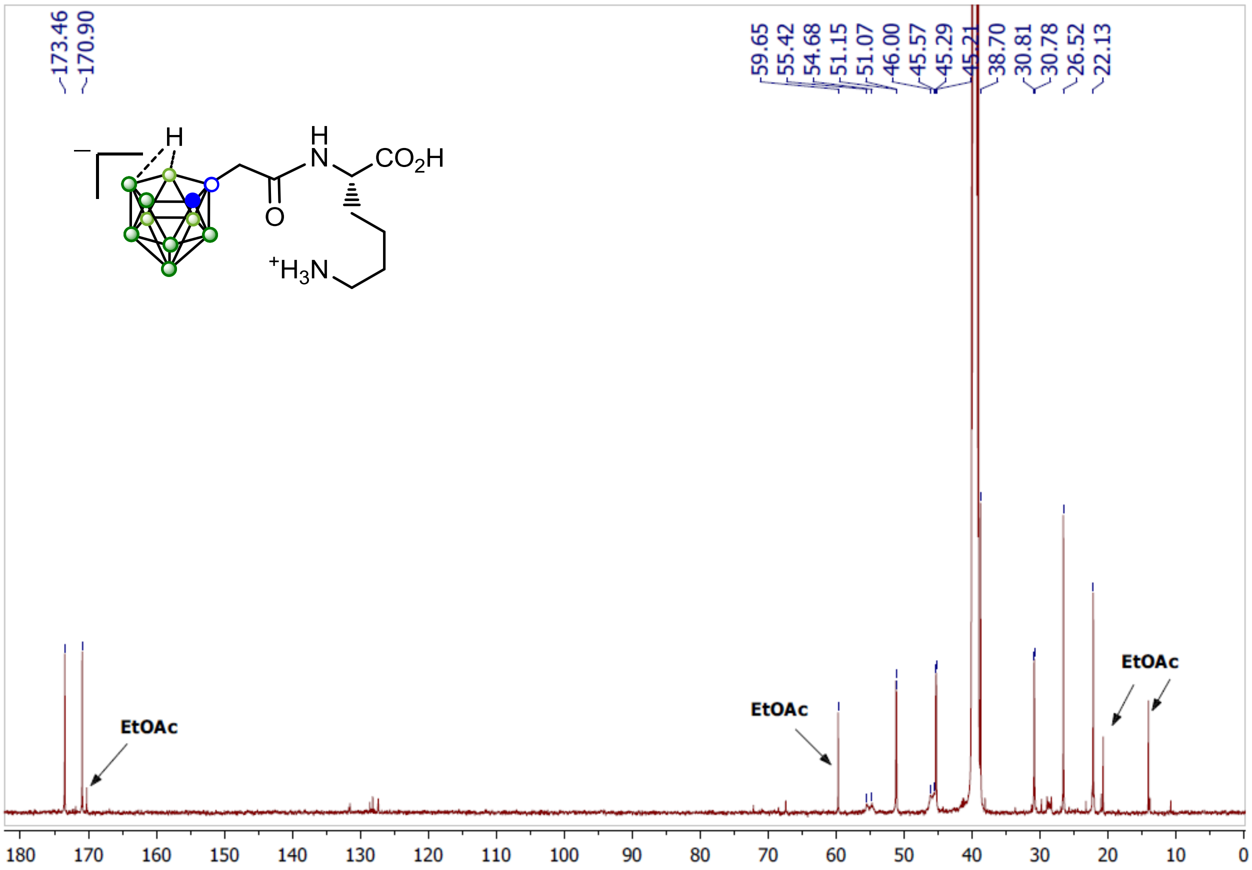

Figure S40. ^{13}C NMR spectrum of compound **13b** (126 MHz, DMSO-d_6)

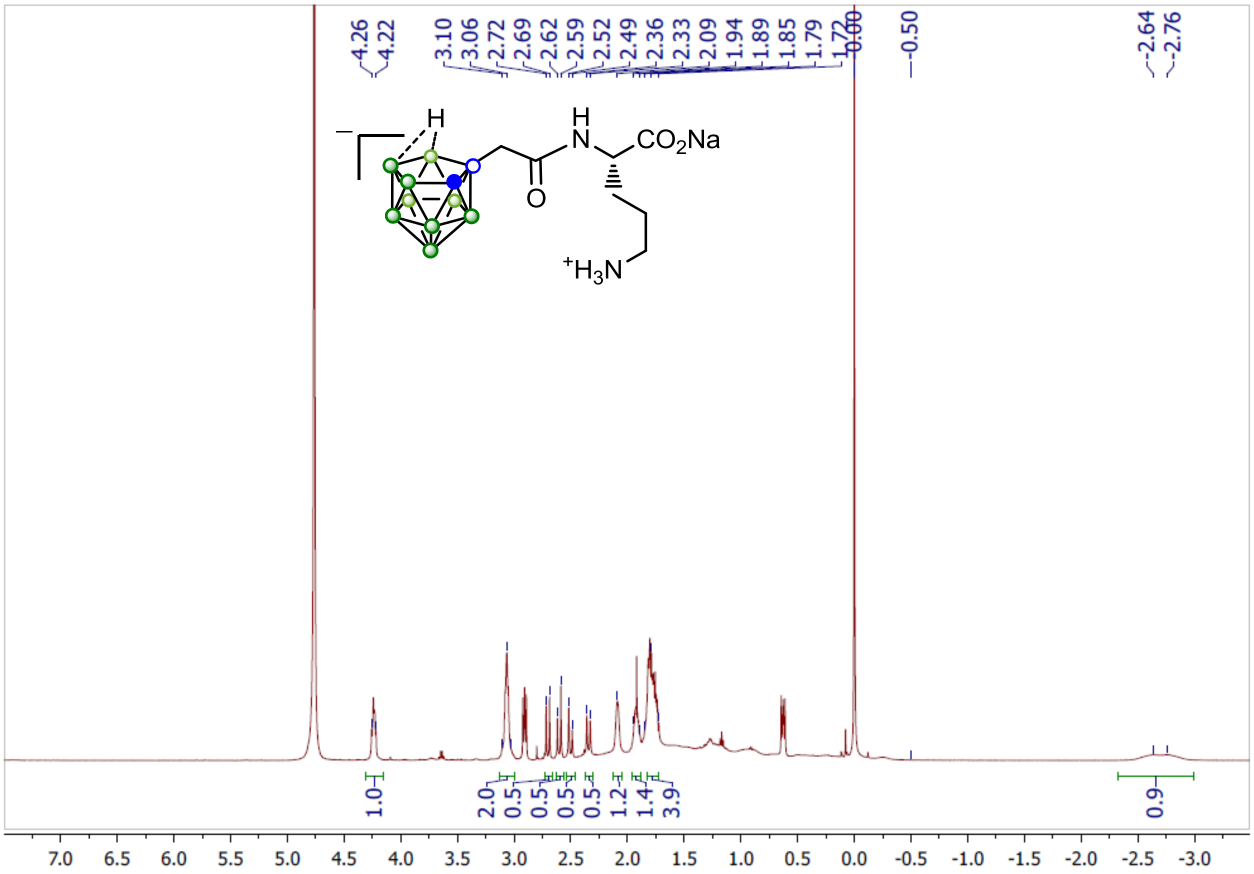

Figure S41. ^{1}H NMR spectrum of compound **14a** (500 MHz, D_2O)

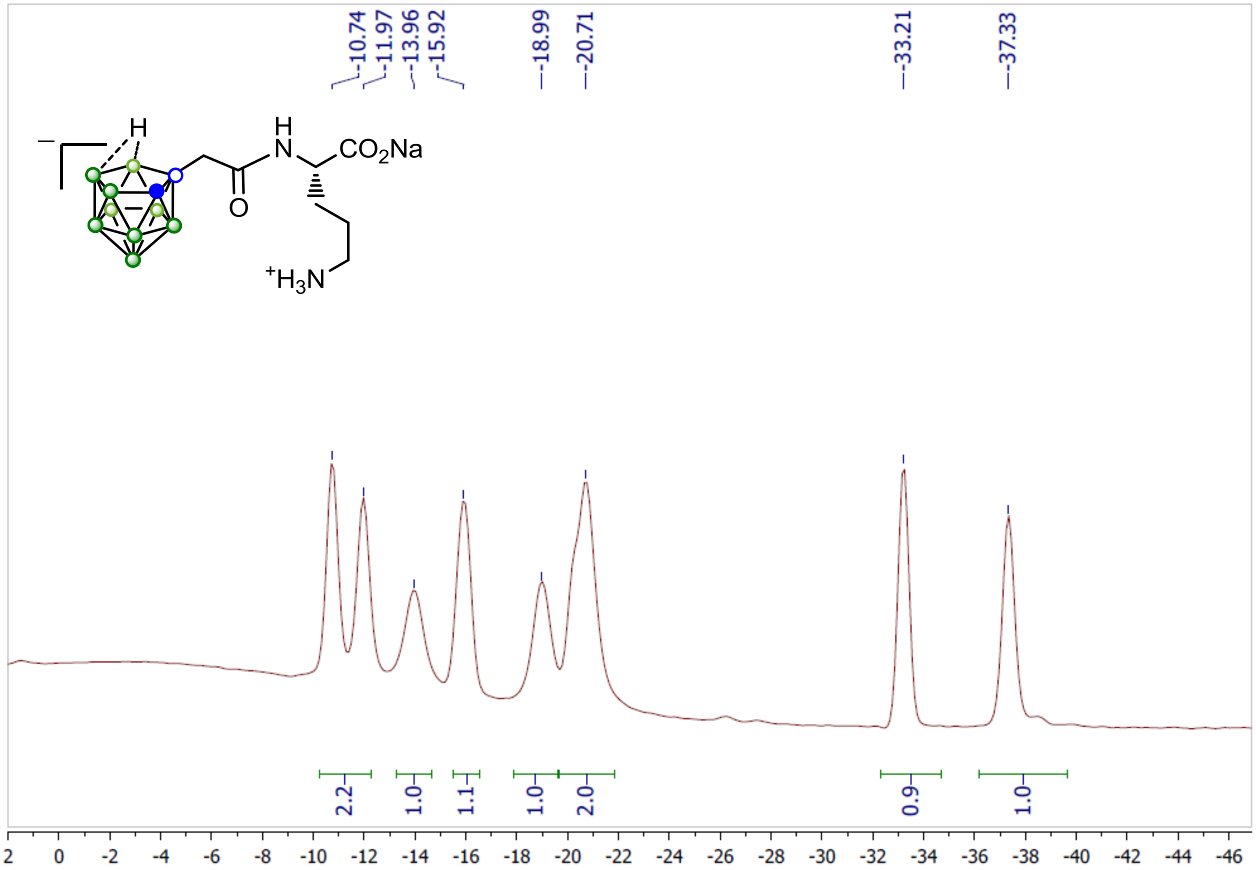

Figure S42. ^{11}B{^{1}H} NMR spectrum of compound **14a** (160 MHz, D_2O)

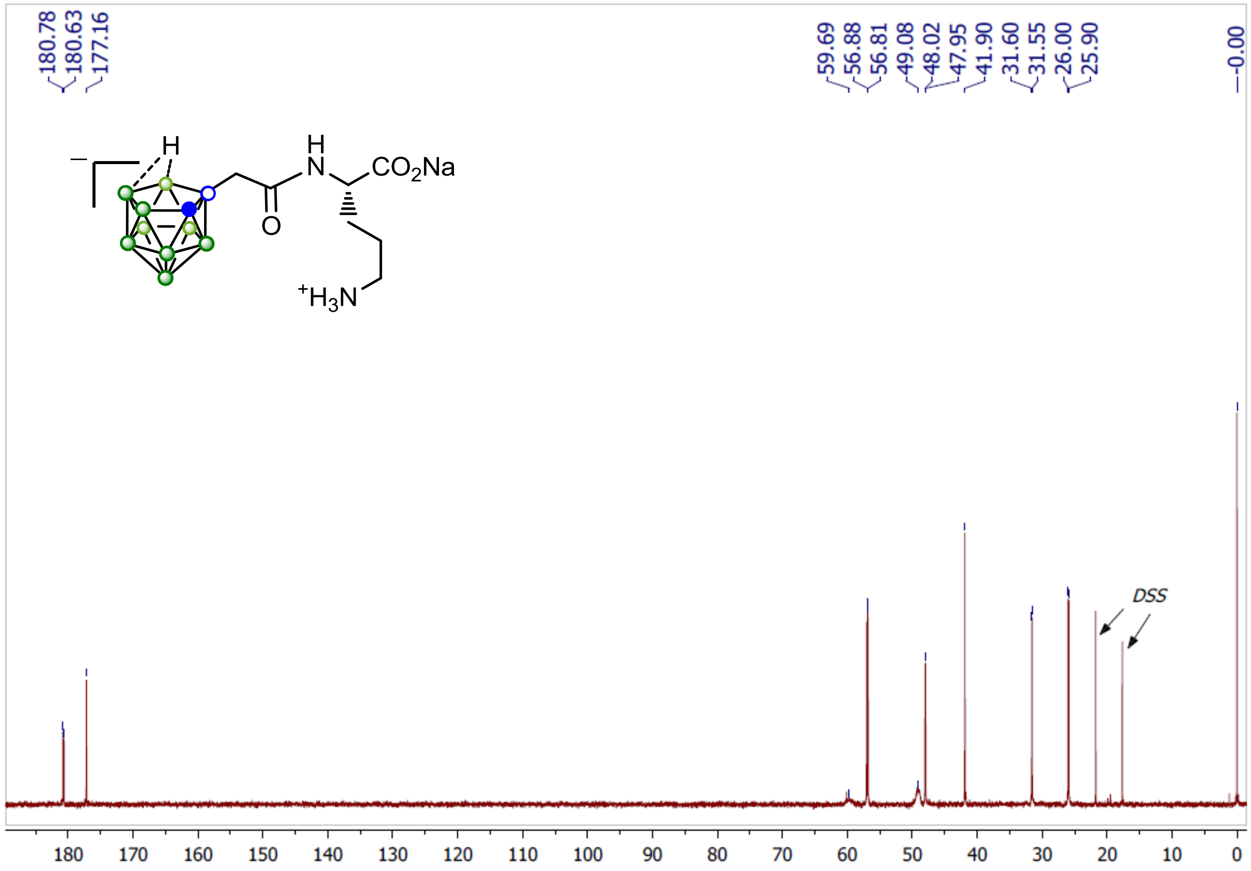

Figure S43. ^{13}C NMR spectrum of compound **14a** (126 MHz, D_2O)

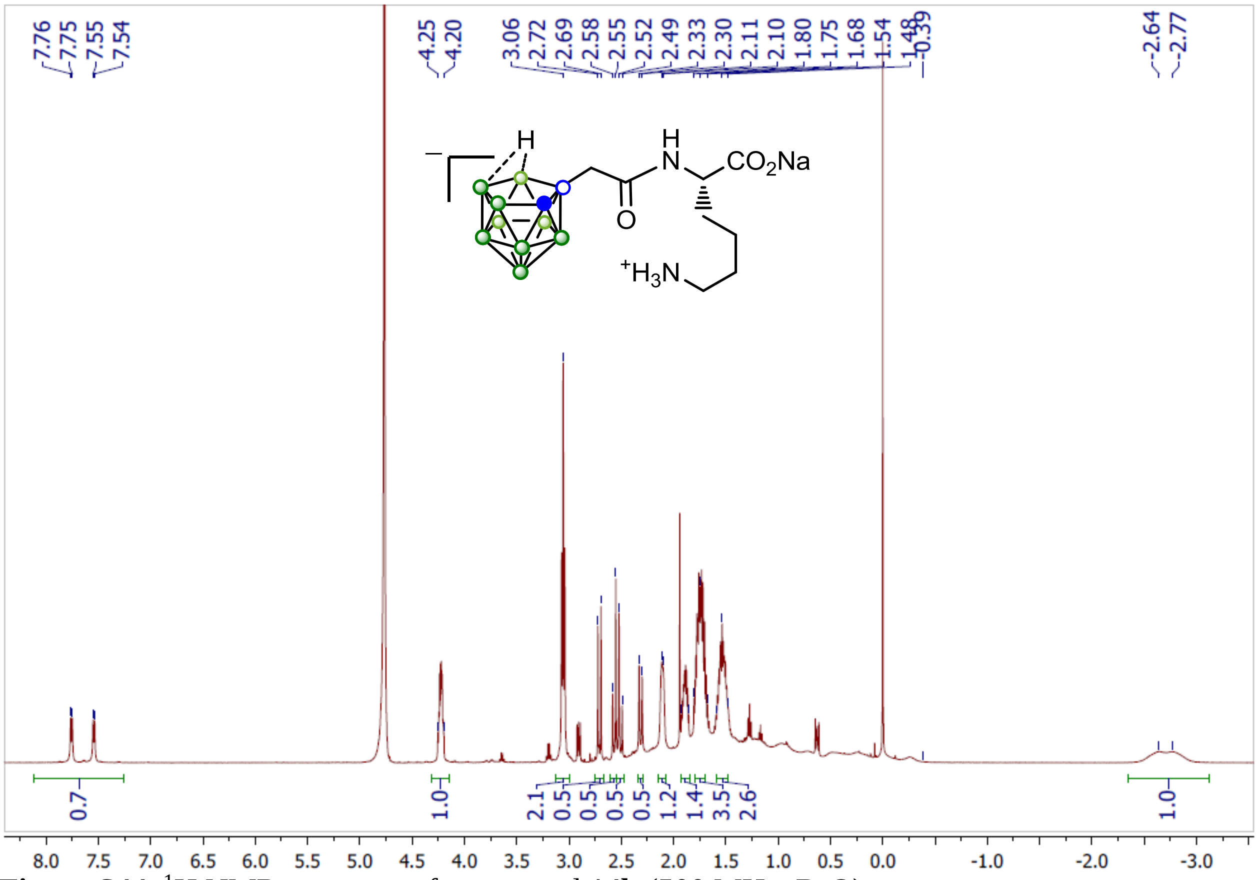

Figure S44. ^{1}H NMR spectrum of compound **14b** (500 MHz, D_2O)

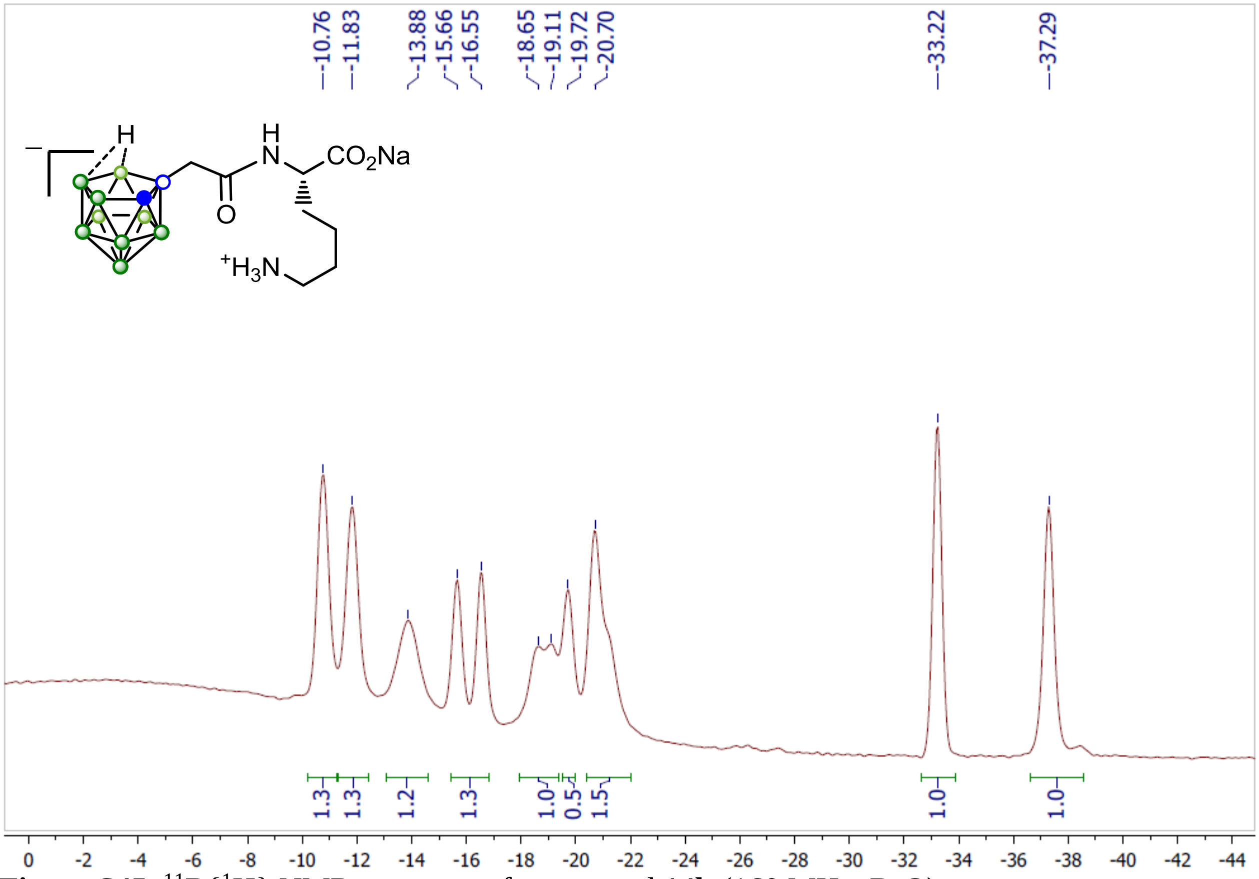

Figure S45. $^{11}B\{^1H\}$ NMR spectrum of compound **14b** (160 MHz, D_2O)

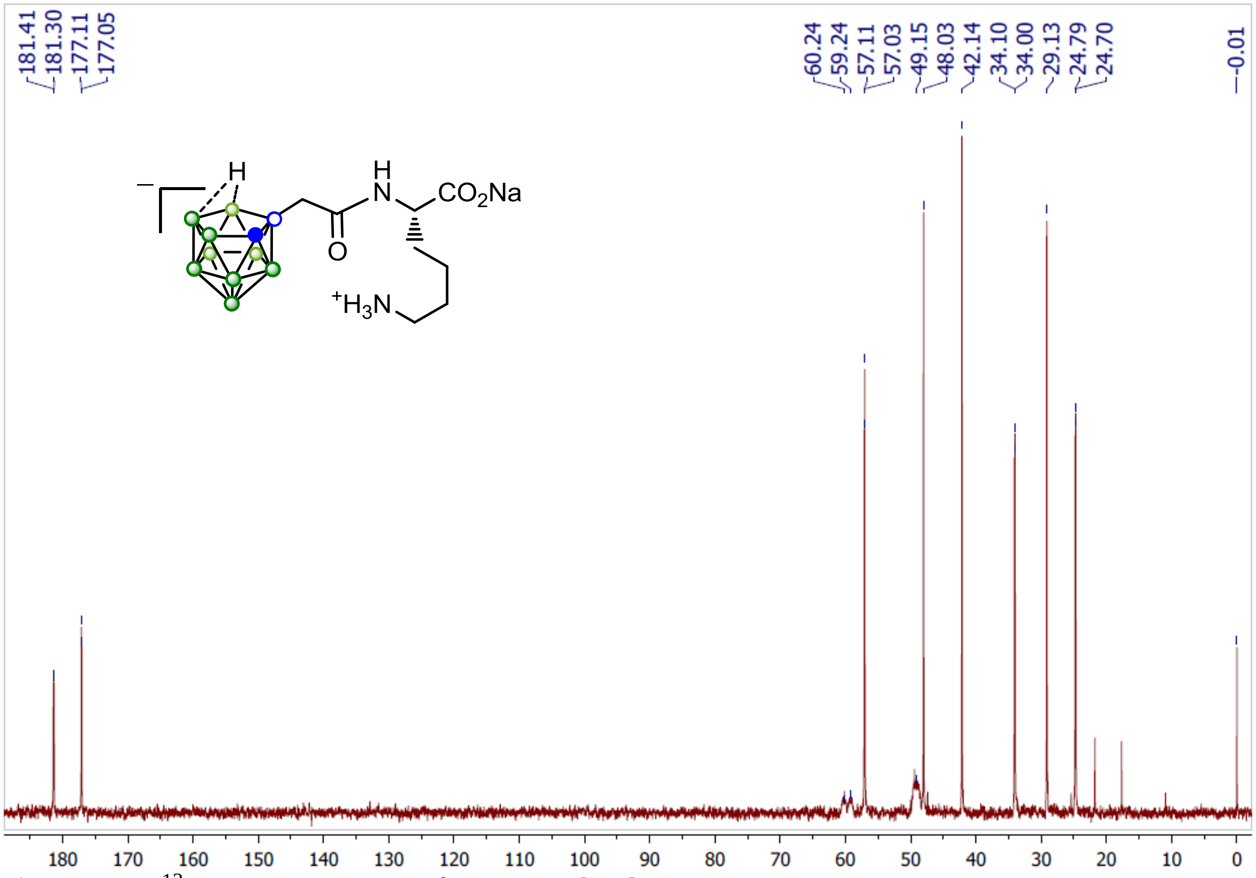

Figure S46. ^{13}C NMR spectrum of compound **14b** (126 MHz, D_2O)

HRMS Data

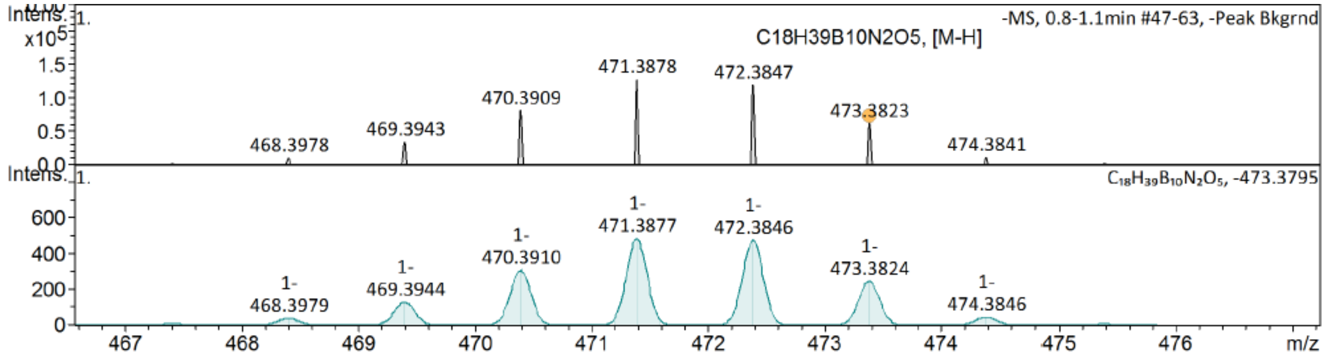

Figure S47. Experimental and simulated peak distribution in high-resolution mass spectrum of compound **3a**

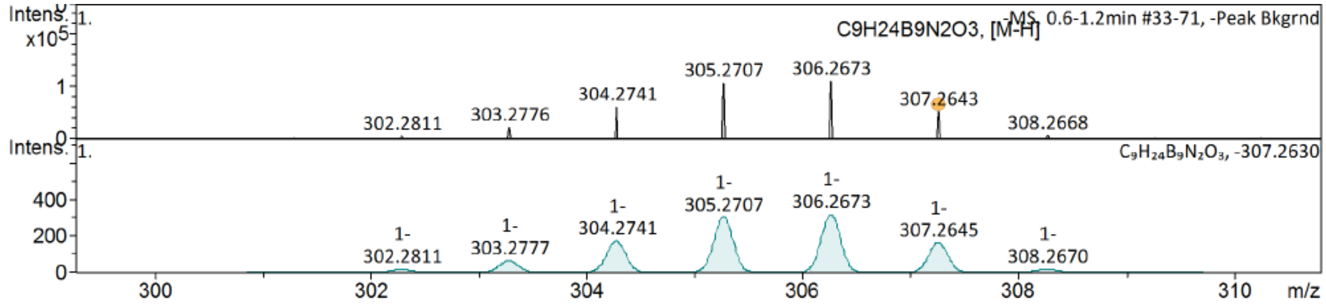

Figure S48. Experimental and simulated peak distribution in high-resolution mass spectrum of compound **4a**

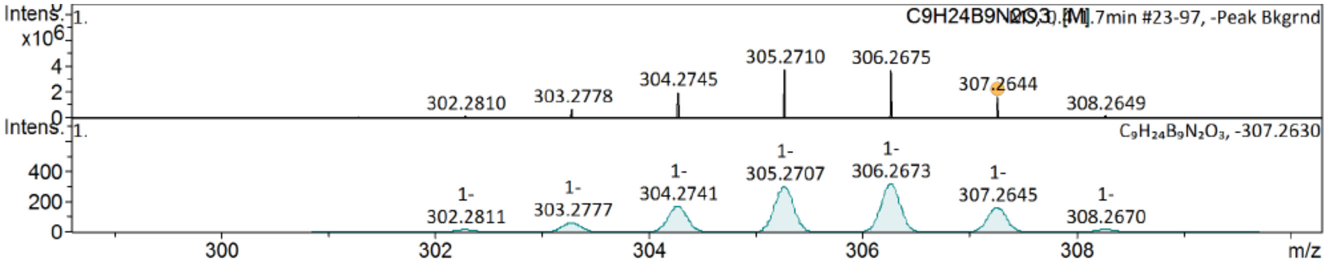

Figure S49. Experimental and simulated peak distribution in high-resolution mass spectrum of compound **5a**

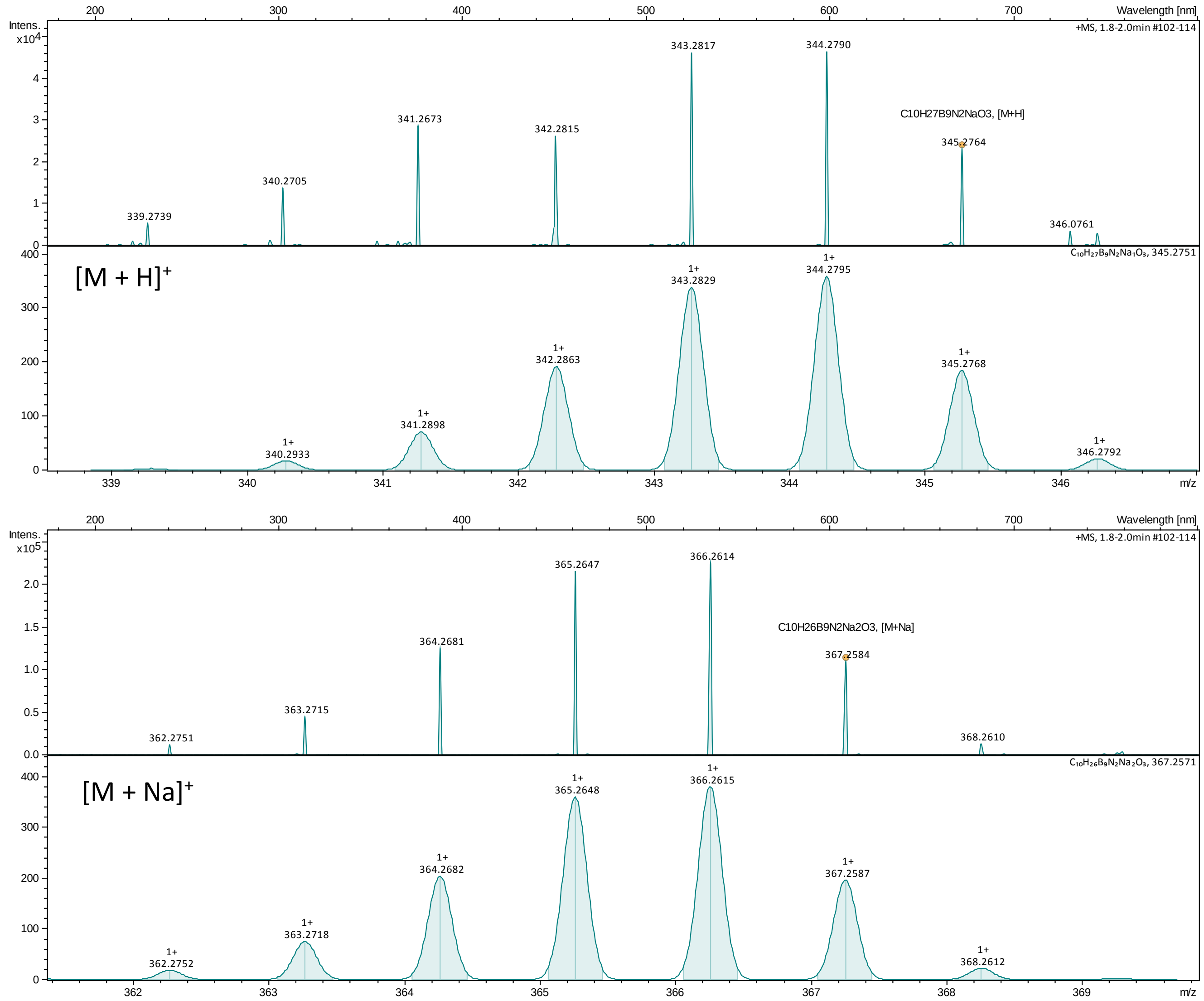

Figure S50. Experimental and simulated peak distribution in high-resolution mass spectra of compound **5b**

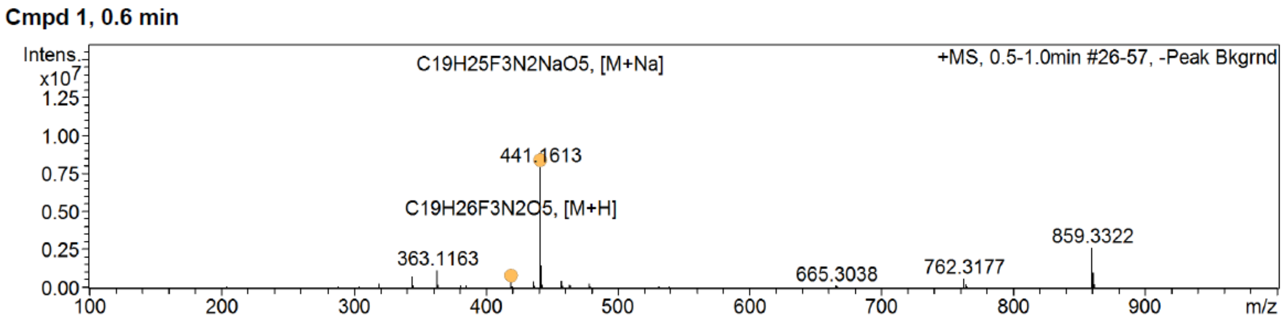

Meas. m/z	#	Ion Formula	m/z	err [ppm]	mSigma	# mSigma	Score	rdb	e¯ Conf	N-Rule
419.1787	1	C19H26F3N2O5	419.1788	0.3	4.4	1	100.00	6.5	even	ok
441.1613	1	C19H25F3N2NaO5	441.1608	-1.2	17.3	12	69.06	6.5	even	ok

Figure S51. Experimental and simulated peak distribution in high-resolution mass spectrum of compound **7**

+MS, 0.5-0.5min #28-29

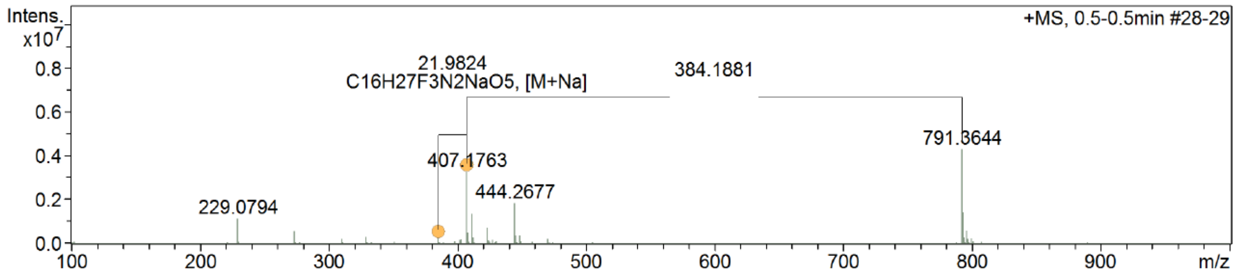

Meas. m/z	#	Ion Formula	m/z	err [ppm]	mSigma	# mSigma	Score	rdb	e¯ Conf	N-Rule
385.1939	1	C16H28F3N2O5	385.1945	1.5	5.5	1	100.00	2.5	even	ok
407.1763	1	C16H27F3N2NaO5	407.1764	0.3	15.9	1	100.00	2.5	even	ok

Figure S52. Experimental and simulated peak distribution in high-resolution mass spectrum of compound **8**

Cmpd 3, 6.6 min

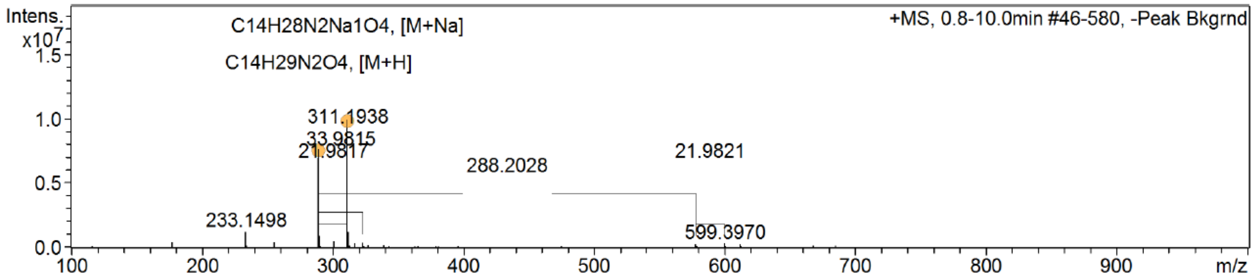

Meas. m/z	#	Ion Formula	m/z	err [ppm]	mSigma	# mSigma	Score	rdb	e¯ Conf	N-Rule
289.2121	1	C14H29N2O4	289.2122	0.2	17.0	1	100.00	1.5	even	ok
311.1938	1	C14H28N2NaO4	311.1941	1.0	17.1	1	100.00	1.5	even	ok

Figure S53. Experimental and simulated peak distribution in high-resolution mass spectrum of compound **9a**

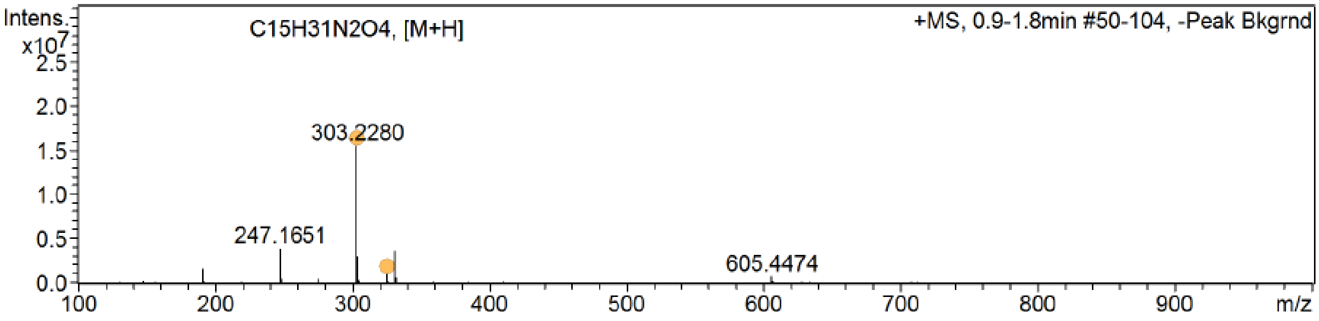

Meas. m/z	#	Ion Formula	m/z	err [ppm]	mSigma	# mSigma	Score	rdb	e¯ Conf	N-Rule
303.2280	1	C15H31N2O4	303.2278	-0.7	15.8	1	100.00	1.5	even	ok
325.2095	1	C15H30N2NaO4	325.2098	0.9	4.8	1	100.00	1.5	even	ok
	1	C15H30N2NaO4	325.2098	0.9	4.8	1	100.00	1.5	even	ok

Figure S54. Experimental and simulated peak distribution in high-resolution mass spectrum of compound **9b**

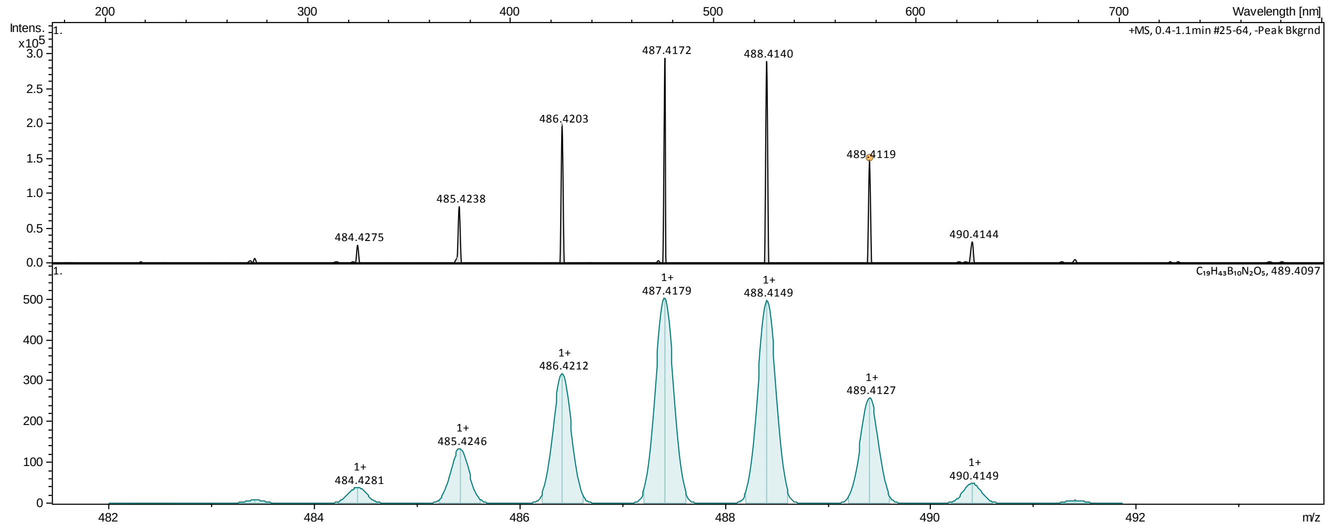

Figure S55. Experimental and simulated peak distribution in high-resolution mass spectrum of compound **12b**

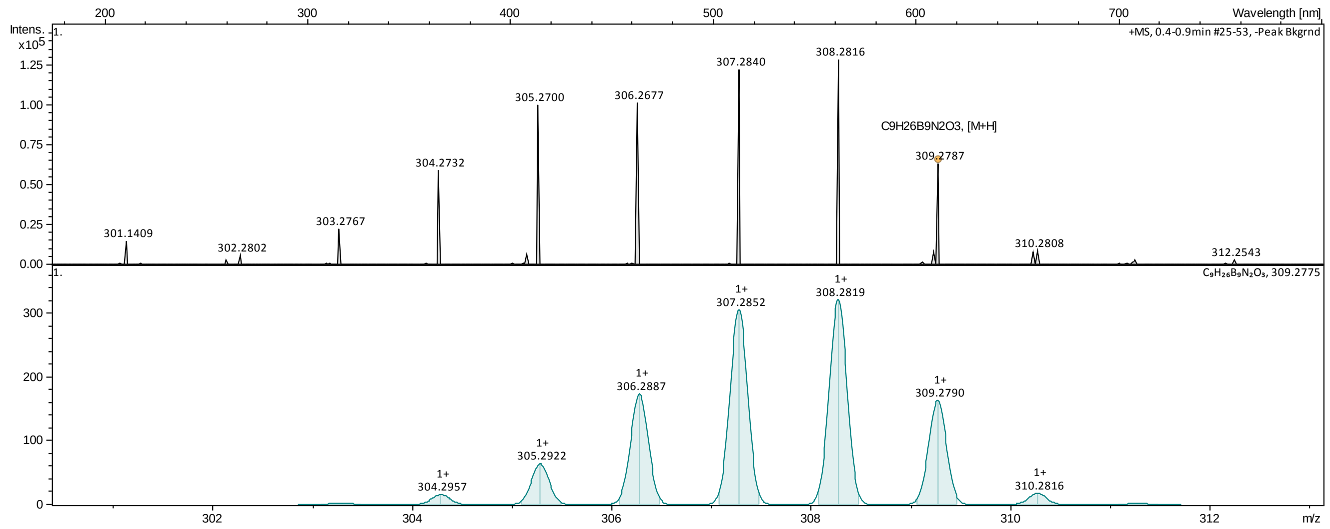

Figure S56. Experimental and simulated peak distribution in high-resolution mass spectrum of compound **13a**

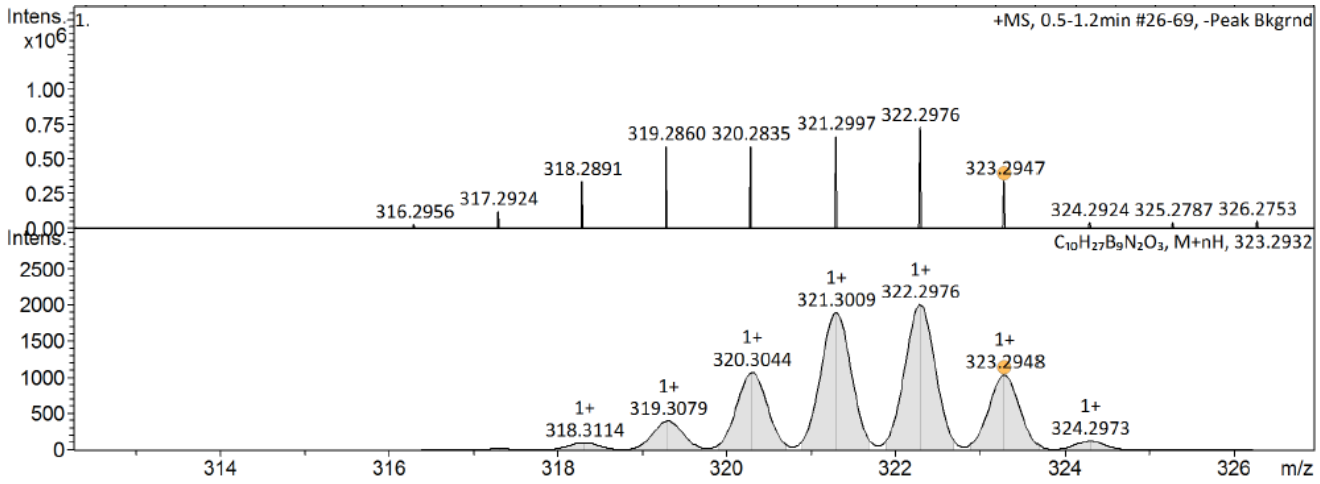

Figure S57. Experimental and simulated peak distribution in high-resolution mass spectrum of compound **13b**

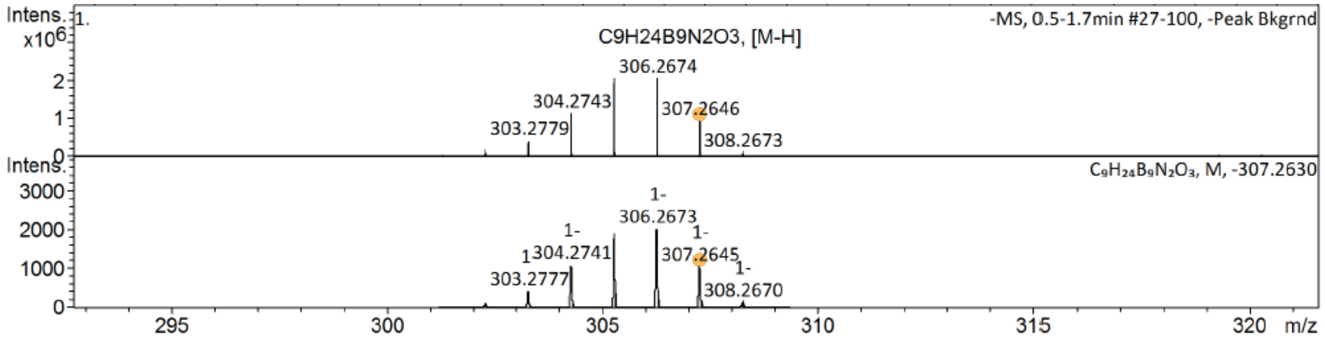

Figure S58. Experimental and simulated peak distribution in high-resolution mass spectrum of compound **14a**

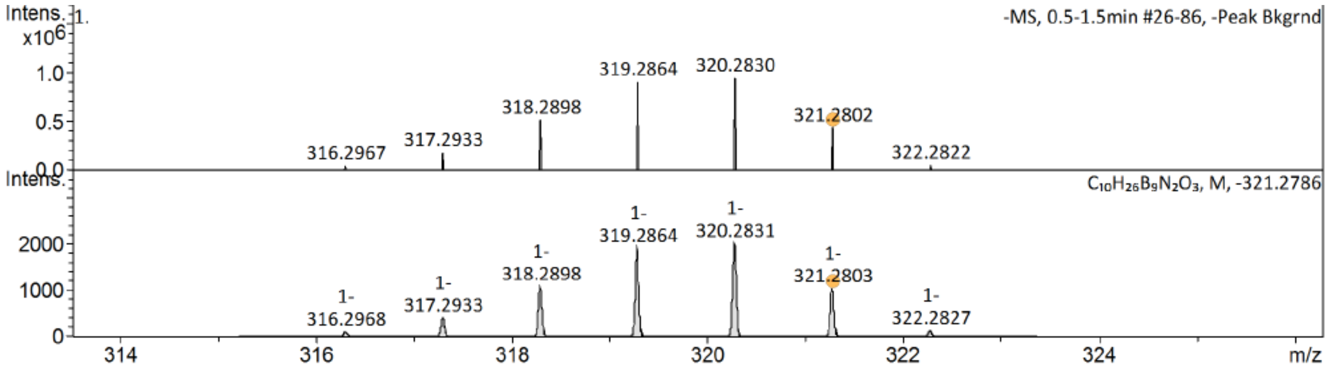

Figure S59. Experimental and simulated peak distribution in high-resolution mass spectrum of compound **14b**

Table S1. Cell Viability after 72-h Co-incubation with Compounds **5a** and **5b** and Cisplatin as a Positive Control [a]

Cell line	Compound	Concentration (mg/mL)	Cell viability (%)	SE (%)[b]
BJ-5ta	**5a**	0.008	101.8	0.6
		0.016	96.5	2.6
		0.031	99.5	1.0
		0.063	96.7	0.6
		0.125	95.9	0.5
		0.250	92.3	1.5
		0.500	85.6	3.0
		1.000	38.8	2.4
	**5b**	0.008	101.6	2.0
		0.016	103.5	4.7
		0.031	122.0	5.1
		0.063	107.6	3.0
		0.125	100.6	2.5
		0.250	95.9	3.6
		0.500	93.8	2.3
		1.000	71.9	3.4
	**Cisplatin**	0.0008	87.5	1.6
		0.0016	86.9	4.5
		0.0031	82.3	2.0
		0.0063	79.7	1.9
		0.0125	71.3	3.2
		0.0250	44.9	3.7
		0.0500	28.5	2.6
		0.1000	18.5	0.2
DU-145	**5a**	0.008	103.2	1.0
		0.016	101.7	1.3
		0.031	101.5	1.5
		0.063	100.4	1.5
		0.125	99.9	1.1
		0.250	88.4	2.3
		0.500	58.2	2.9
		1.000	48.9	1.2
	**5b**	0.008	104.2	4.4
		0.016	93.0	3.9
		0.031	92.9	3.4
		0.063	84.8	5.2
		0.125	91.0	6.8
		0.250	76.1	3.3
		0.500	56.2	3.8
		1.000	14.2	3.0
	**Cisplatin**	0.0008	53.7	6.9
		0.0016	41.4	2.0
		0.0031	30.5	1.0
		0.0063	22.3	1.5
		0.0125	17.1	0.6
		0.0250	16.8	1.7
		0.0500	8.3	0.8
		0.1000	7.5	0.4

[a] Results of three independent experiments are presented.
[b] SE is the standard error calculated according to equation: $SE = {}^{s}/_{\sqrt{3}}$, where *s* is the standard deviation.

Table S1. *Continuation*

Cell line	Compound	Concentration (mg/mL)	Cell viability (%)	SE (%)[b]
MDA-MB-231	**5a**	0.008	100.6	1.5
		0.016	97.6	2.1
		0.031	93.6	2.3
		0.063	92.3	2.9
		0.125	81.0	2.6
		0.250	69.0	1.8
		0.500	59.6	2.0
		1.000	44.9	2.7
	**5b**	0.008	105.0	5.4
		0.016	107.5	2.4
		0.031	107.6	5.2
		0.063	102.2	4.0
		0.125	106.7	1.7
		0.250	104.6	2.0
		0.500	106.0	0.8
		1.000	26.3	1.7
	**Cisplatin**	0.0008	85.0	4.1
		0.0016	77.3	2.4
		0.0031	71.0	2.9
		0.0063	55.9	5.8
		0.0125	33.4	1.6
		0.0250	20.5	1.1
		0.0500	13.9	0.4
		0.1000	12.8	0.5
SK-Mel 28	**5a**	0.008	100.3	0.8
		0.016	100.6	1.0
		0.031	101.5	1.3
		0.063	101.2	0.6
		0.125	101.8	0.8
		0.250	100.0	0.8
		0.500	95.3	3.3
		1.000	71.6	2.4
	**5b**	0.008	118.2	1.4
		0.016	108.6	2.9
		0.031	109.0	3.7
		0.063	104.2	2.6
		0.125	101.7	3.1
		0.250	88.1	3.0
		0.500	69.2	2.6
		1.000	11.1	0.6
	**Cisplatin**	0.0008	93.0	1.0
		0.0016	93.7	2.3
		0.0031	76.9	2.1
		0.0063	58.2	1.8
		0.0125	35.9	0.8
		0.0250	25.0	0.5
		0.0500	15.6	1.3
		0.1000	14.3	1.1

Table S1. *Continuation*

Cell line	Compound	Concentration (mg/mL)	Cell viability (%)	SE (%)[b]
T98G	**5b**	0.008	64.4	5.0
		0.016	63.9	2.9
		0.031	61.6	3.4
		0.063	56.8	2.1
		0.125	62.4	2.3
		0.250	54.2	2.5
		0.500	29.0	2.8
		1.000	7.1	0.5
	**Cisplatin**	0.0008	86.4	3.6
		0.0016	81.9	1.5
		0.0031	66.8	2.9
		0.0063	39.1	2.2
		0.0125	21.4	1.4
		0.0250	12.3	0.2
		0.0500	9.7	0.3
		0.1000	9.3	0.5
U87 MG	**5b**	0.008	107.0	0.9
		0.016	88.8	3.9
		0.031	87.9	4.2
		0.063	86.7	5.1
		0.125	83.5	7.0
		0.250	69.0	7.3
		0.500	44.6	9.2
		1.000	12.4	1.1
	**Cisplatin**	0.0008	68.2	3.4
		0.0016	59.0	2.5
		0.0031	42.8	3.0
		0.0063	21.7	2.2
		0.0125	18.0	1.1
		0.0250	15.0	1.0
		0.0500	10.6	1.9
		0.1000	6.1	0.5

Table S2. Boron Accumulation after Incubation in the Presence of Compound **5b**[a]. $P = 0.95$, $n = 3$

Cell line	Number of cells, $\times 10^6$	Incubation time	Boron content in cells, μg B/10^6 cells
BJ-5ta	1.5	10 min	0.29
			0.44
			0.25
	1.5	30 min	0.13
			0.11
			0.12
	1.8	1 h	0.13
			0.11
			0.11
	1.4	3 h	0.19
			0.19
			0.17
	1.5	6 h	0.13
			0.13
			0.11
	1.4	8 h	0.28
			0.19
			0.23
DU 145	4.0	10 min	0.07
			0.07
			0.09
	4.2	30 min	0.05
			0.06
			0.07
	4.2	1 h	0.09
			0.09
			0.08
	3.0	3 h	0.10
			0.12
			0.10
	5.0	6 h	0.08
			0.09
			0.10
	5.2	8 h	0.07
			0.08
			0.07
MDA-MB-231	0.7	10 min	0.57
			0.37
			0.46
	0.7	30 min	0.36
			0.31
			0.34
	1.8	1 h	0.12
			0.12
			0.12
	0.7	3 h	0.37
			0.37
			0.43
	1.9	6 h	0.12
			0.14
			0.14
	0.6	8 h	0.70
			0.72
			0.58

Cell line	Number of cells, ×10^6	Incubation time	Boron content in cells, μg B/10^6 cells
SK-Mel 28	1.8	10 min	0.38
			0.38
			0.39
	2.0	30 min	0.34
			0.23
			0.43
	1.4	1 h	0.55
			0.52
			0.54
	1.5	3 h	0.44
			0.43
			0.51
	2.0	6 h	0.45
			0.30
			0.40
	1.6	8 h	0.37
			0.37
			0.43
T98G	1.6	10 min	0.17
			0.17
			0.18
	1.0	30 min	0.22
			0.24
			0.26
	1.0	1 h	0.29
			0.21
			0.30
	1.8	3 h	0.17
			0.15
			0.17
	1.4	6 h	0.19
			0.20
			0.20
	1.2	8 h	0.22
			0.22
			0.18
U87 MG	1.6	10 min	0.08
			0.08
			0.09
	1.0	30 min	0.02
			0.01
			0.01
	1.0	1 h	0.05
			0.05
			0.02
	1.8	3 h	0.02
			0.07
			0.06
	1.4	6 h	0.15
			0.16
			0.15
	1.2	8 h	0.07
			0.10
			0.09

[a] Concentration of compound **5b** in nutrient medium was 0.5 mg/mL.
